# Isolation and Characteristics of Extracellular Vesicles Produced by Probiotics: Yeast *Saccharomyces boulardii* CNCM I-745 and Bacterium *Streptococcus salivarius* K12

**DOI:** 10.1007/s12602-023-10085-3

**Published:** 2023-05-20

**Authors:** Kamila Kulig, Katarzyna Kowalik, Magdalena Surowiec, Elzbieta Karnas, Olga Barczyk-Woznicka, Ewa Zuba-Surma, Elzbieta Pyza, Andrzej Kozik, Maria Rapala-Kozik, Justyna Karkowska-Kuleta

**Affiliations:** 1 Department of Comparative Biochemistry and Bioanalytics, Faculty of Biochemistry, Biophysics and Biotechnology, Jagiellonian University, Kraków, Poland; 2 Doctoral School of Exact and Natural Sciences, Jagiellonian University, Kraków, Poland; 3 Department of Cell Biology, Faculty of Biochemistry, Biophysics and Biotechnology, Jagiellonian University, Kraków, Poland; 4 Department of Cell Biology and Imaging, Institute of Zoology and Biomedical Research, Jagiellonian University, Kraków, Poland; 5 Department of Analytical Biochemistry, Faculty of Biochemistry, Biophysics and Biotechnology, Jagiellonian University, Kraków, Poland

**Keywords:** Extracellular vesicles (EVs), Probiotics, Postbiotics, Proteomics, Proteins, THP-1 monocytes

## Abstract

Numerous probiotic microorganisms have repeatedly been shown to produce nanometer-sized structures named extracellular vesicles (EVs). Recently, it has been suggested that similarly to whole microbial cells, EVs produced by probiotics may also demonstrate health benefits to the host, while their application does not involve the risk of infection caused by live microorganisms. In this work, we isolated EVs from two probiotic species originating from different taxonomic domains — yeast *Saccharomyces boulardii* CNCM I-745 and bacterium *Streptococcus salivarius* K12. The diameters of *S. boulardii* EVs were about 142 nm and for *S. salivarius* EVs about 123 nm. For *S. boulardii* EVs, 1641 proteins and for *S. salivarius* EVs, 466 proteins were identified with a liquid chromatography-coupled tandem mass spectrometry and then functionally classified. In both microbial species, metabolic proteins significantly contributed to the cargo of EVs comprising 25% and 26% of all identified vesicular proteins for fungi and bacteria, respectively. Moreover, enzymes associated with cell wall rearrangement, including enzymatically active glucanases, were also identified in EVs. Furthermore, probiotic EVs were shown to influence host cells and stimulate the production of IL-1β and IL-8 by the human monocytic cell line THP-1, and, at the same time, did not cause any remarkable reduction in the survival rate of *Galleria mellonella* larvae in this invertebrate model commonly used to evaluate microbial EV toxicity. These observations suggest that the EVs produced by the investigated probiotic microorganisms may be promising structures for future use in pro-health applications.

**Supplementary Information:**

The online version contains supplementary material available at 10.1007/s12602-023-10085-3.

## Introduction

Numerous microorganisms have been found to produce structures referred to as extracellular vesicles (EVs), or sometimes distinctively called membrane vesicles (MVs) and outer membrane vesicles (OMVs) for Gram-positive and Gram-negative bacteria, respectively [1, 2]. These are nanometer-sized spherical structures, surrounded by a lipid bilayer, containing a wide range of different cargo molecules, including nucleic acids, proteins, peptides, toxins, quorum sensing molecules, phospholipids, polysaccharides, and other compounds protected from the external environment and employed in intercellular communications [1, 3]. Recently, it has been proposed that similarly to live cells of probiotics — live organisms that contribute to the improvement of host health [4–7], also their vesicles may have a pro-health effect, while their use does not carry the risk of infection that could be caused by complete microbial cells [1, 8, 9].

The positive effect of probiotics on human health during inflammation-associated diseases or at risk of microbial infection may be the result of their influence on the host immune system by modulating its responses and/or the impact of probiotic cells on the resident microbiota and invading pathogens [6]. In the latter case, probiotics can act through antimicrobial compounds that inhibit pathogen growth and may compete directly with pathogens by attaching to host cell receptors or by adhesion to the surface and colonization of a particular niche [10]. The well-known probiotic yeast strain is *Saccharomyces boulardii* CNCM I-745, which demonstrates a valuable effect for the host against infections caused by pathogenic bacteria such as *Salmonella*, *Shigella*, *Escherichia coli*, and *Clostridium difficile*, some viruses and fungi — *Candida albicans* [11]*.* The mechanisms of action of the probiotic yeast include direct binding to bacteria followed by their elimination or the effect on bacterial virulence factors such as toxins or lipopolysaccharide (LPS); additionally, it was also shown to possess anti-inflammatory, immunomodulatory, and reconstructive activity in the intestinal mucosa [12]. One of the eminent bacterial strains considered as probiotic is *Streptococcus salivarius* K12, the safety and effectiveness of which have been supported by numerous studies [13, 14]. *S. salivarius* K12 produces bacteriocin-like inhibitory substances (BLIS), including salivaricin A and B lantibiotics, as well as different other, and yet uncharacterized bacteriocins, which regulate the overgrowth of different pathogens, thus preventing the onset of dangerous infections [15]. The antimicrobial activity of *S. salivarius* K12 has been successfully exploited against bacteria related to upper respiratory tract diseases such as *Streptococcus pyogenes* and *Streptococcus pneumoniae* [16–18]. Furthermore, they also demonstrated antibiofilm activity against *Streptococcus mutans* or *Staphylococcus hominis* [19].

Vesicles produced by probiotics currently attract great interest regarding their applicability in the creation of new vaccine technology, as well as in the novel approach to antibacterial and antifungal treatment, which is particularly important due to the rapid propagation of resistance of microbial pathogens to conventional antibiotics [3, 20]. This is a very promising area, and since the potential functionalities of vesicles are closely related to their structure and content, the identification of the latter becomes crucial to arranging the effective use of probiotic vesicles for health-promoting purposes. Therefore, in this work, we present a method of efficient vesicle isolation and preliminary characterize extracellular vesicles and membrane vesicles, produced by two promising, albeit diverse, probiotic microorganisms, eukaryotic *S. boulardii* CNCM I-745, and prokaryotic *S. salivarius* K12, respectively; however, for simplification, we will refer to both types of vesicles further in the text as EVs.

## Materials and Methods

### Fungal and Bacterial Growth Conditions

*S. boulardii* CNCM I-745 (Biocodex, Gentilly, France) was cultured for 18 h in 20 ml of liquid YPD medium (1% (w/v) yeast extract, 2% (w/v) soybean peptone and 2% (w/v) glucose, Sigma-Aldrich, St. Louis, MO, USA) at 30 °C in an orbital shaker MaxQ 6000 (Thermo Fisher Scientific, Waltham, MA, USA) with a rotation speed of 170 rpm. *S. salivarius* (Andrewes and Horder) strain K12 (ATCC BAA-1024) was purchased from American Type Culture Collection (Manassas, VA, USA). Bacterial cells were cultured for 24 h in 20 ml liquid brain heart infusion (BHI) medium (Sigma-Aldrich) at 37 °C in an atmosphere of 5% CO_2_, without shaking. To prepare cultures on appropriate agar plates (YPD broth with 1.5% (w/v) agar for yeasts and BHI broth with 1.5% (w/v) agar for bacteria), cells were centrifuged for 5 min (3000 × *g* for S*. boulardii* and 5000 × *g* for *S. salivarius*); the cell pellet was suspended in sterile Dulbecco’s phosphate-buffered saline (DPBS) containing 8.1 mM sodium phosphate, 136.9 mM sodium chloride, 2.68 mM potassium chloride, and 1.47 mM potassium phosphate, pH 7.50 ± 0.30 (Biowest, Nuaillé, France); and the number of cells was estimated using the OD measurement at 600 nm and 3 × 10^8^ yeast cells or 3 × 10^9^ bacterial cells were spread on the agar plates and cultured for 24 h at 37 °C, additionally in an atmosphere of 5% CO_2_ in the case of *S. salivarius*.

### Extracellular Vesicle Isolation

Yeast or bacterial cells were carefully scratched from agar plates with a polystyrene, sterile inoculating loop, avoiding damaging the YPD/BHI agar surface, transferred to the Eppendorf tube, suspended in 1 ml of sterile DPBS, pH 7.50 ± 0.30, and gently stirred with the loop for 10 min. Then, after centrifugation twice at 4000 × *g* for 15 min at 4 °C, performed to remove cells and cell debris, the supernatant with EVs was collected and filtered using an Ultrafree-CL Centrifugal Filter (Merck, Darmstadt, Germany) with a pore size of 0.65 μm (for yeasts) or 0.22 μm (for bacteria) to remove any residual cell remnants. Then supernatants were ultracentrifuged at 4 °C for 1 h in polycarbonate thick wall centrifuge tubes (13 × 64 mm) with 13-mm-diameter Delrin tube adapters, using a fixed-angle type 60 Ti Rotor in an Optima LE-80 K Ultracentrifuge (Beckman Coulter, Brea, CA, USA) with a relative centrifugal field of 144,000 × *g* (*k* factor 112). The obtained EVs were transferred in 200 μl of DPBS buffer filtered through a 0.22-µm filter to Eppendorf tubes and stored at − 80 °C for further use.

### TEM Imaging and NTA Measurements of EVs

To visualize EV samples, a negative stained transmission electron microscopy (TEM) was used with formvar-coated, 300-mesh copper grids prepared for each EV sample using 2% (w/v) uranyl acetate (Chemapol, Prague, Czech Republic) as described in a previous work [21]. Imaging of EVs was performed using a JEOL JEM-2100 HT transmission electron microscope (JEOL, Tokyo, Japan).

Size and concentration measurements were performed using the nanoparticle tracking analysis (NTA) and NanoSight NS300 system with camera type sCOS, laser Blue488, and NTA software Version 3.4 (Malvern Instruments, Malvern, UK). Measurements were performed at 25 °C in DPBS buffer containing 8.1 mM sodium phosphate, 136.9 mM sodium chloride, 2.68 mM potassium chloride, and 1.47 mM potassium phosphate, pH 7.30 ± 0.30 filtered through a 0.22-µm filter (Lonza, Basel, Switzerland), and the samples were recorded three times for 60 s with camera level of 12 and the threshold parameter set at 2 [21].

### Protein and Phospholipid Concentration Measurements

The protein concentration in the EV samples was measured with *o-*phthalaldehyde (OPA; Sigma) in three biological replicates, and the fluorescence intensity was measured with excitation and emission at 340 nm and 455 nm, respectively [22]. Phospholipid concentration was determined using the MAK122 Phospholipid Assay Kit (Sigma) in three biological replicates [23], precisely following the manufacturer’s instructions. The absorbance at 570 nm (MAK122) and the fluorescence intensity (OPA) were determined using a Synergy H1 microplate reader (BioTek Instruments, Winooski, VT, USA).

### Proteomic Analysis of EVs Using Liquid Chromatography-Coupled Tandem Mass Spectrometry (LC–MS/MS)

The proteomic analysis of EVs was performed as described in detail in the previous work [24]. Briefly, the EV sample was prepared with a total amount of proteins equal to 11 µg, suspended in 100 µl of 100 mM Tris–HCl buffer, pH 7.60 ± 0.10, with 1% (w/v) sodium dodecyl sulfate. The prepared sample was sonicated using UP50H Compact Lab Homogenizer (amplitude 80%, cycle 0.5, 50 W, 30 kHz; Hielscher Ultrasonics, Teltow, Germany) in four cycles of 30 s. Subsequently, the samples were shaken for 5 min at 95 °C, centrifuged for 15 min at 12,000 × *g*, and the obtained supernatants were precipitated during overnight incubation at − 20 °C by adding trichloroacetic acid in one volume to four volumes of the prepared sample. Then, the samples were centrifuged at 10,000 × *g* for 15 min at 10 °C and washed twice with ice-cold acetone, and the obtained pellet was suspended in 100 µl of 10 mM HEPES buffer, pH 8.50 ± 0.20. Subsequent steps were performed according to the protocol described by Surman et al. [25], briefly, using paramagnetic bead technology based on single-pot solid phase-enhanced sample preparation [26] and digestion with 30 µl of 0.027 µg/µl Trypsin/Lys-C Mix (Promega, Mannheim, Germany). Then, obtained peptides were identified by MS/MS analysis using UltiMate 3000 RSLCnano System coupled with Q-Exactive mass spectrometer (Thermo Fisher Scientific) with DPV-550 Digital PicoView nanospray source (New Objective, Woburn, MA, USA). The obtained RAW files were processed by the Proteome Discoverer platform (v.1.4, Thermo Fisher Scientific) and searched using a locally installed MASCOT search engine (v.2.5.1, Matrix Science, London, UK). The NCBI database was used with the taxonomy restrictions: *Saccharomyces boulardii* (11063 sequences) and *Streptococcus salivarius* (114052 sequences). The following parameters were applied: fixed modification, cysteine carbamidomethylation; variable modifications, methionine oxidation; precursor mass tolerance, 10 ppm; fragment mass tolerance, 20 mmu. The mass spectrometry proteomics data have been deposited to the ProteomeXchange Consortium via the PRIDE partner repository [27] with the dataset identifier PXD039137 (*S. boulardii*) and PXD039138 (*S. salivarius*).

### Quantitative Assessment of Cytokines Produced by THP-1 Cells

The human monocytic cell line THP-1 (Sigma) was cultured in RPMI 1640 medium (Biowest) supplemented with 10% fetal bovine serum (FBS) (Thermo Fisher Scientific) at 37 °C in the atmosphere of 5% CO_2_ and 95% humidity. Differentiation from monocytes to macrophage-like cells was performed by treating cells with 10 ng/ml of phorbol 12-myristate 13-acetate (PMA) added to medium with 100 U/ml penicillin and 100 mg/ml streptomycin (both from Biowest) for 48 h (with medium exchange after 24 h). After differentiation, the medium was replaced with fresh medium without PMA for 3 h, but still with 5% (v/v) FBS, 100 U/ml penicillin, and 100 mg/ml streptomycin, and then the macrophage-like cells (5 × 10^5^ cells per well of a 24-well microplate) were incubated with EVs for 24 h, at 37 °C. After stimulation of THP-1 cells by EVs with a cell-to-EV ratio of 1:100000 (for fungal EVs) or 1:50000 (for bacterial EVs), cells were discarded by centrifugation (1000 rpm, 5 min), and supernatants were collected for further analysis of cytokine production. Four independent experiments were performed. Unstimulated cells were the negative control, while cells stimulated with LPS (100 ng/ml; Sigma-Aldrich) were the positive control. The levels of selected cytokines — IL-1β, IL-8, tumor necrosis factor α (TNF-α) — produced by macrophage-like cells were measured using Human IL-1β ELISA Set II, Human IL-8 ELISA Set, and Human TNF ELISA Set kits (BD OptEIA) strictly following the manufacturer’s instructions. To analyze the statistical significance versus control, an unpaired *t* test was performed with GraphPad Prism software version 7.0 (GraphPad Software, La Jolla, CA, USA).

### Measurement of Glucanase Activity

Glucanase activity was measured with colorimetric method with the laminarin (β-(1,6)-intrachain linked (1,3)-β-D-glucan) (Sigma-Aldrich) as a substrate using dinitrosalicylic acid (DNS) assay allowing the detection of released reducing sugars [28]. D-glucose solutions were used for calibration. Briefly, 5 × 10^9^ EVs from *S. boulardii* and *S. salivarius* were suspended in 50 µl of DPBS, and 125 µl of 1% (w/v) laminarin solution in sodium acetate buffer, pH 5.0 ± 0.10, were added for further incubation for 30 min at 37 °C. Westase Enzyme (Takara Bio Inc. Shiga, Japan) was used as a positive control at the concentration of 1 g/ml (5 mg per sample). For the measurement of the level of released reducing sugars, 750 µl of DNS was added to each sample with further incubation for 5 min at 100 °C. Absorbance was measured at 540 nm using a Synergy H1 Microplate Reader after transferring 200 µl of the sample to the 96-well microplate (Sarstedt, Nümbrecht, Germany). The absorbance signal obtained for the substrate sample without the enzyme was subtracted from the rest of the samples.

### Evaluation of EV In Vivo Safety Using *Galleria mellonella* Invertebrate Model

The moth *Galleria mellonella* larvae were used to assess the potential toxicity of fungal and bacterial EVs according to the previously published protocols [29]. Larvae in their final instar stage (10 per group) were randomly chosen for the experiment and then inoculated in the last left proleg with 10 µl of the solution of fungal or bacterial EVs in sterile DPBS buffer, pH 7.50 ± 0.30 (Biowest) using a 10 μl Hamilton syringe (Merck). DPBS buffer served as a control for injection. The caterpillars were further incubated at 37 °C, and the number of dead larvae was scored daily; they were considered dead when they did not show movement in response to touch. The experiment was carried out in three biological replicates, and the averaged results are presented. The survival curves of EV-treated and control animals were compared using the Mantel-Cox log-rank test with GraphPad Prism 7.0. A value of *p* < 0.05 was considered significant.

## Results

Fungal and bacterial EVs were collected after 24 h of cell growth on the agar plates with cultures started from the defined cell number. Cells were scratched from the surface of the agar and then suspended and gently stirred in DPBS buffer. Subsequently, serial centrifugation steps were completed before ultracentrifugation of the samples and isolation of EVs. This served to separate whole microbial cells from preparations containing EVs only, which was also supported by the filtration of the sample before ultracentrifugation step through the filters with pore sizes smaller than the size of microbial cells.

**Fig. 1:**
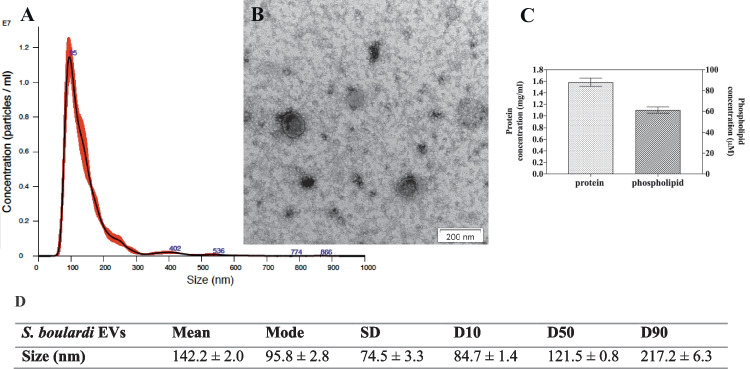
Characteristics of *S. boulardii* EVs. NTA particle size distribution analysis (**A**) and TEM image of EVs (**B**); representative histograms of the average size distribution from three measurements of a single sample (black line). The numbers show the maxima of particular peeks, and widened areas indicate the standard deviation (SD) between measurements. Average protein and phospholipid content in *S. boulardii* EVs (**C**). The size parameters of the EVs measured by NTA; factors D10, D50, and D90 mean that 10%, 50%, and 90% of the EV population had a diameter of less than or equal to the given value (**D**)

**Fig. 2:**
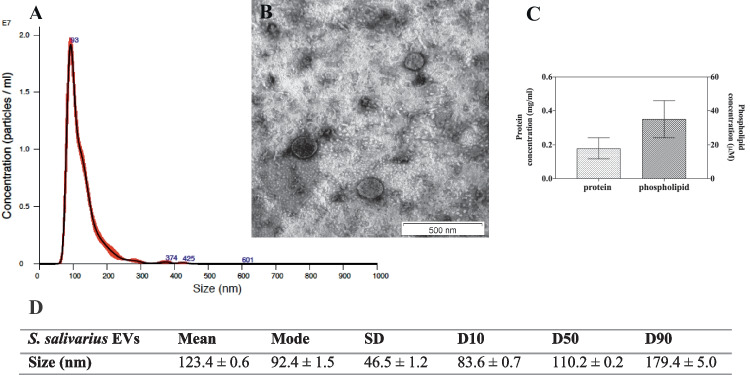
Characteristics of *S. salivarius* EVs. NTA particle size distribution analysis (**A**) and TEM image of EVs (**B**); representative histograms of the average size distribution form three measurements of a single sample (black line). The numbers show the maxima of particular peeks, and widened areas indicate the standard deviation (SD) between measurements. Average protein and phospholipid content in *S. salivarius* EVs (**C**). The size parameters of the EVs measured by NTA; factors D10, D50, and D90 mean that 10%, 50%, and 90% of the EV population had a diameter of less than or equal to the given value (**D**)

To characterize EV size and concentration, NTA analyses were performed, together with TEM imaging demonstrating the spherical structures that varied in size and surrounded by a lipid bilayer, both for yeasts and bacteria (Figs. 1A, B and 2A, B, respectively). The average sizes of the particles were measured, and the diameters of the *S. boulardii* EVs were about 142 nm (Fig. 1D) and for *S. salivarius* EVs about 123 nm (Fig. 2D).

The determination of the number of particles showed that the fungal culture initiated by 3 × 10^8^ cells seeded on the YPD agar plate allowed to isolate 2.303 ± 0.014 × 10^8^ particles and the bacterial culture initiated by 3 × 10^9^ cells seeded on the BHI agar plate allowed to isolate 1.580 ± 0.001 × 10^9^ particles. The concentrations of protein and phospholipid, as one of the main components of EVs, were determined in the obtained EV-containing samples. The average protein and phospholipid concentration for *S. boulardii* EVs was 1.6 mg/ml and 60 μM, respectively (Fig. 1C), and for *S. salivarius* EVs 0.2 mg/ml and 35 μM, respectively (Fig. 2C). For yeast EVs, concentrations were of a similar order of magnitude to those in previous studies when EVs of selected *Candida* pathogenic fungi were isolated and characterized [21, 24]. The detected *S. salivarius* vesicular protein concentration was comparable to that previously reported for EVs produced by *Streptococcus pneumoniae* and other probiotic bacterium, *Lactiplantibacillus plantarum* [30, 31].

**Fig. 3:**
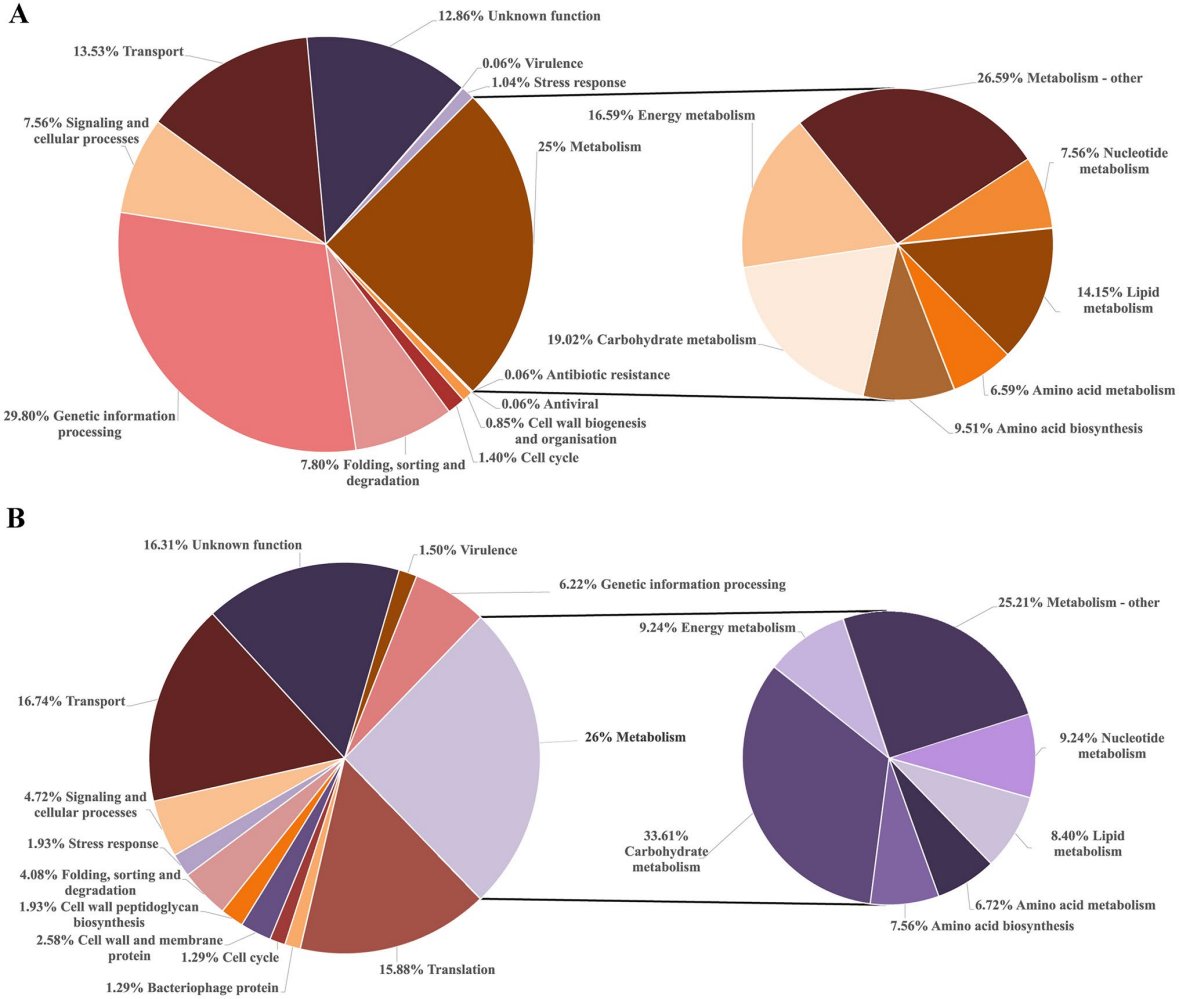
Proteins identified for *S. boulardii* (**A**) and *S. salivarius* (**B**) EVs grouped by their function

To identify the protein content of the *S. boulardii* and *S. salivarius* EVs, LC–MS/MS analyses were performed. Proteomic identification revealed the presence of 1641 proteins for *S. boulardii* EVs and 466 proteins for *S. salivarius* EVs (Supplementary Tables 1 and 2, respectively), which were assigned different functions (Fig. 3) with the UniProt protein database (https://www.uniprot.org/) [32] and InterPro protein families database (https://www.ebi.ac.uk/interpro/) [33].

**Fig. 4:**
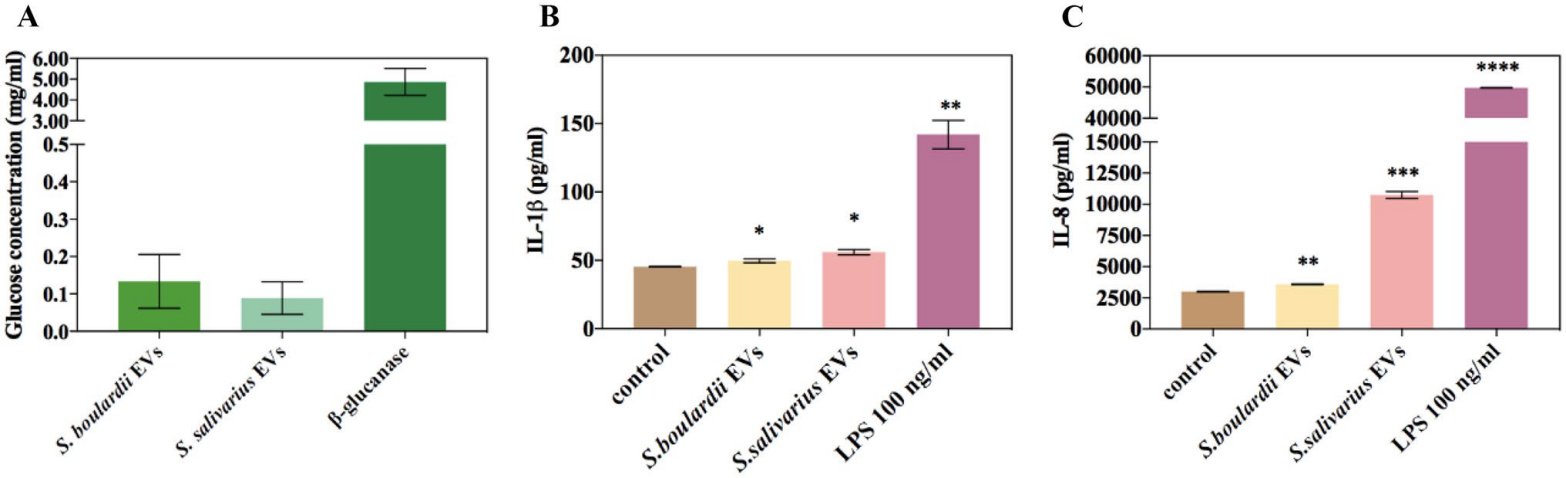
Glucanase activity measured with laminarin as a substrate (**A**) and analysis of cytokine IL-1β (**B**) and IL-8 (**C**) production by THP-1 cells stimulated with EVs of *S. boulardii* and *S. salivarius* EVs. A representative result of four independent experiments is presented. The statistical significance versus control was analyzed by performing an unpaired *t* test with GraphPad Prism software version 7.0 (GraphPad Software, La Jolla, CA, USA). The statistical significance levels were marked with ns for *p* ≥ 0.05, * for *p* < 0.05, ** for *p* < 0.01, *** for *p* < 0.001, and **** for *p* < 0.0001

For both microorganisms, one of the most abundant categories was proteins related to cellular metabolism, which accounted for 25% of all identified vesicular proteins for fungi (predominantly proteins from carbohydrate metabolism with 19.2% of representatives of this group) (Fig. 3A) and 26% for bacteria, also with the predominance of carbohydrate metabolism proteins comprising over 33% of this protein set (Fig. 3B). Additionally, for *S. boulardii* EVs, proteins involved in genetic information processing were 29.8% of all identified proteins, constituting the most numerous group; more than 13% of the proteins were molecules involved in transport, about 7.8% in protein folding, sorting and degradation, over 7% in cellular signaling, 1.4% in cell cycle, approximately 1% in stress response, and less than 1% in cell wall biogenesis (Fig. 3A). The identification of numerous fungal proteins associated with carbohydrate metabolism and rearrangement of yeast cell wall polysaccharides (Supplementary Table 1) prompted us to verify whether isolated EVs may exhibit some glucanase activity [34], which was corroborated by an enzymatic assay that showed glucose release when EVs were incubated with laminarin used as a substrate for glucanases (Fig. 4A).

In the case of *S. salivarius* EVs, almost 17% were proteins involved in transport, approximately 16% in translation, more than 6% in genetic information processing, more than 4% in cellular signaling and the similar portion in protein folding and degradation (Fig. 3B). Interestingly, the same percentage (1.93%) were bacterial vesicular proteins involved in the stress response and cell wall peptidoglycan biosynthesis; also 2.58% of proteins were cell wall and membrane proteins (Fig. 3B).

The ability of microbial EVs to modulate host immune system responses is well documented [35]. In the present study, we focused on the EVs impact on the production of cytokines by human monocytic cell line THP-1 cells differentiated into macrophage-like cells. The analysis of the level of cytokines such as TNF-α, IL-1β and IL-8 was carried out in four biological replicates, and the representative results are presented in Fig. 4. There has been observed a statistically significant increase in the production of IL-1β (Fig. 4B) and IL-8 (Fig. 4C) for both *S. boulardii* and *S. salivarius* EVs when compared to the control, which were untreated cells. The analysis of TNF-α levels demonstrated that this cytokine was not produced in a significantly different amount than in the control when cells were stimulated by both types of probiotic EVs (data not shown).

**Fig. 5:**
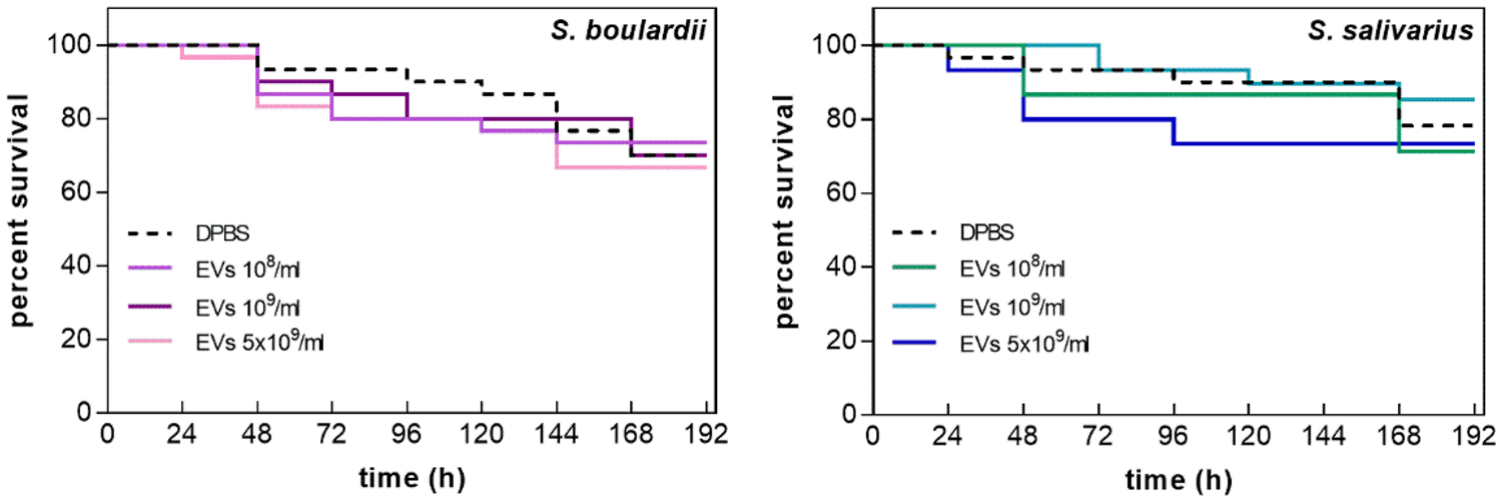
The survival curve of *Galleria mellonella* larvae after the injection of *S. boulardii* or *S. salivarius* EVs or DPBS as a control

The impact on the survival of the model organism *Galleria mellonella* after EV injection was tested for 8 days using three concentrations of fungal or bacterial EVs per ml – 1 × 10^8^, 1 × 10^9^, and 5 × 10^9^ prepared in DPBS buffer (Fig. 5).

For the highest concentration of EVs, the largest decrease in survival was observed in the shortest period of time; however, the differences between survival curves were not statistically significant, that is, the mortality of larvae after injection was not considerably different from the mortality after injection of DPBS buffer alone.

## Discussion

In the current work, for the first time to the best of our knowledge, EVs released by the probiotic microorganisms *S. boulardii* CNCM I-745 and *S. salivarius* K12 were isolated and characterized in terms of size, protein content, and selected aspects of impact on the host. The use of EVs from probiotic organisms — defined as postbiotics — is currently widely considered in the aspect of the pro-health effect on humans, also in the context of alleviating diseases related to inflammatory conditions and infections caused by various pathogens, such as *Clostridium difficile*, *Salmonella* Typhimurium, or *Helicobacter pylori* [1, 8]. The beneficial effect of probiotic EVs may be manifested by modulating the activity of the host immune system, strengthening the epithelial barrier, and direct harmful effect on pathogens [9]. At the same time, the use of EVs instead of whole cell microorganisms avoids the potential risk of infection caused by the probiotic, which is not common, although some cases were reported [36, 37].

Most of the fungal vesicles isolated previously from *S. cerevisiae* cultures were in the size range of 100 nm to 200 nm; therefore, the measurements performed herein for *S. boulardii* vesicles are consistent with those studies [38, 39], and since the strain *S. boulardii* CNCM I-745 is very closely related to *S. cerevisiae* [11], specific comparisons between the strains are mostly justified. The average size range of EVs isolated from *S. salivarius* K12 was comparable to EVs produced by other bacteria of the *Streptococcus* genus, as the average size reported for *S. equi* EVs ranged from 50 to 150 nm [40] and from 130 to 160 nm for EVs of *S. pneumoniae* [30]. For other probiotic-derived EVs, the sizes ranged from 50 to 800 nm for *L. plantarum* EVs and from 20 to 200 nm for *L. rhamnosus* EVs, *L. casei* EVs and *L. brevis* EVs isolated from culture supernatants [31], and approximately 90 nm, 130 nm, and 126 nm for *L. crispatus*, *L. gasseri* [41], and *Bifidobacterium longum* [42], respectively.

The proteomic analysis of EVs produced by strains of *S. boulardii* and *S. salivarius* investigated in this study revealed the most represented functional groups of vesicular proteins. Among the proteins identified for *S. boulardii* EVs, the group of proteins involved in cell wall organization has been distinguished, and several fungal glucanases were identified therein, including endo-beta-1,3-glucanase Bgl2, cell wall protein like glucanase Scw11, cell wall protein Scw10 with similarity to glucanase, and major cell wall exo-1,3-beta-glucanase Exg1. Their enzymatic activity in the EVs sample was confirmed with straightforward colorimetric assay with laminarin as a substrate. Additionally, chitin synthases were also identified in this study among yeast vesicular proteins, indicating the potential involvement of *S. boulardii* EVs in cell wall polysaccharide remodeling and their role in cell wall dynamics, similarly as it was indicated for EVs of *S. cerevisiae* [43, 44]. As well, polysaccharide-hydrolyzing enzymes may participate in the traversal of EVs through the cell wall, causing partial hydrolysis of its components and release of the vesicle structure outside the cell [45]. Furthermore, glucanase activity may also affect fungal recognition by human immune cells equipped with receptors for glucan detection, like Dectin-1 [46, 47]. However, assumptions about the functionality of these enzymes in the *S. boulardii* vesicles require further detailed research.

Within the identified *S. boulardii* vesicular proteins, there were numerous proteins attributed to different types of cellular membranes, and this group included, among others, several plasma membrane proteins, endoplasmic reticulum transmembrane protein Yet1, Golgi apparatus membrane proteins Tvp15 and Tvp18, Sec9 t-SNARE protein required for secretory vesicle-plasma membrane fusion, Pep12 target membrane receptor (t-SNARE), and Sft2 tetra-spanning membrane protein found mostly in the late Golgi, suggesting the complexity of the process of EVs formation, diverse structural biogenesis, and high heterogeneity of vesicle group [38, 48]. Among EV-related yeast proteins, there were also proteins involved in the formation of eisosome domains, including eisosome core components Lsp1 and Pil1, and component required for proper eisosome assembly Eis1, which are important for the organization of the plasma membrane and cell wall morphogenesis [49]. This protein family also includes Sur7 protein, which in the case of *Candida albicans* is currently considered as a marker of fungal EVs [50]; in this study, this particular protein was identified for *S. boulardii* as plasma membrane protein with GenBank accession number KQC41723.1.

Considering the proven probiotic properties of the investigated *S. boulardii* strain CNCM I-745 [51], one of the beneficial proteins found in yeast EVs might be spermidine synthase, responsible for the production of polyamine spermidine, which in the intestine exhibits the anti-inflammatory effect on innate and adaptive immunity, and displays epithelial cell renewal properties [52]. Furthermore, Buts et al. [53] demonstrated that *S. boulardii* secreted leucine aminopeptidase — zinc-binding metalloprotease from M1 family of peptidases — facilitating digestion of proteins and peptides in the intestine, while in this study aminopeptidase of similar characteristics and specificity has been identified in EVs produced by yeasts. The enzyme invertase, which hydrolyzes sucrose into fructose and glucose, thus playing a supportive role in the digestion of saccharides in the intestine was also found in *S. boulardii* EVs herein. The presence of these enzymes might be helpful to the human host if the digestive system is immature, there are some enzyme deficiencies, and inflammatory conditions or infection are present [51]. Additionally, also alkaline phosphatase was found in *S. boulardii* vesicles in this study, which is an enzyme indicated previously as capable to inhibit toxicity of *E. coli* surface LPS by its dephosphorylation [54].

In the case of *S. salivarius* EVs, several identified proteins belong to the superfamily of ATP binding cassette (ABC) transporters, which exploit the energy from binding and hydrolysis of ATP to transport different substrates through the cellular membrane, thus might be involved in a wide variety of functions in bacteria, including transport of ions and nutrients, drug resistance, production of extracellular polysaccharides and glycoconjugates, and secretion of various proteins, toxins, and bacteriocins, many of which influence the contact of bacteria with the host [55, 56]. However, in this work, only a few of these *S. salivarius* proteins could be directly attributed to specific functions, including iron-siderophore ABC transporter, amino acid ABC transporter, zinc ABC transporter, or spermidine/putrescine import ATP-binding protein PotA, while for others, the exact functions remained unspecified and were only generally classified as ABC transporters. Among the identified *S. salivarius* proteins, there were also numerous proteins equipped with signal sequences, i.e., KxYKxGKxW signal peptide domain-containing protein, YSIRK-type signal peptide-containing protein, or YSIRK signal domain/LPXTG anchor domain surface protein, and the representatives of the latter group have been previously indicated as bacterial surface adhesins, involved in binding of *S. salivarius* cells to the intestinal epithelial cells [57]. Among proteins present in EVs of *S. mutans*, KxYKxGKxW signal peptide-containing protein has also been found [58]. Additionally, several proteins involved in bacterial cell wall biosynthesis and related to resistance to antibiotics [59], such as penicillin-binding proteins — penicillin-binding protein PBP2A, penicillin-binding protein 2X, penicillin-binding protein PBP1B, and cell wall-active antibiotic response protein — were identified in bacterial vesicles herein, suggesting the participation of these structures in the synthesis and rearrangement of streptococcal peptidoglycan.

Furthermore, several proteins with supportive function representing the probiotic activities of *S. salivarius* were identified in bacterial EVs, including urease — alpha and gamma subunit, and urease accessory protein UreG [60], additionally also bacteriocin immunity protein, a key component of the bacterial system important to elicit optimal antimicrobial activity [61], which was also identified in other probiotic-derived EVs, e.g., in *L. acidophilus*, and was considered important also in the regulation of immune response and bacterial adhesion [62]. Moreover, also L-lactate dehydrogenase was identified in *S. salivarius* EVs cargo, the enzyme crucial for lactic acid bacteria to produce this compound, important in medical applications, agriculture, and food and chemical industries [63].

Additionally, also the vesicular presence of glucosyltransferases, which are important factors influencing aggregation of *S. salivarius* cells [64], has been indicated herein. The glucosyltransferases are enzymes involved in the processes of maturation and modification of biofilm structures and biosynthesis of extracellular compounds — glucans, glycoproteins, cell wall polysaccharides, peptidoglycan, and lipoteichoic acid. Glucosyltransferases are represented in *S. salivarius* genome by six genes *Gft1*-*Gft6* [65]. This group of enzymes is also highly represented in membrane vesicles of *S. mutans* [58].

Interestingly, it has been previously demonstrated that EVs of probiotic bacteria *L. casei* BL23 may carry phage proteins [66] that could be transferred to other bacterial species colonizing the same niche and thus potentially could serve as a new aspect useful in the treatment of bacterial infections [1]. This work also identified six proteins associated with bacteriophages in *S. salivarius* K12 EVs, including phage holin or site-specific integrase.

The analysis of molecules derived in EVs of *S. equi*, pathogenic bacterium causing respiratory infection in horses, revealed a few candidates for potential vaccine. Among them, there were such proteins as penicillin-binding protein 1A, several ABC transporter substrate-binding proteins, and other enzymes [40]. In the current study, in the identified protein content of EVs of *S. salivarius* K12, the presence of about 40 ABC transporter proteins and several penicillin-binding proteins was revealed as abovementioned, making *S. salivarius* EVs a potential vaccine candidate. Recently, also *S. cerevisiae* EVs were found as an attractive source for novel vaccine material applied for immune cell maturation [2]; and interestingly, they contained heat shock protein 70 (Hsp70), while similar proteins were also found in *S. salivarius* K12 EVs in this study.

As presented in many other studies, the immune response of different types of host cells varies when they are in contact with whole probiotic cells or with isolated probiotic EVs. In our present study, in consistency with some published results and in contrary to other, there was observed a proinflammatory response of macrophage-like cells after contact with yeast and bacterial EVs; however, it should be bear in mind that the observed response is highly dependent on the probiotic strain used, the origin and type of host cells stimulated, the initial conditions, and the prior presence of inflammation or infection. For *S. boulardii* cells, the stimulation of monocyte-derived dendritic cells for production of multiple cytokines was observed, and the levels of IL-1β, IL-12, IL-10, IL-6, and TNF-α were higher after contact with probiotic yeast; moreover, the obtained results were comparable when live yeast cells and UV-irradiated and heat-treated cells were used [67]. In other studies, it was also shown that some commercial and isolated strains of *S. boulardii* can affect the immune response by increasing the production of proinflammatory cytokines IL-6, IL-12, IL-1β, TNF-α, or IL-8 chemokine by dendritic cells [37]. In the case of probiotic bacteria, the analysis performed previously showed that an immunomodulatory effect of *S. salivarius* can differ among closely related species or strains and that response was also altered by the type of the human cells used [68, 69]. For the epithelial cells after contact with *S. salivarius* K12 cells, the upregulation of genes involved in the homeostasis was demonstrated, similarly to the immunosuppression and inhibition of bacterial pathogens activity, suggesting that these probiotic cells play an important role in reducing inflammation [70]. In contrary, in other studies, the stimulation of monocyte-derived dendritic cells with *S. salivarius* cells showed an increase in the production of IL-8, TNF-α, and IL-1β [71]. The U937 cells differentiated to macrophages also showed increased levels of IL-10 and TNF-α genes after contact with *S. salivarius* cells [72]. As reported by Kaci et al. [73], supernatants from culture of *S. salivarius* with the addition of TNF-α inhibited the activation of NF-κB signaling pathway, and the effect of such inhibition was the absence of the IL-8 production. The authors suggested that obtained results were related to the presence of undefined low-molecular-weight metabolite secreted by *S. salivarius* cells [73].

The effects of EVs derived from probiotics on human cells are also very diverse. In the case of *S. cerevisiae* EVs, it was demonstrated that they significantly increased the production of proinflammatory TNF-α and IL-6 by mouse macrophage-like RAW264.7 cells and mouse dendritic DC2.4 cells [2]. Kurata et al. [31] reported that *L. plantarum* EVs stimulated innate and acquired immunity, by inducing production of the both, pro-inflammatory and anti-inflammatory cytokines, by RAW264.7 cells, including IL-1β, IL-6, and IL-10, that elicited response of the innate immune system and by inducing production of IFN-ɣ and IL-12p70 as part of the acquired immune system. The observed activation of the host immune system was noticed as a consequence of the recognition of specific ligands displayed at the surface of the EVs by pattern recognition receptors (PRRs) and activation of the transcription factor NF-κB. The most important in activation of host immune cells by lactobacilli EVs seemed to be TLR1/2 and TLR2/6 receptors. Among identified bacterial proteins, there were lipopeptides which were indicated as potential TLR2 ligands in EVs of *L. plantarum.* Moreover, EVs of *L. plantarum* appeared to induce different response of the host immune system than complete bacterial cells [31]. In other studies, *L. plantarum* EVs were demonstrated to induce monocyte-to-macrophage transition and polarization of the macrophages to the M2b state in vitro, and additionally, the increase in mRNA expression of IL-10, IL-1β, and IL-6 genes was observed [1, 74]. The EVs of *B. longum* stimulated macrophage-like cells RAW264.7 and dendritic cells DC2.4 to produce TNF-α and IL-6, the same as *L. plantarum* EVs, although the response by both human cells lines for EVs of *B. longum* was higher than for EVs of *L. plantarum* in the case of both released pro-inflammatory cytokines. The performed analysis showed that TLR2 receptor plays a crucial role in the activation of host immune system by probiotic-derived EVs. Moreover, in the production of cytokines two signaling pathways, JNK/MAPK and NF-κB performed important function [40]. After stimulation with *L. rhamnosus* EVs of the mouse macrophage-like cells RAW264.7, the decrease of TNF-α, IL-1β, and IL-6 was observed [1, 75]. For *Clostridium butyricum* EVs, the stimulation of RAW264.7 cells and DC2.4 cells resulted in increased levels of TNF-α and IL-6 [1, 76]. In our current work, we reported the increase of IL-1β and IL-8 production by THP-1 cells differentiated to macrophage-like cells and the lack of production of TNF-α, suggesting that both types of EVs, those released by *S. boulardii* CNCM I-745 and by *S. salivarius* K12, are capable of modulating the immune response of the host cells, although probably they do not trigger acute inflammatory process. Therefore, we hypothesize that they may have an immunostimulatory effect and thus be beneficial in the prevention and control of infections.

The potential of using EVs as postbiotics for health benefits also encouraged us to test their toxicity in an in vivo system. In this study, we analyzed the effect of *S. boulardii* CNCM I-745 and *S. salivarius* K12 EVs on survival of model organism *Galleria mellonella*, and no significant post-injection mortality of larvae was observed at the concentrations and conditions used. In analyses performed by Moman et al. [77], injection with *S. salivarius* K12 cells did not show a significant mortality effect on *G. mellonella* larvae. However, in the case of injections with cells from different *S. boulardii* isolates, some lethality was observed after the inoculation with probiotic products [37].

In conclusion, EVs isolated from the two probiotic strains demonstrated quite comparable impacts on host cells, indicating rather an immunostimulatory effect. They also seem to be non-toxic in an in vivo model, and the identification of their plentiful protein content may not only indicate the potential mechanisms of their biogenesis or traversal through the cell wall, but also allows to form assumptions about their functionality.

### Supplementary Information

Below is the link to the electronic supplementary material.

Supplementary file1 (PDF 2390 kb)

Supplementary file2 (PDF 741 kb)

## Data Availability

The datasets generated and analyzed during the current study are available in the Cracow Open Research Data Repository, 10.57903/UJ/TKVZSG. The mass spectrometry proteomics data have been deposited to the ProteomeXchange Consortium via the PRIDE partner repository with the dataset identifier PXD039137 (*S. boulardii*) and PXD039138 (*S. salivarius*).
