# Supplementary material for: Isolation and Characteristics of Extracellular Vesicles Produced by Probiotics: Yeast Saccharomyces boulardii CNCM I-745 and Bacterium Streptococcus salivarius K12
Source: Probiotics Antimicrob Proteins. 2023 May 20;16(3):936–48. doi: 10.1007/s12602-023-10085-3 (PMC11126510; doi:10.1007/s12602-023-10085-3)
Supplement: Supplementary file 1 — Supplementary file1 (PDF 2390 kb) [file 12602_2023_10085_MOESM1_ESM.pdf]

Isolation and characteristics of extracellular vesicles produced by probiotics: yeast *Saccharomyces boulardii* CNCM I-745 and bacterium *Streptococcus salivarius* K12

Kamila Kulig[1], Katarzyna Kowalik[1], Magdalena Surowiec[1,2], Elzbieta Karnas[3], Olga Barczyk-Woznicka[4], Ewa Zuba-Surma[3], Elzbieta Pyza[4], Andrzej Kozik[5], Maria Rapala-Kozik[1], Justyna Karkowska-Kuleta[1*]

[1] Department of Comparative Biochemistry and Bioanalytics, Faculty of Biochemistry, Biophysics and Biotechnology, Jagiellonian University, Kraków, Poland

[2] Doctoral School of Exact and Natural Sciences, Jagiellonian University, Kraków, Poland

[3] Department of Cell Biology, Faculty of Biochemistry, Biophysics and Biotechnology, Jagiellonian University, Kraków, Poland

[4] Department of Cell Biology and Imaging, Institute of Zoology and Biomedical Research, Jagiellonian University, Kraków, Poland

[5] Department of Analytical Biochemistry, Faculty of Biochemistry, Biophysics and Biotechnology, Jagiellonian University, Kraków, Poland

[*]Corresponding author: justyna.karkowska@uj.edu.pl

Supplementary Table 1. Mass spectrometry identification of *Saccharomyces boulardii* proteins in EVs. The proteomic analysis of extracellular vesicles was performed with samples prepared with a total amount of proteins equal to 11 µg. After digestion with Trypsin/Lys-C Mix the resulting peptides were analyzed using UltiMate 3000 RSLCnano System coupled with Q-Exactive mass spectrometer with DPV-550 Digital PicoView nanospray source. The obtained lists of peaks were searched against the NCBI protein database with the locally installed MASCOT search engine using the taxonomy restrictions: *Saccharomyces boulardii*. The Target Decoy PSM Validator was applied with the maximum false discovery rate (FDR) for peptides set to 0.01. Proteins identified with a score > 19 and a number of identified peptides ≥ 1 are only listed.

Accession number	Description	Molecular mass [kDa]	Score	Coverage	# Proteins	# Unique Peptides	# Peptides	# PSMs	# AAs	calc. pI
KQC43708.1	Bifunctional alcohol dehydrogenase and formaldehyde dehydrogenase [*Saccharomyces boulardii*]	35.7	11091.31	62.65	1	4	22	309	332	6.74
KQC42965.1	Glyceraldehyde-3-phosphate dehydrogenase (GAPDH), isozyme 2 [*Saccharomyces boulardii*]	35.8	10400.92	68.98	1	4	22	309	332	6.96
KQC40410.1	Alpha subunit of fatty acid synthetase [*Saccharomyces boulardii*]	206.9	8413.64	45.10	1	68	68	216	1887	5.50
KQC43016.1	Glyceraldehyde-3-phosphate dehydrogenase (GAPDH), isozyme 1 [*Saccharomyces boulardii*]	35.7	7428.09	68.67	1	1	21	210	332	8.51
KOH49774.1	TDH1p Glyceraldehyde-3-phosphate dehydrogenase (GAPDH), isozyme 1 [*Saccharomyces boulardii*]	35.7	7397.10	66.57	1	1	20	208	332	8.51
KQC42790.1	Beta subunit of fatty acid synthetase [*Saccharomyces boulardii*]	228.5	7050.62	38.81	1	63	63	184	2051	5.96
KQC45527.1	Translational elongation factor EF-1 alpha [*Saccharomyces boulardii*]	50.0	7022.61	41.70	1	15	16	223	458	9.04
KQC41085.1	Ribosomal 60S subunit protein L3 [*Saccharomyces boulardii*]	43.7	5335.31	38.50	1	14	14	136	387	10.29
KQC42168.1	PDC1p [*Saccharomyces boulardii*]	61.5	4491.80	43.16	2	17	20	98	563	6.19
KQC45164.1	Shmoo tip protein, substrate of Hub1p ubiquitin-like protein [*Saccharomyces boulardii*]	254.2	4134.69	28.26	2	40	40	78	2293	6.67
AGZ13685.1	pyruvate kinase [*Saccharomyces boulardii*]	54.5	4053.91	61.00	1	22	23	117	500	7.68
KQC41338.1	Acetyl-CoA carboxylase, biotin containing enzyme [*Saccharomyces boulardii*]	250.2	3978.62	31.21	2	54	54	129	2233	6.28
KQC43665.1	Alpha subunit of heterooctameric phosphofructokinase [*Saccharomyces boulardii*]	107.9	3737.42	41.95	1	30	31	72	987	6.39
KQC42352.1	Tetradecameric mitochondrial chaperonin [*Saccharomyces boulardii*]	60.7	3703.25	52.62	1	24	24	77	572	5.31
KQC44614.1	Elongation factor 2 (EF-2), also encoded by EFT1 [*Saccharomyces boulardii*]	93.2	3701.22	45.13	2	34	34	84	842	6.32
KQC42344.1	Translation elongation factor 3 [*Saccharomyces boulardii*]	115.9	3524.79	29.89	2	19	27	97	1044	6.05
KQC43088.1	Bifunctional carbamoylphosphate synthetase/aspartate transcarbamylase [*Saccharomyces boulardii*]	244.9	3197.91	29.45	3	55	56	89	2214	5.81
KQC41533.1	Cytoplasmic ATPase that is a ribosome-associated molecular chaperone [*Saccharomyces boulardii*]	66.6	3083.71	48.12	1	1	20	65	613	5.44
KOH50682.1	PMA1p Plasma membrane P2-type H+-ATPase [*Saccharomyces boulardii*]	99.6	3077.84	34.10	2	9	22	77	918	5.11
KQC41701.1	Cortical ER protein involved in ER-plasma membrane tethering [*Saccharomyces boulardii*]	196.4	3065.27	14.46	3	15	22	90	1770	8.63
KQC40590.1	Plasma membrane H+-ATPase [*Saccharomyces boulardii*]	102.1	3050.84	31.05	1	8	21	71	947	5.06
KQC40502.1	Ribosomal 60S subunit protein L5 [*Saccharomyces boulardii*]	33.7	2961.79	51.18	2	12	12	85	297	6.83
KQC45169.1	Cytoplasmic ATPase that is a ribosome-associated molecular chaperone [*Saccharomyces boulardii*]	66.6	2863.35	48.12	1	1	20	62	613	5.44
KQC45754.1	ATPase involved in protein folding and NLS-directed nuclear transport [*Saccharomyces boulardii*]	69.6	2744.17	50.16	1	8	22	70	642	5.11
KQC42108.1	hypothetical protein AB282_03288 [*Saccharomyces boulardii*]	69.5	2719.09	39.44	1	4	19	67	639	5.08

KQC43516.1	Peptidyl-prolyl cis-trans isomerase (cyclophilin) [*Saccharomyces boulardii*]	22.8	2711.42	50.73	1	8	8	53	205	6.13
KQC42685.1	Fructose 1,6-bisphosphate aldolase [*Saccharomyces boulardii*]	39.6	2636.56	42.06	1	11	11	59	359	5.78
KQC43404.1	Enolase II, a phosphopyruvate hydratase [*Saccharomyces boulardii*]	46.9	2601.09	47.83	1	8	18	60	437	6.00
KQC41711.1	Ribosomal protein 10 (rp10) of the small (40S) subunit [*Saccharomyces boulardii*]	28.8	2576.28	51.37	1	2	12	89	255	10.02
KQC42509.1	Ribosomal protein 10 (rp10) of the small (40S) subunit [*Saccharomyces boulardii*]	28.7	2561.01	54.51	1	3	13	90	255	9.99
KQC45706.1	Acetyl-coA synthetase isoform [*Saccharomyces boulardii*]	79.1	2546.64	39.69	1	22	23	66	713	6.62
KQC45448.1	Ribosomal 60S subunit protein L4A [*Saccharomyces boulardii*]	39.1	2534.13	44.75	1	12	12	57	362	10.64
KQC43803.1	Eisosome core component [*Saccharomyces boulardii*]	38.3	2495.60	52.51	1	11	13	66	339	4.63
KQC42998.1	ATPase involved in protein import into the ER [*Saccharomyces boulardii*]	74.4	2493.48	41.20	1	21	22	62	682	4.93
KQC42515.1	Ribosomal 60S subunit protein L6B [*Saccharomyces boulardii*]	20.0	2323.48	51.70	1	3	10	78	176	10.08
KQC45484.1	Small heat shock protein (sHSP) with chaperone activity [*Saccharomyces boulardii*]	23.9	2279.98	55.61	1	12	12	63	214	5.53
KQC43357.1	Invertase [*Saccharomyces boulardii*]	58.6	2277.61	29.10	1	13	13	58	512	4.73
KQC44194.1	Actin [*Saccharomyces boulardii*]	41.7	2177.71	47.20	2	14	14	56	375	5.68
KQC45076.1	Component of the Mcm2-7 hexameric helicase complex [*Saccharomyces boulardii*]	92.0	2174.09	40.72	1	28	28	60	835	4.94
KQC41936.1	Cytoplasmic chaperone of the Hsp90 family [*Saccharomyces boulardii*]	80.8	2102.00	37.87	2	5	26	58	705	4.83
AGZ13686.1	phosphopyruvate hydratase enolase i [*Saccharomyces boulardii*]	46.8	2095.63	44.62	1	7	17	46	437	6.62
KQC42421.1	FK506 Sensitivity [*Saccharomyces boulardii*]	214.9	2075.72	19.83	2	17	26	45	1876	7.11
KQC42090.1	Ribosomal 60S subunit protein L8B [*Saccharomyces boulardii*]	28.1	2051.29	50.00	3	2	14	53	256	10.02
KQC41678.1	Large subunit of trehalose 6-phosphate synthase/phosphatase complex [*Saccharomyces boulardii*]	122.9	1982.96	24.95	1	22	23	51	1098	6.70
KQC40810.1	Mitochondrial aldehyde dehydrogenase [*Saccharomyces boulardii*]	56.7	1975.13	56.07	1	20	20	53	519	6.74
KQC41955.1	Beta subunit of heterooctameric phosphofructokinase [*Saccharomyces boulardii*]	104.5	1970.81	33.06	1	23	24	46	959	6.52
KQC42114.1	Mitochondrial aspartyl-tRNA synthetase [*Saccharomyces boulardii*]	63.5	1935.08	44.52	2	22	22	43	557	6.58
KQC40402.1	Hsp90 chaperone [*Saccharomyces boulardii*]	81.3	1868.09	37.38	2	4	25	53	709	4.91
KQC45443.1	P-loop ATPase [*Saccharomyces boulardii*]	44.1	1864.36	37.06	1	13	13	44	394	7.43
KQC40782.1	Subunit 2 of ubiquinol cytochrome-c reductase (Complex III) [*Saccharomyces boulardii*]	40.4	1858.02	58.42	1	17	17	65	368	7.96
AGZ13684.1	phosphoglycerate kinase [*Saccharomyces boulardii*]	44.7	1853.79	49.04	1	18	18	50	416	7.61
KQC43395.1	6-phosphogluconate dehydrogenase (decarboxylating) [*Saccharomyces boulardii*]	53.5	1838.94	34.15	1	11	14	42	489	6.64
KQC40526.1	ATPase component of heat shock protein Hsp90 chaperone complex [*Saccharomyces boulardii*]	77.3	1779.72	28.72	5	14	17	47	693	5.22
KQC43968.1	ARC1p that binds tRNA and methionyl- and glutamyl-tRNA synthetase [*Saccharomyces boulardii*]	42.1	1745.54	36.70	1	11	11	58	376	7.88
KQC43478.1	Hexose transporter with moderate affinity for glucose [*Saccharomyces boulardii*]	66.2	1714.05	24.49	5	10	14	37	592	7.61

KQC41353.1	Citrate synthase [*Saccharomyces boulardii*]	53.3	1699.10	31.94	1	14	14	42	479	8.29
KQC42866.1	Beta subunit of the F1 sector of mitochondrial F1F0 ATP synthase [*Saccharomyces boulardii*]	54.8	1658.55	39.73	1	13	13	42	511	5.71
KQC45617.1	Pyruvate carboxylase isoform [*Saccharomyces boulardii*]	130.1	1612.39	23.90	1	5	24	47	1180	6.51
KQC43108.1	Inositol-3-phosphate synthase [*Saccharomyces boulardii*]	59.6	1578.60	27.39	1	13	13	35	533	5.92
KQC42106.1	Disaggregase [*Saccharomyces boulardii*]	102.0	1554.39	35.13	1	29	30	46	908	5.45
KQC45263.1	Acetate transporter required for normal sporulation [*Saccharomyces boulardii*]	30.7	1525.78	13.07	1	2	2	26	283	5.15
KQC44347.1	ATPase of the ATP-binding cassette (ABC) family [*Saccharomyces boulardii*]	68.3	1522.76	36.07	1	19	19	28	610	6.51
KQC43605.1	Ribosomal 60S subunit protein L8A [*Saccharomyces boulardii*]	28.1	1520.81	54.30	3	3	15	41	256	10.04
KQC44379.1	Isocitrate lyase [*Saccharomyces boulardii*]	62.4	1519.38	32.85	1	14	14	37	557	6.42
KQC41147.1	Phospholipase B (lysophospholipase) involved in lipid metabolism [*Saccharomyces boulardii*]	75.0	1498.44	24.05	1	11	12	33	686	5.03
KQC41652.1	hypothetical protein AB282_03715 [*Saccharomyces boulardii*]	59.6	1476.90	38.21	1	18	18	36	513	7.97
KQC44654.1	High-affinity glucose transporter [*Saccharomyces boulardii*]	62.7	1456.45	23.86	9	6	10	37	570	7.75
KQC43375.1	RPS4Ap component of the small (40S) ribosomal subunit [*Saccharomyces boulardii*]	29.4	1455.36	47.51	3	14	14	42	261	10.08
KQC45535.1	Subunit B of V1 peripheral membrane domain of vacuolar H+-ATPase [*Saccharomyces boulardii*]	57.7	1449.93	44.29	1	18	18	35	517	5.07
KQC41508.1	RPS3p component of the small (40S) ribosomal subunit [*Saccharomyces boulardii*]	26.5	1395.20	68.75	1	16	16	36	240	9.41
KQC40619.1	Eisosome core component [*Saccharomyces boulardii*]	38.0	1392.26	43.99	1	11	12	30	341	4.70
KQC41366.1	Translational elongation factor EF-3 [*Saccharomyces boulardii*]	115.8	1377.32	17.05	2	7	15	38	1044	6.18
KQC44055.1	Clathrin heavy chain [*Saccharomyces boulardii*]	187.0	1364.90	18.15	2	26	26	36	1653	5.27
KQC41403.1	Mitochondrial NADH kinase [*Saccharomyces boulardii*]	30.4	1360.10	56.54	1	13	13	31	283	8.32
KQC42537.1	Phosphoenolpyruvate carboxykinase [*Saccharomyces boulardii*]	60.9	1357.41	33.33	1	14	14	33	549	6.34
AML33326.1	aldehyde dehydrogenase Ald6p [*Saccharomyces boulardii*]	54.4	1354.23	54.00	2	19	19	41	500	5.44
KQC43932.1	Pyruvate carboxylase isoform [*Saccharomyces boulardii*]	129.9	1347.18	23.34	1	4	23	40	1178	6.21
KQC43849.1	Catalytic subunit of 1,3-beta-glucan synthase [*Saccharomyces boulardii*]	216.9	1340.89	15.04	2	9	19	31	1895	6.98
KOH52313.1	RPG1p eIF3a subunit of the eukaryotic translation initiation factor 3 (eIF3) [*Saccharomyces boulardii*]	110.5	1339.27	27.07	2	25	25	42	964	6.38
KQC42050.1	Cell wall protein with similarity to glucanase [*Saccharomyces boulardii*]	40.4	1329.62	25.96	1	9	9	24	389	4.65
KQC41288.1	Daughter cell-specific secreted protein with similarity to glucanase [*Saccharomyces boulardii*]	121.0	1319.11	14.68	1	9	9	22	1117	4.53
KQC44127.1	Ribosomal 60S subunit protein L2B [*Saccharomyces boulardii*]	27.4	1305.90	49.61	2	10	10	42	254	11.11
KQC42864.1	RPS5p component of the small (40S) ribosomal subunit [*Saccharomyces boulardii*]	25.0	1278.12	32.44	1	7	7	27	225	8.59
KQC44827.1	Dihydrolipoyl transsuccinylase [*Saccharomyces boulardii*]	50.4	1263.35	41.04	1	13	13	37	463	8.85
KQC42725.1	Glutamine-fructose-6-phosphate amidotransferase [*Saccharomyces boulardii*]	80.0	1258.34	33.05	1	19	19	31	717	6.43
KQC42937.1	Hsp70 family ATPase [*Saccharomyces boulardii*]	70.8	1238.56	39.85	2	20	20	32	655	5.53

KQC43284.1	Threonyl-tRNA synthetase [*Saccharomyces boulardii*]	84.4	1237.19	37.19	1	25	25	35	734	6.93
KQC45352.1	Cytoplasmic isoleucine-tRNA synthetase [*Saccharomyces boulardii*]	122.8	1229.05	24.07	1	20	20	31	1072	6.11
KQC45353.1	ATPase involved in protein folding and the response to stress [*Saccharomyces boulardii*]	70.5	1223.12	26.50	1	10	13	29	649	5.17
KQC45082.1	Homocitrate synthase isozyme [*Saccharomyces boulardii*]	48.6	1218.53	30.68	1	1	10	29	440	6.30
KQC41895.1	Ribosomal 60S subunit protein L13B [*Saccharomyces boulardii*]	22.5	1217.14	47.74	2	10	10	27	199	11.08
KQC45300.1	Member of the protein disulfide isomerase (PDI) family [*Saccharomyces boulardii*]	58.2	1211.27	29.50	1	2	10	22	522	4.53
KQC45343.1	Ribosomal 60S subunit protein L23B [*Saccharomyces boulardii*]	14.5	1197.14	47.45	1	6	6	30	137	10.33
KQC41038.1	ATPase of the 19S regulatory particle of the 26S proteasome [*Saccharomyces boulardii*]	48.2	1183.19	49.08	1	17	17	30	434	5.06
KQC45096.1	Alpha subunit of COPI vesicle coatomer complex [*Saccharomyces boulardii*]	135.5	1157.72	21.65	1	19	19	33	1201	6.04
KQC44892.1	Phosphatase subunit of the trehalose-6-P synthase/phosphatase complex [*Saccharomyces boulardii*]	102.9	1153.51	25.56	2	18	18	30	896	8.05
KQC42629.1	Dynamin-like GTPase required for vacuolar sorting [*Saccharomyces boulardii*]	78.7	1138.44	26.99	2	17	18	27	704	7.91
KQC41777.1	Subunit of the eukaryotic translation initiation factor 3 (eIF3) [*Saccharomyces boulardii*]	145.0	1133.54	20.60	1	22	22	32	1277	6.39
KQC42621.1	3-hydroxyacyl-CoA dehydrogenase and enoyl-CoA hydratase [*Saccharomyces boulardii*]	98.6	1126.09	29.78	1	22	22	30	900	9.00
KQC45332.1	Alpha subunit of the F1 sector of mitochondrial F1F0 ATP synthase [*Saccharomyces boulardii*]	58.6	1115.65	32.29	1	16	16	33	545	9.04
KQC42705.1	Gamma subunit of translational elongation factor eEF1B [*Saccharomyces boulardii*]	46.5	1110.36	32.04	1	10	12	29	412	7.87
KQC42302.1	Essential evolutionarily-conserved nucleolar protein [*Saccharomyces boulardii*]	56.8	1108.30	35.71	1	14	14	27	504	8.90
KQC40480.1	Vacuolar aspartyl protease (proteinase A) [*Saccharomyces boulardii*]	44.5	1102.45	28.64	1	8	8	25	405	4.84
KQC43942.1	Ribosomal 60S subunit protein L7A [*Saccharomyces boulardii*]	27.6	1093.58	48.77	1	13	13	29	244	10.15
KQC45390.1	Major ADP/ATP carrier of the mitochondrial inner membrane [*Saccharomyces boulardii*]	34.4	1084.95	35.53	2	11	12	40	318	9.77
KQC42281.1	Cytosolic NADP-specific isocitrate dehydrogenase [*Saccharomyces boulardii*]	46.5	1084.07	40.78	1	12	14	29	412	6.19
KQC42271.1	Acetyl-coA synthetase isoform [*Saccharomyces boulardii*]	75.4	1075.66	31.19	1	17	18	27	683	6.71
KQC44088.1	Glutamyl-tRNA synthetase (GluRS) [*Saccharomyces boulardii*]	80.8	1071.82	28.53	1	22	22	29	708	7.53
KOH51991.1	PDI1p Protein disulfide isomerase [*Saccharomyces boulardii*]	56.8	1070.82	25.93	1	1	9	19	509	4.63
KQC41457.1	Malate synthase, enzyme of the glyoxylate cycle [*Saccharomyces boulardii*]	62.7	1063.48	26.17	2	13	13	32	554	7.03
KQC45432.1	Alpha-1,2-mannosyltransferase [*Saccharomyces boulardii*]	67.7	1063.22	38.53	1	2	17	37	597	6.06
KQC41637.1	Glycosylphosphatidylinositol (GPI)-anchored cell wall endoglucanase [*Saccharomyces boulardii*]	108.3	1053.85	10.18	1	5	5	19	1041	4.81
KOH52370.1	MNN2p Alpha-1,2-mannosyltransferase [*Saccharomyces boulardii*]	67.7	1043.20	37.86	1	1	16	36	597	6.27
KQC40574.1	mannosylphosphate transferase [*Saccharomyces boulardii*]	48.2	1034.47	39.81	1	12	12	21	412	5.08
KQC41173.1	Ribosomal protein 51 (rp51) of the small (40s) subunit [*Saccharomyces boulardii*]	16.0	1033.85	33.80	5	4	4	28	142	10.70

KQC44336.1	hypothetical protein AB282_01592 [*Saccharomyces boulardii*]	157.3	1032.22	18.14	3	17	18	27	1417	6.21
KQC42788.1	Ribosomal 60S subunit protein L17A [*Saccharomyces boulardii*]	20.5	1027.25	39.13	1	1	6	21	184	10.92
KQC44406.1	Cobalamin-independent methionine synthase [*Saccharomyces boulardii*]	85.8	1015.94	31.16	1	21	21	28	767	6.47
KQC45034.1	RPS16Bp component of the small (40S) ribosomal subunit [*Saccharomyces boulardii*]	15.8	1010.12	45.45	1	6	6	23	143	10.26
AGZ13687.1	alcohol dehydrogenase [*Saccharomyces boulardii*]	32.9	1002.53	45.31	3	11	14	30	309	7.12
KQC42724.1	hypothetical protein AB282_03152 [*Saccharomyces boulardii*]	57.0	1001.97	27.82	1	9	9	20	514	5.74
KQC42500.1	Inosine monophosphate dehydrogenase [*Saccharomyces boulardii*]	56.5	999.78	30.02	2	6	10	22	523	7.40
KQC40753.1	Glycogen phosphorylase required for the mobilization of glycogen [*Saccharomyces boulardii*]	103.2	997.37	26.27	1	18	19	29	902	5.62
KQC43802.1	Minor isoform of pyruvate decarboxylase [*Saccharomyces boulardii*]	61.5	984.26	17.76	1	5	8	16	563	6.19
KQC44912.1	RPS13p component of the small (40S) ribosomal subunit [*Saccharomyces boulardii*]	17.0	980.64	56.29	1	8	8	32	151	10.43
KQC40902.1	Subunit a of vacuolar-ATPase V0 domain [*Saccharomyces boulardii*]	95.5	976.42	20.71	1	14	14	25	840	5.48
KQC41056.1	RPS7Ap component of the small (40S) ribosomal subunit [*Saccharomyces boulardii*]	21.6	975.23	35.26	1	4	7	32	190	9.83
KQC40839.1	RNA polymerase I largest subunit A190 [*Saccharomyces boulardii*]	186.3	970.01	12.74	1	14	15	19	1664	7.08
KQC44107.1	Hexokinase isoenzyme 1 [*Saccharomyces boulardii*]	53.7	969.35	47.84	1	15	15	24	485	5.45
KQC43630.1	Cell wall protein with similarity to glucanase [*Saccharomyces boulardii*]	40.1	960.18	10.62	1	3	3	13	386	4.79
KQC45128.1	Homocitrate synthase isozyme [*Saccharomyces boulardii*]	47.1	958.56	33.88	1	2	11	20	428	7.27
KQC45299.1	Glucokinase [*Saccharomyces boulardii*]	55.4	956.43	28.80	1	11	11	26	500	6.25
KQC45583.1	RPS6Bp component of the small (40S) ribosomal subunit [*Saccharomyces boulardii*]	27.0	941.14	39.83	2	10	11	24	236	10.45
KQC43717.1	Ribonucleotide-diphosphate reductase (RNR) small subunit [*Saccharomyces boulardii*]	40.0	936.15	25.51	1	8	8	16	345	5.21
KOH49880.1	MIR1p Mitochondrial phosphate carrier [*Saccharomyces boulardii*]	32.8	935.28	38.26	2	10	10	21	311	9.31
KQC43350.1	Mitochondrial glycerol-3-phosphate dehydrogenase [*Saccharomyces boulardii*]	72.3	917.98	37.44	1	22	22	30	649	7.71
KQC40474.1	Cytosolic leucyl tRNA synthetase [*Saccharomyces boulardii*]	124.1	915.69	21.83	1	17	17	19	1090	5.90
KQC45130.1	Subunit A of the V1 peripheral membrane domain of V-ATPase [*Saccharomyces boulardii*]	118.5	914.53	19.33	1	17	17	24	1071	6.10
KQC43332.1	Mitochondrial outer membrane protein [*Saccharomyces boulardii*]	44.6	914.21	38.68	1	15	15	25	393	8.75
KQC44046.1	Positive regulator of the Gcn2p kinase activity [*Saccharomyces boulardii*]	296.5	906.01	9.09	1	21	21	23	2672	5.26
KQC44985.1	NAD-dependent glycerol-3-phosphate dehydrogenase [*Saccharomyces boulardii*]	42.8	900.19	26.34	1	7	7	16	391	5.47
KQC41253.1	Ribosomal 60S subunit protein L25 [*Saccharomyces boulardii*]	15.7	889.50	42.25	1	5	5	21	142	10.11
KQC43251.1	BAR domain protein [*Saccharomyces boulardii*]	36.6	883.60	28.83	1	8	8	15	326	4.97
KQC43130.1	Ribosomal 60S subunit protein L17B [*Saccharomyces boulardii*]	20.5	883.24	39.13	1	1	6	18	184	10.92
KQC42444.1	RPS22Bp component of the small (40S) ribosomal subunit [*Saccharomyces boulardii*]	14.6	860.59	48.46	2	6	6	26	130	9.94
KQC40990.1	Glutamine tRNA synthetase [*Saccharomyces boulardii*]	93.1	860.34	21.76	1	16	16	22	809	8.41

KQC44013.1	Vacuolar alpha mannosidase [*Saccharomyces boulardii*]	124.4	853.01	22.25	7	19	20	31	1083	7.24
KQC40960.1	ATP-dependent DEAD (Asp-Glu-Ala-Asp)-box RNA helicase [*Saccharomyces boulardii*]	65.5	852.10	26.99	1	10	14	25	604	7.83
KOH49748.1	SCP160p RNA-binding G protein effector of mating response pathway [*Saccharomyces boulardii*]	134.7	847.62	21.69	2	21	21	26	1222	5.85
KQC44537.1	Ribosomal 60S subunit protein L27A [*Saccharomyces boulardii*]	15.2	843.69	53.38	2	8	8	23	133	10.35
KQC40863.1	Ribosomal 60S subunit protein L20B [*Saccharomyces boulardii*]	20.7	835.11	45.40	2	9	9	21	174	10.26
KQC45530.1	Plasma membrane protein [*Saccharomyces boulardii*]	75.3	820.98	34.03	1	20	20	26	667	5.81
KQC43795.1	Mitochondrial and cytoplasmic valyl-tRNA synthetase [*Saccharomyces boulardii*]	120.1	813.11	21.36	1	20	20	26	1058	6.33
KQC42182.1	Cytosolic serine hydroxymethyltransferase [*Saccharomyces boulardii*]	52.2	809.79	33.90	1	14	15	27	469	7.43
KQC43627.1	Endo-beta-1,3-glucanase [*Saccharomyces boulardii*]	34.1	801.30	23.32	1	6	6	17	313	4.51
KQC45597.1	Glycolytic enzyme phosphoglucose isomerase [*Saccharomyces boulardii*]	61.3	787.14	19.13	1	7	7	25	554	6.46
KQC44232.1	Poly(A) binding protein [*Saccharomyces boulardii*]	64.3	787.13	25.82	1	11	11	18	577	5.97
KQC44054.1	Fatty-acyl coenzyme A oxidase [*Saccharomyces boulardii*]	83.9	786.94	21.12	1	13	13	20	748	8.38
KQC44957.1	Plasma membrane ATP-binding cassette (ABC) transporter [*Saccharomyces boulardii*]	168.6	778.35	15.99	3	17	17	18	1501	7.69
KOH51464.1	NOP1p Histone glutamine methyltransferase, modifies H2A at Q105 in nucleolus [*Saccharomyces boulardii*]	32.9	776.08	27.51	4	9	9	25	309	9.99
KQC42345.1	hypothetical protein AB282_03527 [*Saccharomyces boulardii*]	27.3	773.44	32.91	1	6	6	22	234	5.11
KQC45600.1	putative mannosyltransferase involved in protein glycosylation [*Saccharomyces boulardii*]	54.5	768.18	38.36	2	12	12	16	464	4.83
KQC44388.1	RPS24Ap component of the small (40S) ribosomal subunit [*Saccharomyces boulardii*]	15.3	763.70	37.04	2	5	5	21	135	10.51
KQC43994.1	Ribosomal 60S subunit protein L1A [*Saccharomyces boulardii*]	24.5	763.61	31.80	1	8	8	20	217	9.72
KQC45268.1	hypothetical protein AB282_00540 [*Saccharomyces boulardii*]	26.3	762.80	46.56	1	7	7	14	247	8.19
KQC43265.1	hypothetical protein AB282_01341 [*Saccharomyces boulardii*]	19.2	762.65	62.80	1	9	9	29	164	7.39
KQC42964.1	ATP sulfurylase [*Saccharomyces boulardii*]	57.7	758.96	14.87	1	6	6	21	511	5.82
KOH52307.1	RPL19Ap Ribosomal 60S subunit protein L19A [*Saccharomyces boulardii*]	21.5	755.99	32.09	4	6	6	16	187	11.12
KQC41387.1	Subunit of mitochondrial NAD(+)-dependent isocitrate dehydrogenase [*Saccharomyces boulardii*]	39.3	753.24	35.00	1	10	10	25	360	9.00
KQC45244.1	RPS14Ap component of the small (40S) ribosomal subunit [*Saccharomyces boulardii*]	14.5	745.71	61.31	2	7	7	22	137	10.73
KQC44220.1	14-3-3 protein, major isoform [*Saccharomyces boulardii*]	30.1	740.58	41.20	1	3	9	19	267	4.88
KQC43740.1	Cystathionine beta-synthase [*Saccharomyces boulardii*]	56.0	738.85	38.26	1	15	15	25	507	6.70
KQC45011.1	GDP-mannose pyrophosphorylase (mannose-1-phosphate guanyltransferase) [*Saccharomyces boulardii*]	39.6	733.22	47.65	1	5	13	22	361	6.34
KQC42759.1	ATPase of the 19S regulatory particle of the 26S proteasome [*Saccharomyces boulardii*]	52.0	729.72	37.26	1	14	15	31	467	5.47
KQC44933.1	Lysyl-tRNA synthetase [*Saccharomyces boulardii*]	67.9	722.61	27.24	1	14	14	20	591	6.13
KQC45026.1	Ribosomal 60S subunit protein L31B [*Saccharomyces boulardii*]	12.9	721.79	31.86	1	1	3	25	113	9.99

KQC45850.1	RPS4Ap component of the small (40S) ribosomal subunit [*Saccharomyces boulardii*]	48.8	719.83	24.03	1	7	7	13	437	6.04
KQC43922.1	ATPase of the 19S regulatory particle of the 26S proteasome [*Saccharomyces boulardii*]	45.2	714.22	31.36	1	10	11	21	405	9.01
KQC45376.1	Core subunit of the ubiquinol-cytochrome c reductase complex [*Saccharomyces boulardii*]	50.2	711.31	33.04	1	13	13	19	457	7.30
KQC44701.1	Translational repressor with a role in polar growth and wall integrity [*Saccharomyces boulardii*]	139.9	708.72	19.12	1	14	15	17	1250	7.64
KQC44056.1	Subunit of the heterodimeric FACT complex (Spt16p-Pob3p) [*Saccharomyces boulardii*]	118.6	708.33	17.78	1	15	16	20	1035	5.12
KQC43546.1	Non-ATPase base subunit of the 19S RP of the 26S proteasome [*Saccharomyces boulardii*]	109.5	705.34	15.91	1	13	13	19	993	4.63
KQC43683.1	Major karyopherin [*Saccharomyces boulardii*]	124.1	701.38	19.19	1	18	18	21	1084	5.48
KQC42388.1	Aconitase [*Saccharomyces boulardii*]	85.3	701.22	27.76	9	16	17	21	778	7.93
KQC44868.1	14-3-3 protein, minor isoform [*Saccharomyces boulardii*]	31.0	700.44	35.53	1	2	8	18	273	4.88
KQC42156.1	Ribosomal 60S subunit protein L15A [*Saccharomyces boulardii*]	24.4	698.96	43.63	1	2	10	23	204	11.39
KQC43643.1	Methionyl-tRNA synthetase [*Saccharomyces boulardii*]	85.6	698.91	23.17	1	14	15	18	751	6.61
KQC42663.1	UDP-glucose pyrophosphorylase (UGPase) [*Saccharomyces boulardii*]	56.2	698.72	31.34	1	14	14	20	501	7.44
KQC44417.1	RPS8Bp component of the small (40S) ribosomal subunit [*Saccharomyces boulardii*]	22.5	698.51	43.00	2	8	8	17	200	10.74
KQC44193.1	Rab family GTPase [*Saccharomyces boulardii*]	23.2	696.87	50.00	1	7	9	12	206	5.33
KQC43687.1	Ribosomal 40S subunit protein S0A [*Saccharomyces boulardii*]	28.0	687.54	56.35	1	2	8	20	252	4.72
KQC45035.1	Component of the TREX complex required for nuclear mRNA export [*Saccharomyces boulardii*]	50.2	682.43	24.22	1	10	10	15	446	5.52
KOH51201.1	GLC3p Glycogen branching enzyme, involved in glycogen accumulation [*Saccharomyces boulardii*]	81.1	679.18	15.48	2	10	10	19	704	6.28
KQC44029.1	Evolutionarily-conserved 5'-3' exonuclease [*Saccharomyces boulardii*]	175.4	673.69	13.02	1	17	17	20	1528	7.55
KQC44326.1	Medium chain fatty acyl-CoA synthetase [*Saccharomyces boulardii*]	83.4	672.83	19.49	1	11	11	14	744	7.74
KQC41875.1	Ribosomal 60S subunit protein L15B [*Saccharomyces boulardii*]	24.4	671.01	38.73	1	1	9	21	204	11.39
KQC43509.1	Hsp70 protein that interacts with Zuo1p [*Saccharomyces boulardii*]	58.2	669.03	30.67	1	14	14	16	538	5.05
KQC43671.1	Nitric oxide oxidoreductase [*Saccharomyces boulardii*]	44.6	666.53	19.55	1	6	6	19	399	6.28
KQC41247.1	RPS19Ap component of the small (40S) ribosomal subunit [*Saccharomyces boulardii*]	15.9	665.04	46.53	2	6	6	14	144	9.61
KQC42088.1	Transporter of the ATP-binding cassette (ABC) family [*Saccharomyces boulardii*]	189.0	661.28	9.75	1	13	13	16	1661	8.09
KQC45430.1	Cytoplasmic inorganic pyrophosphatase (PPase) [*Saccharomyces boulardii*]	32.3	656.38	52.61	1	10	11	17	287	5.58
KQC44282.1	Translation elongation factor eIF-5A [*Saccharomyces boulardii*]	17.1	653.28	29.30	2	4	4	13	157	4.96
KQC40937.1	Transcriptional modulator [*Saccharomyces boulardii*]	48.4	647.14	25.63	1	8	8	13	437	5.36
KQC40963.1	Component of mRNP complex [*Saccharomyces boulardii*]	54.6	645.32	28.30	1	12	12	17	470	9.19
KQC45147.1	Reticulon protein [*Saccharomyces boulardii*]	44.3	644.96	30.53	1	10	10	16	393	9.22
KQC41979.1	RNA binding protein [*Saccharomyces boulardii*]	193.0	643.65	12.38	1	19	20	23	1729	6.21
KQC43321.1	hypothetical protein AB282_01398 [*Saccharomyces boulardii*]	114.3	639.38	17.75	1	14	14	22	1014	7.14

KQC45139.1	Component of the Sec13p-Sec31p complex of the COPII vesicle coat [*Saccharomyces boulardii*]	138.7	638.87	10.13	1	8	8	11	1273	5.72
KQC42129.1	Dynamin-related GTPase involved in mitochondrial organization [*Saccharomyces boulardii*]	84.9	637.20	23.78	2	12	13	14	757	5.52
KQC41007.1	RNA polymerase II second largest subunit B150 [*Saccharomyces boulardii*]	138.7	633.66	10.87	1	9	9	17	1224	6.52
KQC42432.1	Transaldolase [*Saccharomyces boulardii*]	37.0	633.30	41.79	1	12	12	15	335	6.11
KQC40852.1	Type V myosin motor [*Saccharomyces boulardii*]	180.6	633.11	11.69	2	14	14	16	1574	6.32
KOH51885.1	RPS17Bp Ribosomal protein 51 (rp51) of the small (40s) subunit [*Saccharomyces boulardii*]	15.7	631.61	51.85	2	1	7	16	135	10.43
KQC44945.1	Cytosolic seryl-tRNA synthetase [*Saccharomyces boulardii*]	53.3	629.93	28.57	1	10	10	16	462	6.09
KQC42048.1	Glucose-repressible alcohol dehydrogenase II [*Saccharomyces boulardii*]	36.7	627.59	27.30	2	6	9	16	348	6.74
KQC42707.1	Mitochondrial malate dehydrogenase [*Saccharomyces boulardii*]	35.6	626.71	35.03	1	8	8	14	334	8.47
KQC40845.1	Subunit of the APT subcomplex of cleavage and polyadenylation factor [*Saccharomyces boulardii*]	107.2	623.75	24.11	1	19	19	20	958	5.60
KQC42198.1	Ribosomal 60S subunit protein L10 [*Saccharomyces boulardii*]	25.3	623.09	19.91	1	5	5	29	221	10.02
KQC41760.1	Component of the universally conserved Spt4/5 complex (DSIF complex) [*Saccharomyces boulardii*]	114.4	620.65	13.96	1	10	10	13	1053	5.39
KQC40881.1	RPS10Ap component of the small (40S) ribosomal subunit [*Saccharomyces boulardii*]	12.7	620.64	57.14	2	4	4	20	105	8.79
KQC40911.1	ATPase of the 19S regulatory particle of the 26S proteasome [*Saccharomyces boulardii*]	49.4	616.37	22.65	1	10	10	21	437	5.73
KQC40859.1	Long chain fatty acyl-CoA synthetase [*Saccharomyces boulardii*]	77.8	615.51	23.43	1	13	13	18	700	7.62
KQC41360.1	Type II HSP40 co-chaperone that interacts with the HSP70 protein Ssa1p [*Saccharomyces boulardii*]	38.3	614.24	27.58	1	7	7	15	359	9.03
KQC42172.1	RPS0Bp component of the small (40S) ribosomal subunit [*Saccharomyces boulardii*]	27.9	613.95	56.35	1	2	8	19	252	4.75
KQC41761.1	Delta(24)-sterol C-methyltransferase [*Saccharomyces boulardii*]	43.4	613.48	34.99	1	11	11	16	383	5.77
KQC41811.1	Constituent of 66S pre-ribosomal particle [*Saccharomyces boulardii*]	91.6	607.27	18.84	1	11	11	18	807	5.16
KQC44585.1	Ribosomal 60S subunit protein L12A [*Saccharomyces boulardii*]	17.8	602.08	47.88	1	6	6	14	165	9.41
KQC43996.1	Essential beta'-coat protein of the COPI coatomer [*Saccharomyces boulardii*]	99.4	601.84	24.52	9	17	18	19	889	4.74
KQC41112.1	Hsp90 cochaperone [*Saccharomyces boulardii*]	66.3	599.42	25.98	1	11	11	14	589	5.59
KQC45403.1	Type I transmembrane sorting receptor for multiple vacuolar hydrolase [*Saccharomyces boulardii*]	177.4	597.03	10.46	3	15	15	19	1577	4.92
KQC42478.1	Ribosomal 60S subunit protein L31B [*Saccharomyces boulardii*]	13.0	595.85	31.86	1	1	3	19	113	9.99
KQC43801.1	Cytosolic catalase T [*Saccharomyces boulardii*]	64.5	595.80	27.58	1	10	10	16	562	6.44
KQC44177.1	Alpha subunit of cytoplasmic phenylalanyl-tRNA synthetase [*Saccharomyces boulardii*]	57.4	593.64	24.25	1	9	9	12	503	5.78
KQC44938.1	PST2p with similarity to a family of flavodoxin-like protein [*Saccharomyces boulardii*]	20.9	591.49	45.45	1	5	5	10	198	5.49
KQC44556.1	RPS18Bp component of the small (40S) ribosomal subunit [*Saccharomyces boulardii*]	17.0	583.54	43.84	1	7	7	15	146	10.27
KQC41658.1	NADH:ubiquinone oxidoreductase [*Saccharomyces boulardii*]	57.3	582.49	25.73	1	10	10	18	513	9.44

KOH48793.1	RPS17Ap Ribosomal protein 51 (rp51) of the small (40s) subunit [*Saccharomyces boulardii*]	16.2	580.10	50.72	2	1	7	15	138	10.33
KQC43697.1	Cytoplasmic trifunctional enzyme C1-tetrahydrofolate synthase [*Saccharomyces boulardii*]	102.0	579.08	17.34	3	15	15	17	946	6.76
KQC44410.1	Beta 3 subunit of the 20S proteasome [*Saccharomyces boulardii*]	22.6	578.79	14.15	1	2	2	8	205	5.15
KQC44728.1	Oligomeric mitochondrial matrix chaperone [*Saccharomyces boulardii*]	81.7	578.57	21.32	1	10	12	15	727	6.04
KQC45808.1	Outer mitochondrial carnitine acetyltransferase [*Saccharomyces boulardii*]	77.7	572.32	14.56	1	8	8	15	687	6.93
KQC43966.1	Ribosomal 60S subunit protein L28 [*Saccharomyces boulardii*]	16.7	572.25	34.23	1	5	5	22	149	10.62
KQC42287.1	S-adenosylmethionine synthetase [*Saccharomyces boulardii*]	41.8	567.08	26.96	1	3	8	12	382	5.22
KQC42485.1	Plasma membrane protein with a role in cell wall integrity [*Saccharomyces boulardii*]	24.1	565.67	14.81	1	2	2	23	216	8.73
KQC43650.1	6-phosphogluconate dehydrogenase (decarboxylating) [*Saccharomyces boulardii*]	53.9	563.77	32.93	1	11	14	20	492	7.30
KQC45620.1	Oxalyl-CoA synthetase [*Saccharomyces boulardii*]	60.4	563.69	20.63	3	10	10	15	543	9.20
KQC44746.1	Essential beta-coat protein of the COPI coatomer [*Saccharomyces boulardii*]	108.9	563.12	16.24	1	13	13	16	973	5.68
KQC44605.1	ATPase of the 19S regulatory particle of the 26S proteasome [*Saccharomyces boulardii*]	47.9	558.00	16.59	1	6	6	15	428	5.53
KQC41796.1	Component of the eisosome required for proper eisosome assembly [*Saccharomyces boulardii*]	93.3	556.58	14.71	1	11	11	14	843	6.27
KQC40392.1	Ribosomal 60S subunit protein L36B [*Saccharomyces boulardii*]	11.1	550.32	46.00	2	5	5	15	100	11.59
KQC41410.1	Type I HSP40 co-chaperone [*Saccharomyces boulardii*]	44.6	549.29	26.41	1	9	9	18	409	6.30
KQC44805.1	Translation termination factor eRF3 [*Saccharomyces boulardii*]	76.6	547.62	17.23	1	9	10	14	685	6.86
KQC44508.1	S-adenosylmethionine synthetase [*Saccharomyces boulardii*]	42.2	547.36	30.99	1	4	9	13	384	5.38
KQC42453.1	Fructose-1,6-bisphosphatase [*Saccharomyces boulardii*]	38.2	546.88	27.59	1	8	8	12	348	6.01
KQC44573.1	eIF3g subunit of the eukaryotic translation initiation factor 3 (eIF3) [*Saccharomyces boulardii*]	30.5	546.03	25.91	1	5	5	11	274	6.81
KQC43553.1	hypothetical protein AB282_02549 [*Saccharomyces boulardii*]	77.4	541.74	18.58	1	10	10	14	689	6.40
KQC45029.1	Peroxisomal malate dehydrogenase [*Saccharomyces boulardii*]	37.2	540.85	32.36	1	8	8	18	343	9.20
KQC43604.1	Glycerol kinase [*Saccharomyces boulardii*]	75.7	537.60	18.20	1	12	12	19	676	6.98
KQC41874.1	Enzyme of 'de novo' purine biosynthesis [*Saccharomyces boulardii*]	65.2	537.32	21.28	1	7	11	14	592	6.55
KQC42761.1	Flavoprotein subunit of succinate dehydrogenase [*Saccharomyces boulardii*]	70.2	533.91	16.09	1	7	8	18	640	7.49
KQC42993.1	Ribonucleotide-diphosphate reductase (RNR), small subunit [*Saccharomyces boulardii*]	46.1	533.65	23.81	1	9	9	14	399	5.19
KQC42297.1	eIF3j component of translation initiation factor 3 (eIF3) [*Saccharomyces boulardii*]	29.5	533.34	31.32	1	6	6	11	265	5.16
KQC44354.1	S-adenosyl-L-homocysteine hydrolase [*Saccharomyces boulardii*]	49.1	529.28	26.73	1	10	10	17	449	6.24
KQC43281.1	Subunit of the 26S proteasome [*Saccharomyces boulardii*]	104.2	528.07	15.56	1	15	16	17	945	6.18
KQC42850.1	Sulfite reductase beta subunit [*Saccharomyces boulardii*]	161.1	528.00	11.23	2	10	11	14	1442	5.49
KQC44656.1	arginyl-tRNA synthetase [*Saccharomyces boulardii*]	69.5	527.67	26.03	2	14	15	18	607	6.74
KQC41614.1	Ribosomal 60S subunit protein L14B [*Saccharomyces boulardii*]	20.6	523.07	39.25	1	7	8	19	186	11.71

KOH51431.1	PSA1p GDP-mannose pyrophosphorylase (mannose-1-phosphate guanyltransferase) [*Saccharomyces boulardii*]	34.1	522.96	39.29	1	1	9	14	308	5.97
KQC44438.1	E3 ubiquitin ligase of NEDD4 family [*Saccharomyces boulardii*]	91.8	521.32	18.79	1	10	10	13	809	6.70
KQC43096.1	Translation initiation factor eIF4A [*Saccharomyces boulardii*]	44.7	521.25	27.09	1	8	8	13	395	5.12
KQC40406.1	ATP-dependent DNA helicase, also known as reptin [*Saccharomyces boulardii*]	51.6	517.35	14.44	1	7	7	19	471	5.31
KQC41413.1	Ribosomal 60S subunit protein L9B [*Saccharomyces boulardii*]	21.6	513.39	42.93	2	9	9	15	191	9.66
KQC42615.1	Carboxy-terminal domain (CTD) phosphatase [*Saccharomyces boulardii*]	60.9	513.04	27.27	1	10	10	12	539	6.60
KQC43337.1	Subunit beta of the cytosolic chaperonin Cct ring complex [*Saccharomyces boulardii*]	57.2	512.31	16.51	1	7	7	10	527	6.11
KQC44575.1	Non-ATPase regulatory subunit of the 26S proteasome [*Saccharomyces boulardii*]	45.8	511.38	24.68	2	12	12	20	393	5.78
KQC40682.1	Transketolase [*Saccharomyces boulardii*]	73.8	510.55	18.68	1	11	11	16	680	7.01
KQC45428.1	histone H4 [*Saccharomyces boulardii*]	11.4	508.74	40.78	1	4	4	15	103	11.36
KQC41719.1	Inosine monophosphate dehydrogenase [*Saccharomyces boulardii*]	56.4	507.88	17.56	1	2	6	11	524	8.00
KQC44094.1	Sensor of mitochondrial dysfunction [*Saccharomyces boulardii*]	65.6	502.79	15.14	1	7	7	10	588	8.12
KQC40653.1	Subunit H of the V1 peripheral membrane domain of V-ATPase [*Saccharomyces boulardii*]	54.4	502.07	20.29	1	8	8	11	478	6.35
KQC44838.1	Vacuolar glutathione S-conjugate transporter [*Saccharomyces boulardii*]	171.0	500.57	5.81	1	6	6	13	1515	8.41
KQC44024.1	High affinity Ca2+/Mn2+ P-type ATPase [*Saccharomyces boulardii*]	104.5	499.37	12.53	1	10	11	14	950	5.68
KQC42384.1	Gamma-glutamyltranspeptidase [*Saccharomyces boulardii*]	69.6	498.43	13.22	1	9	9	22	628	5.60
KQC43746.1	Ribosomal 60S subunit protein L24B [*Saccharomyces boulardii*]	17.5	497.40	29.68	1	1	6	18	155	11.37
KQC45405.1	ACH1p with CoA transferase activity [*Saccharomyces boulardii*]	58.7	494.39	26.62	1	11	11	20	526	6.79
KQC43774.1	Ribosomal protein 28 (rp28) of the small (40S) ribosomal subunit [*Saccharomyces boulardii*]	16.0	489.69	35.86	1	4	4	9	145	10.73
KQC40821.1	eIF3b subunit of the eukaryotic translation initiation factor 3 (eIF3) [*Saccharomyces boulardii*]	83.6	486.89	19.42	1	12	12	16	726	5.59
KQC41155.1	hypothetical protein AB282_04597 [*Saccharomyces boulardii*]	60.9	486.31	24.14	1	9	9	10	551	6.47
KQC41560.1	Glucose-6-phosphate dehydrogenase (G6PD) [*Saccharomyces boulardii*]	57.4	486.15	18.85	1	7	7	10	504	6.43
KQC41215.1	hypothetical protein AB282_04657 [*Saccharomyces boulardii*]	112.5	483.36	9.89	1	7	7	13	991	8.56
KQC42433.1	Acetohydroxyacid reductoisomerase and mtDNA binding protein [*Saccharomyces boulardii*]	44.3	483.30	30.89	1	10	10	13	395	9.04
KQC41946.1	hypothetical protein AB282_04010 [*Saccharomyces boulardii*]	126.5	483.24	11.67	1	10	10	14	1088	5.38
KQC44110.1	Beta 7 subunit of the 20S proteasome [*Saccharomyces boulardii*]	29.4	482.22	27.82	1	5	5	11	266	5.99
KQC40809.1	NADP(+)-dependent glutamate dehydrogenase [*Saccharomyces boulardii*]	49.5	481.30	22.47	1	4	7	11	454	5.69
KQC43909.1	Ribosomal 60S subunit protein L24A [*Saccharomyces boulardii*]	17.6	481.17	29.68	1	1	6	17	155	11.28
KQC40382.1	Translation initiation factor eIF5B [*Saccharomyces boulardii*]	53.1	479.60	23.77	1	8	8	15	488	8.25
KQC42054.1	eIF3c subunit of the eukaryotic translation initiation factor 3 (eIF3) [*Saccharomyces boulardii*]	93.2	477.40	12.93	1	9	9	11	812	5.00

KQC41870.1	G-protein beta subunit and guanine dissociation inhibitor for Gpa2p [*Saccharomyces boulardii*]	34.8	477.27	27.59	1	10	10	14	319	6.24
KQC41476.1	CAP (cyclase-associated protein) [*Saccharomyces boulardii*]	57.5	475.58	24.52	1	9	9	11	526	5.64
KQC42765.1	Tetrameric phosphoglycerate mutase [*Saccharomyces boulardii*]	27.6	475.46	34.82	1	8	8	15	247	8.84
KQC45337.1	Ribosomal 60S subunit protein L32 [*Saccharomyces boulardii*]	14.8	474.10	33.85	1	5	5	13	130	11.17
KQC44845.1	Pentafunctional arom protein [*Saccharomyces boulardii*]	174.7	469.01	10.08	1	12	12	12	1588	6.28
KQC45020.1	Mitochondrial NADP-specific isocitrate dehydrogenase [*Saccharomyces boulardii*]	48.2	463.84	28.50	1	8	10	13	428	8.76
KQC45136.1	ADP-ribosylation factor [*Saccharomyces boulardii*]	20.5	462.96	39.78	1	2	6	11	181	7.34
KQC44095.1	Hexokinase isoenzyme 2 [*Saccharomyces boulardii*]	53.9	462.71	20.16	1	6	6	14	486	5.30
KQC44526.1	Alpha1,2-mannosyltransferase of the Golgi [*Saccharomyces boulardii*]	51.4	462.46	26.47	1	10	11	14	442	5.40
KQC45098.1	Subunit of the CSN and 26S proteasome lid complex [*Saccharomyces boulardii*]	51.8	460.56	22.70	1	9	9	10	445	6.13
KQC41439.1	RPS7Bp component of the small (40S) ribosomal subunit [*Saccharomyces boulardii*]	21.6	459.66	38.95	1	4	7	14	190	9.95
KQC40865.1	NOP58p [*Saccharomyces boulardii*]	56.9	456.80	23.29	2	9	9	11	511	8.94
KQC43984.1	RPS2p component of the small (40S) subunit [*Saccharomyces boulardii*]	27.4	448.76	43.31	1	8	8	15	254	10.43
KQC43776.1	Nucleosome remodeling protein [*Saccharomyces boulardii*]	168.2	448.04	8.75	1	10	10	13	1451	5.00
KQC45491.1	AAA ATPase and SNARE disassembly chaperone [*Saccharomyces boulardii*]	84.0	447.19	19.26	2	10	11	11	758	7.99
KQC44316.1	GTPase [*Saccharomyces boulardii*]	57.6	446.59	23.85	1	10	10	15	520	8.88
KQC44874.1	Essential Fe-S protein [*Saccharomyces boulardii*]	68.3	446.33	22.70	1	12	12	16	608	8.66
KQC45118.1	NAD(+)-dependent glutamate synthase (GOGAT) [*Saccharomyces boulardii*]	238.0	443.63	5.92	2	9	11	14	2145	6.61
KQC40630.1	RNA polymerase I second largest subunit A135 [*Saccharomyces boulardii*]	135.7	443.23	9.48	1	9	9	10	1203	7.65
KQC43624.1	Ribosome-associated chaperone [*Saccharomyces boulardii*]	49.0	441.47	24.71	1	8	8	10	433	8.25
KQC44174.1	Dihydrolipoamide dehydrogenase [*Saccharomyces boulardii*]	54.0	440.87	29.26	1	11	11	13	499	8.03
KQC43839.1	Transaldolase, enzyme in the non-oxidative pentose phosphate pathway [*Saccharomyces boulardii*]	37.2	433.22	27.93	1	8	8	10	333	6.38
KQC41899.1	eIF3i subunit of the eukaryotic translation initiation factor 3 (eIF3) [*Saccharomyces boulardii*]	38.7	433.19	42.94	1	10	10	13	347	5.67
KQC45462.1	RPS11Bp component of the small (40S) ribosomal subunit [*Saccharomyces boulardii*]	17.7	432.25	42.31	1	7	7	12	156	10.78
KQC42419.1	Conserved ribosomal protein P0 of the ribosomal stalk [*Saccharomyces boulardii*]	33.7	432.06	29.49	1	9	9	14	312	4.79
KQC40579.1	One of two isoforms of the gamma subunit of eEF1B [*Saccharomyces boulardii*]	47.1	425.02	20.72	1	5	7	12	415	8.38
KQC40415.1	ATP binding cassette protein [*Saccharomyces boulardii*]	134.3	423.38	12.63	1	12	12	16	1196	5.85
KQC42387.1	O-acetyl homoserine-O-acetyl serine sulfhydrylase [*Saccharomyces boulardii*]	48.7	421.92	25.00	1	10	10	10	444	6.43
KQC41998.1	Glutamate decarboxylase [*Saccharomyces boulardii*]	65.9	421.79	12.14	1	5	5	15	585	6.62
KQC42986.1	Subunit of the cytosolic chaperonin Cct ring complex [*Saccharomyces boulardii*]	58.8	421.05	22.47	1	10	10	12	534	6.11

KQC42277.1	ubiquitin-ribosomal 40S subunit protein S31 fusion protein [*Saccharomyces boulardii*]	17.2	420.54	40.79	4	6	6	11	152	9.86
KQC41589.1	Glyoxylate reductase [*Saccharomyces boulardii*]	38.8	420.26	35.71	1	9	9	11	350	6.34
KQC45591.1	RPS9Ap component of the small (40S) ribosomal subunit [*Saccharomyces boulardii*]	22.3	417.15	32.31	1	1	7	16	195	10.05
KQC40550.1	RPS9Ap component of the small (40S) ribosomal subunit [*Saccharomyces boulardii*]	23.0	415.11	32.18	1	1	7	15	202	9.85
KQC41460.1	Component of the TOM (translocase of outer membrane) complex [*Saccharomyces boulardii*]	70.1	414.57	17.67	1	7	7	9	617	5.35
KQC45088.1	ADP-ribosylation factor [*Saccharomyces boulardii*]	20.6	414.38	32.04	1	1	5	10	181	7.34
KQC42007.1	Regulatory subunit of trehalose-6-phosphate synthase/phosphatase [*Saccharomyces boulardii*]	118.8	412.02	8.25	1	5	6	12	1054	6.33
KQC43888.1	Alpha 1 subunit of the 20S proteasome [*Saccharomyces boulardii*]	28.0	411.47	41.27	1	9	9	10	252	6.24
KQC41721.1	Cytochrome b2 (L-lactate cytochrome-c oxidoreductase) [*Saccharomyces boulardii*]	65.5	409.90	19.63	2	9	10	16	591	8.41
KQC41919.1	Cytoplasmic aldehyde dehydrogenase [*Saccharomyces boulardii*]	55.4	408.31	21.74	1	4	9	9	506	5.76
KQC44059.1	Rab family GTPase involved in the exocytic pathway [*Saccharomyces boulardii*]	24.5	403.60	38.74	1	3	7	9	222	5.49
KQC45135.1	Ribosomal 60S subunit protein L35A [*Saccharomyces boulardii*]	13.9	403.52	41.67	1	6	6	13	120	10.58
KQC43322.1	Catalytic subunit of the SWI/SNF chromatin remodeling complex [*Saccharomyces boulardii*]	156.7	402.58	7.65	1	9	10	13	1359	6.71
KQC45820.1	Subunit of protein N-terminal acetyltransferase NatA [*Saccharomyces boulardii*]	98.9	401.67	13.58	1	12	12	13	854	8.78
KQC45381.1	Major CTP synthase isozyme (see also URA8) [*Saccharomyces boulardii*]	64.7	401.53	13.99	1	6	6	8	579	6.01
KQC40538.1	putative GTPase [*Saccharomyces boulardii*]	74.4	398.72	22.41	1	13	13	14	647	8.79
KQC45267.1	Citrate synthase [*Saccharomyces boulardii*]	51.4	398.68	16.74	1	6	6	9	460	6.38
KQC45574.1	hypothetical protein AB282_00336 [*Saccharomyces boulardii*]	77.6	396.27	18.04	1	7	11	14	693	5.57
KQC44400.1	Threonine deaminase, catalyzes first step in isoleucine biosynthesis [*Saccharomyces boulardii*]	63.8	395.75	13.89	1	5	5	8	576	8.53
KQC41864.1	Hexadecenal dehydrogenase [*Saccharomyces boulardii*]	59.9	395.31	15.23	1	6	6	11	532	6.76
KQC40513.1	putative ATP-dependent RNA helicase of the DEAD-box protein family [*Saccharomyces boulardii*]	67.9	394.62	13.94	1	4	8	12	617	8.87
KQC41898.1	Mitochondrial external NADH dehydrogenase [*Saccharomyces boulardii*]	62.7	393.96	21.96	1	12	12	12	560	9.28
KQC41862.1	Acetolactate synthase [*Saccharomyces boulardii*]	74.8	390.24	16.89	1	9	9	11	687	8.65
KQC40551.1	Ribosomal 60S subunit protein L21B [*Saccharomyces boulardii*]	18.3	390.15	40.00	2	9	9	15	160	10.39
KQC44424.1	Karyopherin beta [*Saccharomyces boulardii*]	122.5	386.29	9.97	1	9	10	12	1113	4.63
KQC43908.1	Ribosomal 60S subunit protein L30 [*Saccharomyces boulardii*]	12.9	386.14	57.26	1	6	6	12	117	9.60
KQC42816.1	Phosphatidylinositol phosphate (PtdInsP) phosphatase [*Saccharomyces boulardii*]	71.1	385.76	17.82	1	8	8	10	623	7.71
KQC45091.1	RPS15p component of the small (40S) ribosomal subunit [*Saccharomyces boulardii*]	187.0	381.52	7.70	3	11	12	12	1689	5.60
KQC43536.1	Delta-1-pyrroline-5-carboxylate dehydrogenase [*Saccharomyces boulardii*]	64.4	376.50	24.87	1	12	12	14	575	7.49

KQC42436.1	Adenylosuccinate lyase [*Saccharomyces boulardii*]	54.5	376.39	19.29	1	9	9	9	482	6.43
KQC44284.1	P-type ATPase, ion transporter of the ER membrane [*Saccharomyces boulardii*]	135.2	376.29	8.81	6	9	10	13	1215	6.60
KQC45723.1	Translation initiation factor eIF5B [*Saccharomyces boulardii*]	112.0	376.06	12.99	1	10	10	10	1001	5.80
KQC44290.1	Ubiquinol-cytochrome-c reductase [*Saccharomyces boulardii*]	23.3	373.73	33.02	1	4	4	8	215	8.07
KQC43762.1	hypothetical protein AB282_02018 [*Saccharomyces boulardii*]	92.6	373.46	10.54	1	7	7	9	816	5.03
KQC45825.1	NPC2p [*Saccharomyces boulardii*]	19.0	371.78	22.54	1	3	3	8	173	4.44
KQC43566.1	Lanosterol 14-alpha-demethylase [*Saccharomyces boulardii*]	60.7	370.97	17.36	1	6	6	10	530	8.66
KQC42380.1	Ran GTPase [*Saccharomyces boulardii*]	24.8	369.63	30.59	2	8	8	11	219	6.55
KQC41447.1	Alpha-isopropylmalate synthase (2-isopropylmalate synthase) [*Saccharomyces boulardii*]	68.4	368.23	15.83	2	7	7	8	619	6.01
KQC42351.1	Glycogen synthase [*Saccharomyces boulardii*]	80.1	367.51	11.49	6	5	8	11	705	6.29
KQC45555.1	NADP+ dependent arabinose dehydrogenase [*Saccharomyces boulardii*]	38.9	366.59	31.10	1	9	9	12	344	5.96
KQC43477.1	hypothetical protein AB282_02473 [*Saccharomyces boulardii*]	40.6	365.37	26.50	1	7	7	7	366	9.91
KQC44199.1	Phosphomannomutase [*Saccharomyces boulardii*]	29.0	364.88	31.50	1	8	8	12	254	5.24
KQC44759.1	Histone H2B [*Saccharomyces boulardii*]	14.2	364.69	25.95	2	4	4	11	131	10.10
KQC41685.1	Alpha 2 subunit of the 20S proteasome [*Saccharomyces boulardii*]	27.1	364.59	21.60	1	4	4	8	250	5.72
KQC41099.1	ATP-dependent DEAD (Asp-Glu-Ala-Asp)-box RNA helicase [*Saccharomyces boulardii*]	53.9	364.42	15.15	1	6	6	8	482	7.43
KQC45467.1	hypothetical protein AB282_00229 [*Saccharomyces boulardii*]	38.7	363.99	16.57	1	3	5	10	344	9.19
KQC40741.1	SPN1p involved in RNA polymerase II transcription [*Saccharomyces boulardii*]	46.1	363.83	22.44	1	6	6	7	410	7.52
KQC43243.1	Regulatory subunit of the cyclic AMP-dependent protein kinase (PKA) [*Saccharomyces boulardii*]	47.2	363.51	23.56	1	7	7	9	416	7.94
KQC41453.1	ATP-dependent RNA helicase of the DEAD-box protein family [*Saccharomyces boulardii*]	60.8	360.25	18.93	1	9	10	14	544	8.66
KQC42967.1	Alpha subunit of the translation initiation factor eIF2 [*Saccharomyces boulardii*]	34.7	359.82	29.61	1	8	8	11	304	5.02
KOH50702.1	SCW11p Cell wall protein like glucanase [*Saccharomyces boulardii*]	56.1	358.94	21.93	2	6	6	9	538	4.51
KOH51324.1	DLD1p D-lactate dehydrogenase [*Saccharomyces boulardii*]	65.2	358.05	17.21	2	7	7	10	587	6.83
KQC42268.1	STM1p required for optimal translation under nutrient stress [*Saccharomyces boulardii*]	30.0	356.53	29.30	1	5	5	9	273	9.66
KQC45550.1	Polypeptide release factor (eRF1) in translation termination [*Saccharomyces boulardii*]	49.0	355.83	19.91	1	6	6	9	437	5.05
KQC44219.1	E1 beta subunit of the pyruvate dehydrogenase (PDH) complex [*Saccharomyces boulardii*]	48.9	355.06	22.80	1	9	9	11	443	8.27
KQC44487.1	Subunit 7 of ubiquinol cytochrome-c reductase (Complex III) [*Saccharomyces boulardii*]	14.6	353.88	37.01	1	3	3	8	127	5.88
KQC42718.1	Cell wall mannoprotein [*Saccharomyces boulardii*]	24.3	352.72	32.22	1	6	6	7	239	4.67
KQC44333.1	Essential non-ATPase regulatory subunit of the 26S proteasome lid [*Saccharomyces boulardii*]	60.3	351.32	17.40	1	7	7	8	523	5.60
KQC42588.1	hypothetical protein AB282_03016 [*Saccharomyces boulardii*]	31.2	349.42	26.86	1	6	6	8	283	8.16

KQC40399.1	Core component of the signal recognition particle (SRP) complex [*Saccharomyces boulardii*]	68.9	346.22	18.20	1	8	8	8	599	8.75
KQC43913.1	Nicotinamidase that converts nicotinamide to nicotinic acid [*Saccharomyces boulardii*]	25.0	344.18	29.63	1	5	5	9	216	6.27
KQC41996.1	Long chain fatty acyl-CoA synthetase [*Saccharomyces boulardii*]	77.2	342.96	14.12	2	9	9	11	694	6.55
KQC42154.1	Cytosolic aspartate aminotransferase involved in nitrogen metabolism [*Saccharomyces boulardii*]	46.0	342.21	19.86	1	7	7	7	418	8.32
KQC45455.1	Gamma subunit of the F1 sector of mitochondrial F1F0 ATP synthase [*Saccharomyces boulardii*]	34.3	341.38	28.30	1	7	7	8	311	9.31
KQC40657.1	Translation initiation factor eIF5 [*Saccharomyces boulardii*]	45.2	340.53	30.62	1	11	11	15	405	5.06
KQC42093.1	Iron-sulfur protein subunit of succinate dehydrogenase [*Saccharomyces boulardii*]	30.2	339.69	27.44	1	7	7	8	266	8.82
KQC42047.1	Integral inner mitochondrial membrane protein [*Saccharomyces boulardii*]	96.6	339.13	5.88	1	3	3	4	850	8.81
KQC42155.1	Enzyme of 'de novo' purine biosynthesis [*Saccharomyces boulardii*]	65.2	338.50	16.75	1	6	10	11	591	6.55
KQC44803.1	Chromatin associated high mobility group (HMG) family member [*Saccharomyces boulardii*]	27.5	338.29	26.02	1	4	4	8	246	8.51
KQC43719.1	glucose deprivation induced stress granule component [*Saccharomyces boulardii*]	78.8	337.43	14.96	1	7	7	8	722	6.90
KQC44359.1	Nuclear type II J heat shock protein [*Saccharomyces boulardii*]	44.8	337.30	21.23	1	6	6	7	391	5.80
KQC41416.1	Dihydrolipoamide acetyltransferase component (E2) of the PDC [*Saccharomyces boulardii*]	51.8	335.34	17.63	1	8	8	11	482	7.80
KQC44843.1	Fimbrin, actin-bundling protein [*Saccharomyces boulardii*]	71.7	335.11	16.82	1	8	8	10	642	5.48
KQC40847.1	Subunit E of the V1 domain of the vacuolar H+-ATPase (V-ATPase) [*Saccharomyces boulardii*]	26.5	334.87	36.48	1	8	8	9	233	5.36
KQC41562.1	Adaptor protein that links actin to clathrin and endocytosis [*Saccharomyces boulardii*]	108.8	334.55	8.99	1	5	5	5	968	5.44
KQC45361.1	Mitochondrial peroxiredoxin with thioredoxin peroxidase activity [*Saccharomyces boulardii*]	29.5	334.55	38.31	1	7	7	8	261	8.87
KQC41846.1	Component of the RSC chromatin remodeling complex [*Saccharomyces boulardii*]	49.6	333.25	19.77	1	7	7	9	435	4.96
KQC42385.1	Major exo-1,3-beta-glucanase of the cell wall [*Saccharomyces boulardii*]	51.3	332.74	11.61	1	4	4	9	448	4.75
KQC41863.1	type I myosin motor [*Saccharomyces boulardii*]	136.5	332.68	8.97	1	6	9	9	1215	9.28
KQC43554.1	Cytosolic asparaginyl-tRNA synthetase [*Saccharomyces boulardii*]	62.2	331.87	20.22	1	8	8	8	554	5.85
KQC40775.1	Glycogen debranching enzyme [*Saccharomyces boulardii*]	174.9	331.47	7.29	1	9	9	10	1536	5.77
KQC42282.1	Pseudouridine synthase catalytic subunit of box H/ACA snoRNPs [*Saccharomyces boulardii*]	55.0	330.28	19.96	1	7	7	7	486	8.94
KQC42568.1	Glycerol-3-phosphate/dihydroxyacetone phosphate sn-1 acyltransferase [*Saccharomyces boulardii*]	83.6	330.26	13.59	1	8	8	12	743	9.48
KQC45083.1	Cullin [*Saccharomyces boulardii*]	94.0	329.74	12.88	1	8	8	14	815	8.32
KQC41190.1	Arginosuccinate synthetase [*Saccharomyces boulardii*]	46.9	328.40	21.43	1	7	7	10	420	5.62
KQC42052.1	Beta-1,3-glucanosyltransferase [*Saccharomyces boulardii*]	59.5	327.04	8.23	1	4	4	6	559	4.67
KQC41521.1	membrane protein [*Saccharomyces boulardii*]	25.1	325.80	41.26	1	7	7	11	223	9.72
KQC45264.1	Amphiphysin-like lipid raft protein [*Saccharomyces boulardii*]	30.2	325.36	23.02	1	5	5	7	265	6.62

KQC42814.1	Ubiquitin activating enzyme (E1) [*Saccharomyces boulardii*]	114.2	325.34	14.75	1	11	11	14	1024	5.11
KQC41704.1	Dihydroxyacetone kinase [*Saccharomyces boulardii*]	62.2	325.21	17.81	1	7	7	8	584	5.41
KQC43587.1	RPS20p component of the small (40S) ribosomal subunit [*Saccharomyces boulardii*]	13.9	324.91	29.75	1	3	3	10	121	9.52
KQC41845.1	hypothetical protein AB282_03909 [*Saccharomyces boulardii*]	24.9	322.48	35.24	1	7	7	8	227	5.80
KQC42231.1	Thiol-specific peroxiredoxin [*Saccharomyces boulardii*]	19.1	322.15	22.16	1	3	3	6	176	5.16
KQC41362.1	Peroxisomal NADP-dependent isocitrate dehydrogenase [*Saccharomyces boulardii*]	47.8	322.14	13.57	1	3	5	7	420	9.22
KQC45228.1	Threonine synthase [*Saccharomyces boulardii*]	57.4	321.72	20.62	2	7	7	7	514	5.64
KQC41654.1	3-hydroxy-3-methylglutaryl-CoA (HMG-CoA) synthase [*Saccharomyces boulardii*]	55.0	320.85	20.37	1	8	8	11	491	8.16
KQC42980.1	Subunit of the cytosolic chaperonin Cct ring complex [*Saccharomyces boulardii*]	61.6	320.42	19.72	1	10	10	11	568	5.64
KQC45613.1	Vacuolar membrane cation-chloride cotransporter (CCC) [*Saccharomyces boulardii*]	57.2	320.23	13.47	1	6	6	12	527	8.88
KQC43733.1	Translation initiation factor eIF4G [*Saccharomyces boulardii*]	107.0	320.02	8.51	1	6	6	9	952	5.99
KQC42934.1	Cytochrome c, isoform 1 [*Saccharomyces boulardii*]	12.2	319.41	40.37	2	5	5	10	109	9.42
KQC44822.1	Cytoplasmic peptidyl-prolyl cis-trans isomerase (cyclophilin) [*Saccharomyces boulardii*]	17.4	317.95	22.22	1	2	2	7	162	7.44
KQC43713.1	TFIIF (Transcription Factor II) largest subunit [*Saccharomyces boulardii*]	82.2	315.27	8.03	1	6	6	9	735	5.72
KQC42042.1	Vacuolar carboxypeptidase Y (proteinase C, CPY) [*Saccharomyces boulardii*]	59.8	314.67	17.86	1	9	9	9	532	4.74
KQC42746.1	type I myosin [*Saccharomyces boulardii*]	142.2	314.33	11.57	1	7	10	10	1270	9.36
KQC40701.1	Ribosomal 60S subunit protein L11A [*Saccharomyces boulardii*]	19.7	313.58	21.26	2	4	4	8	174	9.92
KQC41731.1	Carnitine acetyl-CoA transferase [*Saccharomyces boulardii*]	77.2	311.90	14.03	1	8	9	9	670	8.44
KQC42184.1	Beta subunit of cytoplasmic phenylalanyl-tRNA synthetase [*Saccharomyces boulardii*]	67.3	311.67	17.14	1	10	10	12	595	5.71
KQC44964.1	Neutral trehalase, degrades trehalose [*Saccharomyces boulardii*]	85.9	311.01	13.98	3	9	10	10	751	7.77
KQC43847.1	Ribosomal 60S subunit protein L26A [*Saccharomyces boulardii*]	14.2	309.44	42.52	1	6	7	10	127	10.46
KQC43407.1	NMD3p involved in nuclear export of the large ribosomal subunit [*Saccharomyces boulardii*]	59.1	308.49	13.71	1	6	6	6	518	5.47
KQC40820.1	proteasome core particle subunit alpha 7 [*Saccharomyces boulardii*]	31.5	307.63	22.92	1	6	6	12	288	5.19
KQC40709.1	RNA polymerase subunit AC40 [*Saccharomyces boulardii*]	37.7	306.82	28.36	1	7	7	8	335	5.52
KQC44789.1	Subunit of the cytosolic chaperonin Cct ring complex [*Saccharomyces boulardii*]	59.9	306.07	17.40	1	9	9	10	546	5.86
KQC42459.1	Subunit of Elongator complex [*Saccharomyces boulardii*]	150.9	305.64	8.71	1	8	8	8	1332	5.07
KQC41836.1	Phosphatidylinositol/phosphatidylcholine transfer protein [*Saccharomyces boulardii*]	34.7	304.84	19.54	2	5	5	11	302	5.49
KQC44075.1	SHE10p involved in outer spore wall assembly [*Saccharomyces boulardii*]	67.3	304.70	17.01	1	9	9	11	582	5.86
KQC41727.1	Endoplasmic reticulum (ER) localized integral membrane protein [*Saccharomyces boulardii*]	45.8	304.40	23.57	1	8	8	9	403	7.12
KQC45398.1	ATP-dependent Lon protease [*Saccharomyces boulardii*]	127.1	303.82	6.62	2	6	6	7	1133	5.57

KQC43768.1	Asparagine synthetase [*Saccharomyces boulardii*]	64.6	303.06	10.14	1	4	5	6	572	5.87
KQC45681.1	Vacuolar aminopeptidase Y [*Saccharomyces boulardii*]	60.1	302.53	17.13	1	7	7	10	537	5.31
KQC43707.1	E3-binding protein of the mitochondrial pyruvate dehydrogenase complex [*Saccharomyces boulardii*]	45.3	301.85	9.02	1	2	2	5	410	5.73
KQC44067.1	hypothetical protein AB282_02323 [*Saccharomyces boulardii*]	31.9	301.84	21.88	1	5	5	8	288	7.46
KQC44751.1	Reticulon protein [*Saccharomyces boulardii*]	32.9	300.51	24.07	1	6	6	8	295	9.17
KQC40431.1	Signal recognition particle (SRP) receptor beta subunit [*Saccharomyces boulardii*]	73.5	299.93	11.56	1	7	7	7	640	9.41
KQC43831.1	hypothetical protein AB282_02087 [*Saccharomyces boulardii*]	71.3	299.55	13.40	1	6	6	9	642	8.91
KQC42889.1	Mitochondrial succinate-fumarate transporter [*Saccharomyces boulardii*]	35.3	297.65	20.19	1	6	6	7	322	9.76
KQC41083.1	Cytochrome c1 [*Saccharomyces boulardii*]	34.0	296.32	27.83	1	5	5	6	309	8.12
KQC43903.1	Tryptophan synthase [*Saccharomyces boulardii*]	76.6	292.26	13.30	1	7	7	8	707	6.51
KQC42036.1	ATP-dependent RNA helicase [*Saccharomyces boulardii*]	56.7	292.05	16.63	1	7	7	7	505	9.33
KQC40650.1	Cytoplasmic and mitochondrial histidine tRNA synthetase [*Saccharomyces boulardii*]	59.9	291.41	12.45	1	5	5	6	546	7.71
KQC43131.1	Type I membrane protein involved in autophagy and the Cvt pathway [*Saccharomyces boulardii*]	30.2	290.43	20.66	1	5	5	8	271	5.77
KQC41028.1	Phosphoribosylaminoimidazole carboxylase [*Saccharomyces boulardii*]	62.2	289.97	12.96	1	7	7	7	571	7.17
KQC41601.1	Gamma subunit of coatomer [*Saccharomyces boulardii*]	104.8	289.24	11.98	1	9	9	9	935	5.12
KQC43662.1	Beta subunit of succinyl-CoA ligase [*Saccharomyces boulardii*]	46.9	288.77	21.08	1	6	6	6	427	7.47
KQC41493.1	Cell wall-related secretory glycoprotein [*Saccharomyces boulardii*]	37.3	288.60	18.08	1	4	4	8	354	5.44
KQC45270.1	Component of the septin ring, required for cytokinesis [*Saccharomyces boulardii*]	37.0	287.82	16.46	2	4	6	8	322	5.72
KQC41016.1	Non-essential component of U5 snRNP [*Saccharomyces boulardii*]	35.0	287.16	19.15	1	5	5	6	329	8.66
KQC42789.1	5-phospho-ribosyl-1(alpha)-pyrophosphate synthetase [*Saccharomyces boulardii*]	47.0	286.22	12.65	1	5	5	7	427	5.73
KQC45674.1	Component of the Paf1p complex involved in transcription elongation [*Saccharomyces boulardii*]	51.8	286.01	23.60	1	6	6	7	445	4.93
KQC41965.1	putative 1,3-beta-glucanosyltransferase [*Saccharomyces boulardii*]	56.8	285.83	20.61	1	8	8	8	524	4.86
KQC40815.1	RPS12p component of the small (40S) ribosomal subunit [*Saccharomyces boulardii*]	15.5	285.47	46.15	1	6	6	13	143	4.73
KQC43520.1	Essential protein [*Saccharomyces boulardii*]	42.4	284.96	15.16	2	4	4	8	376	8.48
KQC43981.1	RNA helicase in the DEAH-box family [*Saccharomyces boulardii*]	87.5	284.91	11.34	1	8	8	9	767	6.42
KQC42679.1	RNAPII degradation factor [*Saccharomyces boulardii*]	82.2	284.42	10.51	1	4	4	5	723	4.94
KOH51351.1	CCT4p Subunit of the cytosolic chaperonin Cct ring complex [*Saccharomyces boulardii*]	57.6	283.16	5.49	2	2	2	6	528	7.93
KQC41920.1	Cytoplasmic aldehyde dehydrogenase [*Saccharomyces boulardii*]	55.2	282.24	16.80	1	2	7	7	506	5.60
KQC41415.1	Ribosomal 60S subunit protein L16B [*Saccharomyces boulardii*]	22.2	280.61	33.33	1	4	7	8	198	10.55
KQC43606.1	hypothetical protein AB282_02602 [*Saccharomyces boulardii*]	33.0	279.46	21.09	1	4	4	7	294	5.66
KQC45115.1	Glyceraldehyde-3-phosphate dehydrogenase (GAPDH), isozyme 3 [*Saccharomyces boulardii*]	41.1	279.33	13.73	1	4	4	6	386	6.61

KQC41566.1	RNA polymerase I subunit A49 [*Saccharomyces boulardii*]	46.6	279.32	11.08	1	4	4	7	415	9.54
KQC41487.1	Palmitoylated plasma membrane-bound casein kinase I (CK1) isoform [*Saccharomyces boulardii*]	62.1	278.64	8.79	1	2	4	5	546	9.07
AGZ13693.1	Rep2 protein [*Saccharomyces boulardii*]	29.8	278.11	20.60	2	5	5	7	267	9.32
KQC41270.1	Component of the Paf1p complex involved in transcription elongation [*Saccharomyces boulardii*]	124.7	277.23	6.59	1	7	7	8	1077	6.32
KQC40758.1	GTP-binding protein of the rho subfamily of Ras-like protein [*Saccharomyces boulardii*]	23.1	275.48	32.06	1	4	4	4	209	6.23
KQC43329.1	Ribosomal 60S subunit protein L16A [*Saccharomyces boulardii*]	22.3	275.41	38.69	1	5	8	9	199	10.55
KQC43236.1	hypothetical protein AB282_01312 [*Saccharomyces boulardii*]	48.8	275.02	13.23	8	5	6	9	431	9.44
KQC40711.1	Essential conserved small ribosomal subunit (40s) synthesis factor [*Saccharomyces boulardii*]	101.0	274.79	8.68	1	5	6	7	887	6.47
KQC44383.1	Acetylglutamate kinase and N-acetyl-gamma-glutamyl-phosphate reductase [*Saccharomyces boulardii*]	94.8	274.28	6.95	1	5	5	13	863	8.15
KQC40552.1	Subunit b of the stator stalk of mitochondrial F1F0 ATP synthase [*Saccharomyces boulardii*]	26.5	274.03	26.25	1	7	7	7	240	9.13
KQC44830.1	Subunit (61/68 kDa) of TFIID and SAGA complex [*Saccharomyces boulardii*]	60.1	271.54	14.47	1	6	6	6	532	9.55
KQC41021.1	Subunit of mitochondrial NAD(+)-dependent isocitrate dehydrogenase [*Saccharomyces boulardii*]	39.7	270.18	28.18	1	6	6	9	369	8.48
KQC40596.1	Acetyl-CoA C-acetyltransferase (acetoacetyl-CoA thiolase) [*Saccharomyces boulardii*]	41.7	268.82	24.12	1	8	8	8	398	7.39
KQC43445.1	putative metalloprotease with similarity to zinc carboxypeptidase [*Saccharomyces boulardii*]	49.8	268.81	10.00	1	3	3	7	430	5.24
KQC43603.1	v-SNARE protein involved in Golgi transport [*Saccharomyces boulardii*]	25.4	268.37	31.84	1	5	5	6	223	9.44
KQC44653.1	Low affinity glucose transporter of the major facilitator superfamily [*Saccharomyces boulardii*]	62.5	266.81	16.23	5	3	6	7	567	7.08
KQC42763.1	Mitochondrial NADH-cytochrome b5 reductase [*Saccharomyces boulardii*]	36.7	264.28	21.67	1	5	5	7	323	8.78
KQC42373.1	Endochitinase [*Saccharomyces boulardii*]	58.0	262.20	5.98	1	3	3	6	552	4.51
KQC43123.1	Farnesyl pyrophosphate synthetase [*Saccharomyces boulardii*]	40.5	261.76	13.07	1	4	4	6	352	5.47
KOH49895.1	RPL43Ap Ribosomal 60S subunit protein L43A [*Saccharomyces boulardii*]	10.9	261.76	27.55	3	2	2	7	98	10.40
KQC40975.1	Mitochondrial translation elongation factor Tu [*Saccharomyces boulardii*]	48.0	260.83	15.79	1	5	5	5	437	6.65
KQC40933.1	Ribosomal Protein of the Large subunit [*Saccharomyces boulardii*]	12.2	260.74	37.38	1	1	4	7	107	11.08
KOH48839.1	FPR3p Nucleolar peptidyl-prolyl cis-trans isomerase (PPIase) [*Saccharomyces boulardii*]	46.1	260.54	18.38	2	4	5	7	408	4.49
KOH49719.1	UTP10p Nucleolar protein [*Saccharomyces boulardii*]	199.8	260.19	4.01	3	6	6	6	1769	6.38
KQC44696.1	Subunit 5 of the stator stalk of mitochondrial F1F0 ATP synthase [*Saccharomyces boulardii*]	22.8	259.83	30.19	1	5	5	10	212	9.57
KQC45023.1	hypothetical protein AB282_01101 [*Saccharomyces boulardii*]	22.9	258.35	17.24	1	3	3	4	203	7.14
KQC41318.1	Subunit of the ARP2/3 complex [*Saccharomyces boulardii*]	39.5	257.77	21.35	1	5	5	5	342	6.81
KQC43786.1	Component of several different pre-ribosomal particle [*Saccharomyces boulardii*]	69.8	256.23	11.57	1	6	6	6	605	5.60

KQC43974.1	Subunit (60 kDa) of TFIID and SAGA complex [*Saccharomyces boulardii*]	57.8	254.71	17.83	1	6	6	7	516	5.50
KQC42329.1	Small rho-like GTPase [*Saccharomyces boulardii*]	21.3	254.49	14.66	1	2	2	5	191	6.74
KQC44937.1	MRH1p that localizes primarily to the plasma membrane [*Saccharomyces boulardii*]	36.2	254.13	12.81	1	1	3	8	320	9.19
KQC40624.1	putative 2-methylcitrate dehydratase [*Saccharomyces boulardii*]	57.6	252.43	13.76	1	6	6	8	516	9.07
KQC44787.1	ATP-dependent DNA helicase, also known as pontin [*Saccharomyces boulardii*]	50.4	251.35	15.98	1	6	6	7	463	5.87
KQC40423.1	GTPase, GTP-binding protein of the ARF family [*Saccharomyces boulardii*]	21.4	250.32	28.95	1	4	4	8	190	5.38
KQC45245.1	Negative regulator of WASP-Arp23 complex [*Saccharomyces boulardii*]	96.1	250.03	7.82	1	6	6	6	870	8.59
KQC42411.1	Component of the exomer complex [*Saccharomyces boulardii*]	60.5	249.85	12.34	2	5	5	6	551	4.51
KQC42415.1	RPS25Bp component of the small (40S) ribosomal subunit [*Saccharomyces boulardii*]	12.0	249.78	28.70	2	4	4	8	108	10.32
KQC42743.1	Serine/threonine protein kinase [*Saccharomyces boulardii*]	76.4	249.24	5.74	1	4	4	6	680	6.51
KQC40957.1	Second-largest subunit of RNA polymerase III [*Saccharomyces boulardii*]	129.4	249.14	8.18	1	7	7	7	1149	8.18
KQC43388.1	Farnesyl-diphosphate farnesyl transferase (squalene synthase) [*Saccharomyces boulardii*]	51.7	249.03	20.72	6	8	9	13	444	5.90
KQC45759.1	Member of the p24 family involved in ER to Golgi transport [*Saccharomyces boulardii*]	24.7	248.41	17.81	1	3	3	4	219	7.43
KQC45500.1	Cortical ER protein involved in ER-plasma membrane tethering [*Saccharomyces boulardii*]	105.6	247.95	7.51	1	6	6	7	945	8.95
KQC41455.1	hypothetical protein AB282_04316 [*Saccharomyces boulardii*]	74.1	247.15	10.71	1	6	6	6	644	6.71
KQC40491.1	Ribosomal 60S subunit protein L33A [*Saccharomyces boulardii*]	13.2	246.73	34.48	1	1	4	7	116	11.44
KQC41565.1	hypothetical protein AB282_04426 [*Saccharomyces boulardii*]	86.0	246.18	9.55	1	7	7	9	754	6.65
KQC41039.1	RNA polymerase III largest subunit C160 [*Saccharomyces boulardii*]	162.2	246.00	5.82	1	9	9	10	1460	8.38
KQC44109.1	Delta subunit of the coatomer complex (COPI) [*Saccharomyces boulardii*]	60.6	244.12	10.99	3	6	7	9	546	5.20
KQC40774.1	Dolichol phosphate mannose (Dol-P-Man) synthase of the ER membrane [*Saccharomyces boulardii*]	30.3	243.83	22.85	1	4	4	8	267	7.88
KQC40383.1	hypothetical protein AB282_05017 [*Saccharomyces boulardii*]	62.7	243.02	9.26	1	4	4	5	551	5.12
KQC43173.1	ER associated glutathione S-transferase capable of homodimerization [*Saccharomyces boulardii*]	26.8	241.14	27.78	1	6	6	7	234	6.65
KQC44619.1	Nuclear polyadenylated RNA-binding protein [*Saccharomyces boulardii*]	24.9	240.85	26.55	1	5	5	6	226	11.33
KQC42185.1	RPS22Ap component of the small (40S) ribosomal subunit [*Saccharomyces boulardii*]	13.7	240.28	18.18	1	1	1	3	121	6.14
KQC41392.1	YIP3p [*Saccharomyces boulardii*]	19.4	239.51	30.68	1	5	5	7	176	8.41
KOH49811.1	OST1p Alpha subunit of the oligosaccharyltransferase complex of the ER lumen [*Saccharomyces boulardii*]	54.1	237.95	7.77	2	2	2	3	476	5.12
KOH50965.1	GSY1p Glycogen synthase [*Saccharomyces boulardii*]	80.4	237.60	11.30	2	4	6	7	708	6.02
KQC40756.1	Translation initiation factor eIF-4B [*Saccharomyces boulardii*]	48.5	237.50	28.21	1	8	8	9	436	5.29
KQC45797.1	Beta-isopropylmalate dehydrogenase (IMDH) [*Saccharomyces boulardii*]	38.9	237.47	17.58	2	4	4	5	364	5.76

KQC41252.1	Cytoplasmic malate dehydrogenase [*Saccharomyces boulardii*]	40.7	237.17	15.12	2	6	6	7	377	7.09
KQC44162.1	Subunit of U3-containing Small Subunit (SSU) processome complex [*Saccharomyces boulardii*]	23.5	236.66	25.58	1	3	4	6	215	7.09
KQC41653.1	Component of the RSC chromatin remodeling complex [*Saccharomyces boulardii*]	65.2	236.39	9.12	1	4	4	6	581	7.83
KQC44129.1	Subunit alpha of assimilatory sulfite reductase [*Saccharomyces boulardii*]	114.8	235.64	8.89	1	7	7	8	1035	5.27
KQC41304.1	Saccharopine dehydrogenase (NADP+, L-glutamate-forming) [*Saccharomyces boulardii*]	48.9	235.62	15.47	1	5	5	5	446	5.27
KQC42406.1	Ribosomal 60S subunit protein L38 [*Saccharomyces boulardii*]	8.8	235.39	51.28	1	4	4	7	78	10.93
KQC41767.1	Rab family GTPase [*Saccharomyces boulardii*]	23.0	235.21	29.81	1	4	4	7	208	4.93
KQC43138.1	Alpha-1,2-mannosyltransferase [*Saccharomyces boulardii*]	67.7	234.07	17.09	1	7	7	7	591	6.87
KQC43757.1	Alpha 3 subunit of the 20S proteasome [*Saccharomyces boulardii*]	28.7	233.55	20.16	1	5	5	7	258	5.22
KQC45606.1	putative alpha-1,2-mannosyltransferase [*Saccharomyces boulardii*]	46.4	233.40	20.51	1	7	7	10	395	8.41
KQC42917.1	Essential component of the Arp2/3 complex [*Saccharomyces boulardii*]	49.5	232.41	22.27	1	8	8	8	449	5.80
KQC44342.1	Rab family GTPase [*Saccharomyces boulardii*]	24.5	231.49	30.04	1	1	5	6	223	5.08
KQC44122.1	hypothetical protein AB282_01786 [*Saccharomyces boulardii*]	63.1	231.20	10.41	1	4	4	4	557	5.33
KQC41407.1	rRNA m5C methyltransferase [*Saccharomyces boulardii*]	69.7	230.61	9.87	1	5	5	5	618	5.07
KQC40614.1	TFIID subunit, involved in RNA pol II transcription initiation [*Saccharomyces boulardii*]	108.7	229.98	4.86	2	4	4	4	947	5.92
KQC41303.1	Ubiquitin protease cofactor [*Saccharomyces boulardii*]	57.6	227.97	15.92	1	5	5	6	515	9.11
KQC44704.1	hypothetical protein AB282_00750 [*Saccharomyces boulardii*]	46.3	227.70	17.16	1	5	5	5	408	4.96
KQC42787.1	Golgi membrane protein with similarity to mammalian CASP [*Saccharomyces boulardii*]	77.3	227.27	9.57	1	4	5	6	679	6.05
KQC42317.1	Peptidyl-prolyl cis-trans isomerase (cyclophilin) [*Saccharomyces boulardii*]	42.0	225.86	16.71	1	6	6	7	371	6.16
KQC40646.1	Gamma-adaptin [*Saccharomyces boulardii*]	93.6	225.68	8.53	1	7	7	7	832	5.50
KQC45580.1	Acyl-coenzymeA:ethanol O-acyltransferase [*Saccharomyces boulardii*]	51.2	224.89	19.96	1	8	8	9	451	7.53
KQC41619.1	Dual-specificity kinase [*Saccharomyces boulardii*]	43.1	221.86	17.87	1	7	7	9	375	8.65
KOH47959.1	SIN3p Component of both the Rpd3S and Rpd3L histone deacetylase complexes [*Saccharomyces boulardii*]	174.8	220.15	5.14	2	7	7	8	1536	5.64
KQC40611.1	RRP12p [*Saccharomyces boulardii*]	137.3	218.45	5.94	1	5	6	6	1228	7.03
KQC42228.1	Huge dynein-related AAA-type ATPase (midasin) [*Saccharomyces boulardii*]	558.9	217.08	1.57	3	6	7	7	4910	5.02
KQC40729.1	hypothetical protein AB282_05364 [*Saccharomyces boulardii*]	49.3	215.58	16.78	1	6	6	7	435	4.75
KQC41742.1	Thioredoxin peroxidase [*Saccharomyces boulardii*]	21.6	215.24	29.59	2	5	5	6	196	5.14
KQC44040.1	Subunit IV of cytochrome c oxidase [*Saccharomyces boulardii*]	17.1	214.96	41.94	1	4	4	6	155	6.90
KQC42646.1	Subunit d of the stator stalk of mitochondrial F1F0 ATP synthase [*Saccharomyces boulardii*]	19.8	214.55	36.78	1	4	4	6	174	8.94
KQC44405.1	Anthranilate synthase [*Saccharomyces boulardii*]	56.7	214.07	13.81	1	6	6	7	507	5.86
KQC44264.1	DNA polymerase phi [*Saccharomyces boulardii*]	115.7	213.65	6.36	1	5	5	5	1022	5.30

KQC40643.1	Acid trehalase required for utilization of extracellular trehalose [*Saccharomyces boulardii*]	136.9	212.32	3.88	1	5	5	6	1211	5.40
KQC45798.1	Cysteine desulfurase [*Saccharomyces boulardii*]	54.4	212.09	10.06	1	4	4	6	497	8.19
KQC42482.1	High-affinity copper transporter of the plasma membrane [*Saccharomyces boulardii*]	27.5	211.38	23.24	1	5	5	7	241	7.74
KQC45289.1	Multifunctional enzyme containing phosphoribosyl-ATP pyrophosphatase [*Saccharomyces boulardii*]	84.4	211.34	8.17	3	5	5	6	771	5.24
KQC42320.1	Vacuolar Fe2+/Mn2+ transporter [*Saccharomyces boulardii*]	34.2	210.93	15.84	1	4	4	5	322	5.10
KOH52390.1	SLA1p Cytoskeletal protein binding protein [*Saccharomyces boulardii*]	135.8	210.78	4.98	2	4	4	4	1244	6.30
KQC40404.1	Beta subunit of the translation initiation factor eIF2 [*Saccharomyces boulardii*]	31.6	210.58	30.53	1	7	7	7	285	9.50
KQC44041.1	RPS26Ap component of the small (40S) ribosomal subunit [*Saccharomyces boulardii*]	13.5	210.41	20.17	2	2	2	6	119	10.76
KQC44925.1	Triose phosphate isomerase, abundant glycolytic enzyme [*Saccharomyces boulardii*]	26.8	209.99	22.18	1	4	4	4	248	6.01
KQC40728.1	hypothetical protein AB282_05363 [*Saccharomyces boulardii*]	19.0	209.94	38.15	1	5	5	6	173	9.39
KQC42781.1	Required for 25S rRNA maturation and 60S ribosomal subunit assembly [*Saccharomyces boulardii*]	49.6	209.57	11.03	1	4	4	4	426	6.54
KQC43381.1	Component of the Rix1 complex [*Saccharomyces boulardii*]	86.7	209.10	3.93	1	2	2	4	763	5.62
KQC42367.1	DEAD-box protein required for 27S rRNA processing [*Saccharomyces boulardii*]	68.0	208.63	11.28	1	6	6	6	594	9.19
KQC44571.1	RNA-binding protein [*Saccharomyces boulardii*]	45.4	208.04	15.70	1	5	5	5	414	5.54
KQC44641.1	Golgi-localized protein [*Saccharomyces boulardii*]	62.3	207.68	10.23	1	4	5	6	557	5.71
KQC43074.1	Subunit of the cytosolic chaperonin Cct ring complex [*Saccharomyces boulardii*]	59.7	207.33	8.91	1	4	4	5	550	5.48
KQC45701.1	putative medium-chain alcohol dehydrogenase with similarity to BDH1 [*Saccharomyces boulardii*]	41.6	206.97	21.73	3	7	7	8	382	6.57
KOH49776.1	MTR4p ATP-dependent 3'-5' RNA helicase of the DExD/H family [*Saccharomyces boulardii*]	118.8	206.75	5.84	2	5	5	5	1045	6.35
KQC44990.1	Essential component of the Arp2/3 complex [*Saccharomyces boulardii*]	44.0	206.46	15.35	1	5	5	5	391	5.78
KQC41171.1	Alpha 4 subunit of the 20S proteasome [*Saccharomyces boulardii*]	28.4	205.96	17.72	1	3	3	4	254	7.36
KQC42045.1	Phosphoribosylpyrophosphate amidotransferase (PRPPAT) [*Saccharomyces boulardii*]	56.7	205.81	19.41	1	7	7	9	510	6.28
KQC44161.1	Subunit of vacuolar transporter chaperone (VTC) complex [*Saccharomyces boulardii*]	91.2	203.99	8.10	2	5	5	6	790	8.32
KQC44010.1	Central component of the peroxisomal importomer complex [*Saccharomyces boulardii*]	38.4	203.98	13.78	2	4	4	4	341	4.88
KQC44769.1	Alpha subunit of chaperonin-containing T-complex [*Saccharomyces boulardii*]	60.4	202.99	12.70	1	6	6	7	559	6.49
KQC41903.1	hypothetical protein AB282_03967 [*Saccharomyces boulardii*]	41.7	202.47	14.25	1	3	3	5	365	8.10
KQC42111.1	Component of the polarisome [*Saccharomyces boulardii*]	165.7	201.15	1.74	1	2	2	4	1493	5.15
KQC43480.1	High-affinity glucose transporter [*Saccharomyces boulardii*]	63.9	200.94	13.72	4	2	5	8	576	6.67
KQC42587.1	Histone chaperone [*Saccharomyces boulardii*]	47.8	200.57	11.51	1	3	3	3	417	4.35
KQC40731.1	Asparagine synthetase [*Saccharomyces boulardii*]	64.4	198.53	13.11	1	6	7	7	572	6.11

KQC41033.1	Component of the Paf1 complex [*Saccharomyces boulardii*]	53.9	198.00	15.73	1	5	5	6	464	4.45
KQC43959.1	Nexin-1 [*Saccharomyces boulardii*]	66.9	197.47	5.55	1	3	3	4	577	6.27
KQC40854.1	Gamma-glutamyl phosphate reductase [*Saccharomyces boulardii*]	49.7	196.28	12.72	1	5	5	5	456	5.59
KQC45533.1	hypothetical protein AB282_00295 [*Saccharomyces boulardii*]	56.1	195.81	12.32	1	5	5	5	495	6.09
KQC41005.1	Plasma membrane ATP-binding cassette (ABC) transporter [*Saccharomyces boulardii*]	170.3	195.06	3.84	2	5	5	5	1511	7.88
KQC45204.1	General repressor of transcription [*Saccharomyces boulardii*]	78.2	194.78	9.40	1	6	6	6	713	5.60
KQC44433.1	Integral ER membrane protein, regulates phospholipid metabolism [*Saccharomyces boulardii*]	26.9	194.16	32.79	1	6	6	7	244	4.89
KQC43484.1	RPF1p involved in assembly and export of the large ribosomal subunit [*Saccharomyces boulardii*]	35.1	193.62	13.22	1	4	4	6	295	9.47
KQC41690.1	Alpha-tubulin [*Saccharomyces boulardii*]	49.8	193.11	17.90	1	6	6	7	447	5.07
KQC40916.1	t-SNARE protein required for secretory vesicle-plasma membrane fusion [*Saccharomyces boulardii*]	75.3	193.06	8.30	1	5	5	5	663	5.27
KQC44820.1	Aspartic beta semi-aldehyde dehydrogenase [*Saccharomyces boulardii*]	39.5	192.83	16.44	1	5	5	5	365	6.73
KQC42764.1	dehydratase [*Saccharomyces boulardii*]	37.3	192.62	10.39	1	4	4	5	337	7.55
KQC42103.1	FRA1p involved in negative regulation of iron regulon transcription [*Saccharomyces boulardii*]	84.8	191.50	6.81	1	4	4	4	749	6.87
KQC43963.1	putative GTPase involved in 60S ribosomal subunit biogenesis [*Saccharomyces boulardii*]	72.7	191.14	11.09	1	6	6	7	640	8.59
KQC44823.1	ENT5p containing an N-terminal epsin-like domain [*Saccharomyces boulardii*]	47.3	190.84	16.55	1	6	6	7	411	6.49
KQC44192.1	Beta-tubulin [*Saccharomyces boulardii*]	50.9	189.99	12.47	1	5	5	6	457	4.78
KQC42613.1	hypothetical protein AB282_03041 [*Saccharomyces boulardii*]	78.8	189.98	9.01	2	4	6	6	699	5.15
KQC45619.1	E1 beta subunit of the pyruvate dehydrogenase (PDH) complex [*Saccharomyces boulardii*]	40.0	189.91	16.94	1	5	5	5	366	5.30
KQC45516.1	putative membrane protein [*Saccharomyces boulardii*]	21.1	189.40	32.98	1	5	5	6	188	9.19
KQC41469.1	Component of the TOM (Translocase of Outer Membrane) complex [*Saccharomyces boulardii*]	16.8	188.56	19.74	1	2	2	3	152	4.16
KQC40707.1	Essential non-ATPase regulatory subunit of the 26S proteasome [*Saccharomyces boulardii*]	48.9	187.82	10.49	1	4	4	4	429	5.25
KQC45774.1	SWH1p [*Saccharomyces boulardii*]	134.9	186.95	4.38	2	4	4	5	1188	5.60
KQC42681.1	TMA19p [*Saccharomyces boulardii*]	18.7	186.68	16.17	1	2	2	3	167	4.56
KOH49812.1	PRE3p Beta 1 subunit of the 20S proteasome [*Saccharomyces boulardii*]	23.5	186.56	24.19	2	5	5	6	215	5.90
KQC44502.1	Mitochondrial protein involved in assembly of succinate dehydrogenase [*Saccharomyces boulardii*]	23.8	185.39	19.51	1	3	3	4	205	6.05
KQC41174.1	Nucleolar protein involved in pre-25S rRNA processing [*Saccharomyces boulardii*]	51.9	185.37	15.47	1	5	5	5	459	9.47
KQC42665.1	hypothetical protein AB282_03093 [*Saccharomyces boulardii*]	60.0	185.34	3.82	1	1	1	3	523	5.17
KQC45737.1	PMT2p O-mannosyltransferase of the ER membrane [*Saccharomyces boulardii*]	86.8	184.97	8.30	1	5	5	5	759	7.71
KQC40652.1	GLutamiNe metabolism [*Saccharomyces boulardii*]	41.7	184.32	13.51	1	4	4	4	370	6.34
KQC42756.1	hypothetical protein AB282_03184 [*Saccharomyces boulardii*]	25.1	183.86	28.31	1	4	4	4	219	4.79

KQC44501.1	Cytoplasmic glutaredoxin [*Saccharomyces boulardii*]	15.9	183.82	32.87	1	4	4	6	143	7.28
KQC41828.1	Integral membrane protein [*Saccharomyces boulardii*]	18.7	182.67	14.97	1	2	2	3	167	6.76
KQC45748.1	Cystathionine gamma-lyase [*Saccharomyces boulardii*]	42.5	182.50	12.44	1	4	4	4	394	6.54
KQC45007.1	RNA binding protein required for maturation of tRNA and U6 snRNA [*Saccharomyces boulardii*]	32.1	182.43	14.55	1	3	3	3	275	8.54
KQC45566.1	Cyclin-dependent kinase (CDK) catalytic subunit [*Saccharomyces boulardii*]	34.0	182.37	26.17	2	8	8	9	298	8.35
KOH52280.1	CYC8p General transcriptional co-repressor [*Saccharomyces boulardii*]	106.8	181.63	5.71	2	4	4	4	964	5.05
KQC44148.1	Ubiquitin-specific protease [*Saccharomyces boulardii*]	57.1	181.57	11.02	1	4	4	4	499	7.14
KQC41063.1	Endosomal Rab family GTPase [*Saccharomyces boulardii*]	23.1	181.07	21.90	2	3	4	5	210	5.33
KQC43714.1	Cytoplasmic tyrosyl-tRNA synthetase [*Saccharomyces boulardii*]	44.0	181.06	15.48	1	7	7	8	394	8.27
KQC43030.1	Mitochondrial intermembrane space protein [*Saccharomyces boulardii*]	28.5	180.91	13.49	1	2	2	3	252	5.92
KQC41705.1	Subunit of the heterodimeric FACT complex (Spt16p-Pob3p) [*Saccharomyces boulardii*]	63.0	180.71	8.51	1	4	5	6	552	4.86
KQC44364.1	Aspartate kinase (L-aspartate 4-P-transferase) [*Saccharomyces boulardii*]	57.8	180.30	7.62	1	3	3	4	525	6.61
KQC45526.1	Transketolase [*Saccharomyces boulardii*]	75.0	179.63	9.99	1	5	5	5	681	6.14
KQC43441.1	Palmitoylated plasma membrane-bound casein kinase I (CK1) isoform [*Saccharomyces boulardii*]	61.7	179.61	10.97	1	3	5	6	538	8.98
KQC43642.1	hypothetical protein AB282_01898 [*Saccharomyces boulardii*]	81.1	179.46	10.27	1	7	7	8	701	6.87
KQC41991.1	Citrate and oxoglutarate carrier protein [*Saccharomyces boulardii*]	34.2	179.27	14.97	1	4	4	4	314	9.88
KQC44916.1	Constituent of 66S pre-ribosomal particle [*Saccharomyces boulardii*]	116.6	179.14	5.27	1	4	4	4	1025	5.02
KQC41993.1	Vacuolar membrane zinc transporter [*Saccharomyces boulardii*]	48.3	178.35	11.09	1	3	3	5	442	6.35
KQC40958.1	NOC2p involved in ribosome biogenesis [*Saccharomyces boulardii*]	81.8	178.18	10.67	1	8	8	8	712	8.38
KQC41080.1	VPS45p of the Sec1p/Munc-18 family [*Saccharomyces boulardii*]	76.4	177.03	8.15	1	4	4	4	675	5.19
KQC43462.1	Cytoplasmic aspartyl aminopeptidase with possible vacuole function [*Saccharomyces boulardii*]	53.1	176.28	10.58	1	5	5	7	482	6.81
KQC44233.1	Chromatin remodeler that regulates various aspects of transcription [*Saccharomyces boulardii*]	168.1	175.77	3.41	1	4	4	4	1468	6.35
KQC44149.1	hypothetical protein AB282_01813 [*Saccharomyces boulardii*]	84.9	175.59	10.77	1	7	7	7	752	6.25
KQC40752.1	Type II integral membrane protein [*Saccharomyces boulardii*]	80.1	175.48	7.50	2	4	4	4	720	4.69
KQC43467.1	GGA2p that regulates Arf1p, Arf2p to facilitate Golgi trafficking [*Saccharomyces boulardii*]	64.3	175.42	7.01	1	3	4	5	585	6.49
KOH49014.1	EMP70p Protein with a role in cellular adhesion and filamentous growth [*Saccharomyces boulardii*]	64.7	174.69	6.68	2	2	3	4	569	6.05
KQC41432.1	Topoisomerase II [*Saccharomyces boulardii*]	164.1	174.48	4.41	1	7	7	7	1428	7.14
KQC40540.1	Cytosolic and mitochondrial glutathione oxidoreductase [*Saccharomyces boulardii*]	51.5	174.25	13.92	1	3	3	6	467	7.40
KQC45460.1	NADPH-dependent quinone reductase [*Saccharomyces boulardii*]	37.0	174.17	14.37	1	4	4	6	334	8.95
KQC45246.1	ssDNA-binding protein essential for mitochondrial genome maintenance [*Saccharomyces boulardii*]	15.4	173.93	21.48	1	3	3	4	135	8.34

KQC45717.1	Guanine nucleotide exchange factor for Cdc42p [*Saccharomyces boulardii*]	96.9	172.48	7.03	1	5	5	6	854	8.46
KQC45086.1	Rho GDP dissociation inhibitor [*Saccharomyces boulardii*]	23.1	171.80	15.35	1	2	2	3	202	6.04
KQC44275.1	Guanosine diphosphatase located in the Golgi [*Saccharomyces boulardii*]	56.8	171.53	13.71	1	4	4	4	518	5.35
KQC44452.1	COX15p required for the hydroxylation of heme O to form heme A [*Saccharomyces boulardii*]	54.6	171.51	9.26	1	3	3	4	486	10.17
KQC41441.1	GTP-binding protein [*Saccharomyces boulardii*]	34.7	171.10	15.53	1	3	3	3	322	7.27
KQC45162.1	hypothetical protein AB282_01254 [*Saccharomyces boulardii*]	34.4	170.79	12.62	1	2	2	3	317	8.00
KQC44466.1	Rho GTPase activating protein (RhoGAP) [*Saccharomyces boulardii*]	245.2	170.78	2.68	2	5	5	5	2167	8.46
KQC41208.1	hypothetical protein AB282_04650 [*Saccharomyces boulardii*]	33.5	170.14	14.78	1	3	3	3	291	9.36
KQC42308.1	Epsin-like protein required for endocytosis and actin patch assembly [*Saccharomyces boulardii*]	72.0	169.95	6.18	1	3	3	3	615	5.54
KQC40641.1	Catalytic subunit of the i-AAA protease complex [*Saccharomyces boulardii*]	81.7	169.55	4.69	1	3	3	3	747	7.08
KQC43918.1	General transcription elongation factor TFIIS [*Saccharomyces boulardii*]	34.8	169.11	17.48	1	5	5	5	309	8.87
KOH48940.1	NOC3p Subunit of a nuclear complex with Noc2p and pre-replicative complexes [*Saccharomyces boulardii*]	75.5	169.02	4.83	2	2	3	4	663	5.95
KQC40794.1	Enolase, a phosphopyruvate hydratase [*Saccharomyces boulardii*]	47.3	168.79	9.15	2	4	4	5	437	5.24
KQC44532.1	Activating gamma subunit of the AMP-activated Snf1p kinase complex [*Saccharomyces boulardii*]	72.0	168.56	10.11	1	5	5	5	633	6.65
KQC45379.1	Beta 6 subunit of the 20S proteasome [*Saccharomyces boulardii*]	26.9	167.57	21.16	1	4	4	4	241	6.07
KQC44202.1	Integral membrane component of ER-derived COPII-coated vesicle [*Saccharomyces boulardii*]	50.4	167.31	6.29	1	2	2	2	445	5.97
AGZ13692.1	Rep1 protein, partial [*Saccharomyces boulardii*]	42.9	167.18	11.89	2	3	3	3	370	6.54
KQC43307.1	metalloendopeptidase [*Saccharomyces boulardii*]	77.4	166.42	9.20	1	6	6	6	696	7.50
KQC44315.1	Apyrase with wide substrate specificity [*Saccharomyces boulardii*]	71.8	165.55	3.81	1	1	1	2	630	6.24
KQC40413.1	RNA 5'-triphosphatase involved in mRNA 5' capping [*Saccharomyces boulardii*]	61.8	165.54	5.46	1	2	2	5	549	5.60
KQC44730.1	Catalase A [*Saccharomyces boulardii*]	58.5	164.88	11.07	1	5	5	7	515	7.85
KQC44047.1	hypothetical protein AB282_02303 [*Saccharomyces boulardii*]	47.8	164.67	12.15	1	4	4	5	428	7.75
KQC44053.1	Cell death protease essential for hypochlorite-induced apoptosis [*Saccharomyces boulardii*]	83.1	164.62	6.24	1	4	4	4	737	4.50
KOH49056.1	DIP2p Nucleolar protein [*Saccharomyces boulardii*]	106.2	164.31	5.41	2	4	4	4	943	6.13
KQC45159.1	NAD(+)-dependent glutamate dehydrogenase [*Saccharomyces boulardii*]	124.2	163.84	6.87	3	7	8	8	1092	5.78
KQC44171.1	Co-chaperone that stimulates HSP70 protein Ssc1p ATPase activity [*Saccharomyces boulardii*]	55.5	163.56	10.57	1	4	4	6	511	9.22
KQC44673.1	Subunit e of mitochondrial F1F0-ATPase [*Saccharomyces boulardii*]	10.9	162.76	30.21	1	2	2	3	96	6.10
KQC42592.1	Mitochondrial inner membrane protein [*Saccharomyces boulardii*]	36.8	162.74	9.12	1	2	2	2	362	4.74
KQC42960.1	hypothetical protein AB282_02745 [*Saccharomyces boulardii*]	22.5	162.63	13.64	1	2	2	2	198	8.60
KQC43799.1	Component of the small-subunit processome [*Saccharomyces boulardii*]	140.4	162.57	5.50	1	5	5	5	1237	8.46

KQC43262.1	Cytoplasmic GTPase/eEF2-like factor involved in ribosomal biogenesis [*Saccharomyces boulardii*]	27.9	162.24	21.60	1	3	4	4	250	5.55
KQC43355.1	3-ketoacyl-CoA thiolase with broad chain length specificity [*Saccharomyces boulardii*]	44.7	162.16	15.83	1	4	4	4	417	7.61
KQC45469.1	hypothetical protein AB282_00231 [*Saccharomyces boulardii*]	57.8	161.36	6.99	1	3	3	4	501	6.46
KQC42449.1	Elongase [*Saccharomyces boulardii*]	39.5	161.20	5.22	1	1	1	2	345	9.31
KQC41264.1	mRNA cap binding protein and translation initiation factor eIF4E [*Saccharomyces boulardii*]	24.2	161.05	28.64	1	6	6	7	213	5.49
KQC41363.1	hypothetical protein AB282_04224 [*Saccharomyces boulardii*]	27.5	160.18	14.11	1	4	4	5	241	5.45
KQC44799.1	Membrane anchor subunit of succinate dehydrogenase (SDH) [*Saccharomyces boulardii*]	20.2	160.13	14.92	1	2	2	2	181	8.90
KQC44533.1	hypothetical protein AB282_01274 [*Saccharomyces boulardii*]	25.2	159.74	17.86	1	3	3	3	224	6.54
KQC43862.1	hypothetical protein AB282_02118 [*Saccharomyces boulardii*]	52.9	159.62	17.83	9	6	7	7	471	6.90
KQC42639.1	MRT4p involved in mRNA turnover and ribosome assembly [*Saccharomyces boulardii*]	27.0	159.40	25.85	1	5	5	5	236	8.56
KOH52146.1	RGD1p GTPase-activating protein (RhoGAP) for Rho3p and Rho4p [*Saccharomyces boulardii*]	69.2	159.30	7.11	2	4	4	5	619	8.75
KQC41689.1	D-Arabinono-1,4-lactone oxidase [*Saccharomyces boulardii*]	59.5	158.59	9.13	1	4	4	4	526	6.80
KQC43269.1	hypothetical protein AB282_01345 [*Saccharomyces boulardii*]	36.0	158.18	4.89	1	1	1	2	327	6.29
KQC42361.1	Non-essential hydrolase involved in mRNA decapping [*Saccharomyces boulardii*]	40.7	157.90	13.71	1	4	4	4	350	6.25
KQC41699.1	HMG-CoA reductase [*Saccharomyces boulardii*]	115.6	157.81	6.55	1	5	5	6	1054	8.00
KQC43126.1	Vacuolar carboxypeptidase S [*Saccharomyces boulardii*]	64.5	157.38	10.42	1	6	6	6	576	5.53
KQC43280.1	3-phosphoglycerate dehydrogenase [*Saccharomyces boulardii*]	51.2	157.18	13.86	2	5	5	5	469	6.54
KOH51417.1	BRE1p E3 ubiquitin ligase [*Saccharomyces boulardii*]	80.6	156.56	6.57	2	3	3	3	700	6.43
KQC45451.1	Pyridoxine (pyridoxamine) phosphate oxidase [*Saccharomyces boulardii*]	26.9	156.53	16.23	1	3	3	4	228	7.49
KQC45271.1	RER1p involved in retention of membrane protein [*Saccharomyces boulardii*]	20.1	156.48	20.24	2	3	3	4	168	8.97
KQC43314.1	Non-essential small GTPase of the Rho/Rac family of Ras-like protein [*Saccharomyces boulardii*]	25.3	155.49	19.91	2	4	4	4	231	5.00
KQC40545.1	Subunit of Elongator complex [*Saccharomyces boulardii*]	63.6	155.15	6.46	1	3	3	5	557	9.00
KQC45339.1	Methionine aminopeptidase [*Saccharomyces boulardii*]	47.5	155.14	5.23	1	2	2	3	421	6.24
KQC42059.1	Alpha 6 subunit of the 20S proteasome [*Saccharomyces boulardii*]	25.6	155.04	23.50	1	4	5	5	234	7.39
KQC43460.1	Ubiquitin-protein ligase (E3) [*Saccharomyces boulardii*]	46.1	154.11	2.64	2	1	1	2	416	6.79
KQC40772.1	GTPase-activating protein, stimulates the GTPase activity of Sar1p [*Saccharomyces boulardii*]	85.3	154.01	6.64	1	4	4	4	768	5.66
KQC44514.1	Pumilio domain protein [*Saccharomyces boulardii*]	75.0	153.89	4.42	1	3	3	4	656	6.89
KQC41477.1	Subunit of the THO complex [*Saccharomyces boulardii*]	183.8	153.79	1.63	1	2	2	3	1597	7.59
KQC43177.1	Saccharopine dehydrogenase (NAD+, L-lysine-forming) [*Saccharomyces boulardii*]	41.4	152.09	11.26	1	3	4	4	373	9.10
KQC41504.1	Plasma membrane protein [*Saccharomyces boulardii*]	40.3	151.96	8.20	1	2	2	4	366	5.80
KQC42286.1	hypothetical protein AB282_03467 [*Saccharomyces boulardii*]	22.1	151.20	19.90	1	3	3	3	201	5.15

KOH49830.1	ESS1p Peptidylprolyl-cis/trans-isomerase (PPIase) [*Saccharomyces boulardii*]	19.2	151.07	22.35	2	3	3	3	170	8.72
KOH52405.1	NCL1p S-adenosyl-L-methionine-dependent tRNA: m5C-methyltransferase [*Saccharomyces boulardii*]	77.5	150.26	5.28	2	2	2	2	682	7.23
KQC43574.1	Oxysterol-binding protein [*Saccharomyces boulardii*]	49.8	149.93	5.26	2	2	2	5	437	7.23
KQC45501.1	Subunit of heteropentameric Replication factor C (RF-C) [*Saccharomyces boulardii*]	39.9	148.73	9.89	1	3	3	3	354	8.05
KQC43530.1	NADP-cytochrome P450 reductase [*Saccharomyces boulardii*]	76.7	148.58	5.93	1	4	4	4	691	5.19
KQC40418.1	Hydrophilin essential in desiccation-rehydration process [*Saccharomyces boulardii*]	18.9	148.47	31.74	1	3	3	4	167	4.82
KQC41400.1	Subunit Va of cytochrome c oxidase [*Saccharomyces boulardii*]	17.1	148.07	26.14	1	3	3	3	153	9.82
KQC42981.1	Essential subunit of U3-containing 90S preribosome [*Saccharomyces boulardii*]	77.7	147.58	5.26	1	3	3	3	666	8.15
KQC44552.1	Guanylate kinase [*Saccharomyces boulardii*]	20.6	146.84	21.39	1	4	4	4	187	7.18
KOH49799.1	BBC1p Protein [*Saccharomyces boulardii*]	126.1	146.76	4.05	2	4	4	5	1136	5.26
KQC40692.1	Transcription factor TFIIB [*Saccharomyces boulardii*]	38.1	146.43	9.86	1	2	2	2	345	8.60
KQC42109.1	hypothetical protein AB282_03289 [*Saccharomyces boulardii*]	32.2	146.37	15.05	2	4	4	6	279	9.96
KQC40991.1	RPS28Ap component of the small (40S) ribosomal subunit [*Saccharomyces boulardii*]	7.6	146.23	31.34	2	2	2	4	67	10.78
KQC42116.1	ABC type transmembrane transporter of MRP/CFTR family [*Saccharomyces boulardii*]	176.9	146.18	2.57	1	3	3	3	1559	7.65
KQC42848.1	Homoserine dehydrogenase (L-homoserine:NADP oxidoreductase) [*Saccharomyces boulardii*]	38.5	146.09	12.26	1	4	4	4	359	7.44
KQC44878.1	Essential evolutionarily conserved nucleolar protein [*Saccharomyces boulardii*]	33.2	145.01	8.63	1	2	2	7	278	5.29
KQC43583.1	5-phospho-ribosyl-1(alpha)-pyrophosphate synthetase [*Saccharomyces boulardii*]	35.1	144.59	8.44	1	2	2	3	320	8.29
KQC42570.1	Constituent of the TIM23 complex [*Saccharomyces boulardii*]	21.9	144.10	16.24	1	2	2	2	197	9.67
KQC41780.1	C-22 sterol desaturase [*Saccharomyces boulardii*]	61.3	143.93	10.22	1	5	5	6	538	7.91
KQC40693.1	Signal recognition particle (SRP) subunit [*Saccharomyces boulardii*]	59.6	143.43	10.72	1	5	5	5	541	8.60
KQC44216.1	hypothetical protein AB282_01738 [*Saccharomyces boulardii*]	27.7	143.18	9.84	1	2	2	3	244	9.70
KQC45660.1	Mitochondrial serine hydroxymethyltransferase [*Saccharomyces boulardii*]	53.7	142.08	10.61	1	4	5	5	490	8.72
KQC41087.1	Alpha' catalytic subunit of casein kinase 2 (CK2) [*Saccharomyces boulardii*]	39.4	142.03	10.03	1	2	2	3	339	8.53
KQC43925.1	COPII-coated vesicle protein [*Saccharomyces boulardii*]	15.9	141.92	7.97	1	1	1	4	138	6.92
KQC40900.1	Constituent of 66S pre-ribosomal particle [*Saccharomyces boulardii*]	51.3	141.91	5.87	1	2	2	3	460	7.14
KQC42750.1	clathrin Adaptor Protein complex Large chain [*Saccharomyces boulardii*]	81.8	141.77	11.16	1	8	8	9	726	5.24
KQC42628.1	Poly(A) polymerase [*Saccharomyces boulardii*]	59.9	141.72	7.02	2	3	3	3	527	8.03
KQC44690.1	Peptidyl-prolyl cis-trans isomerase (cyclophilin) of the ER [*Saccharomyces boulardii*]	25.3	141.55	22.67	1	4	4	4	225	5.60
KQC41952.1	C-8 sterol isomerase [*Saccharomyces boulardii*]	24.9	141.44	17.12	1	3	3	5	222	5.91
KQC42360.1	v-SNARE protein involved in Golgi transport [*Saccharomyces boulardii*]	25.0	141.27	11.21	1	2	2	2	214	7.27

KQC41707.1	ERV46p [*Saccharomyces boulardii*]	40.7	141.26	4.55	1	1	1	1	352	4.89
KQC40592.1	hypothetical protein AB282_05227 [*Saccharomyces boulardii*]	92.5	141.10	5.55	1	4	4	4	829	8.72
KQC44116.1	Cys-Gly metallo-di-peptidase [*Saccharomyces boulardii*]	52.9	140.06	4.57	1	2	2	4	481	5.67
KQC44767.1	Co-chaperone that binds Hsp82p and activates its ATPase activity [*Saccharomyces boulardii*]	39.4	139.81	7.43	1	2	2	4	350	7.47
KQC43214.1	ER protein with chaperone and co-chaperone activity [*Saccharomyces boulardii*]	72.7	138.95	10.85	2	4	5	5	627	6.20
KQC44750.1	Homoaconitase [*Saccharomyces boulardii*]	75.1	138.77	6.93	1	3	3	3	693	7.37
KQC40409.1	TSR1p required for processing of 20S pre-rRNA in the cytoplasm [*Saccharomyces boulardii*]	33.1	138.33	7.93	1	1	2	2	290	5.24
KQC44335.1	Delta 1-pyrroline-5-carboxylate reductase [*Saccharomyces boulardii*]	30.1	137.77	11.89	1	3	3	3	286	5.64
KQC43602.1	Scaffold protein [*Saccharomyces boulardii*]	210.3	137.49	2.57	1	4	4	4	1868	6.55
KQC40505.1	Histone H1, linker histone with roles in meiosis and sporulation [*Saccharomyces boulardii*]	27.8	137.48	10.08	1	3	3	3	258	10.21
KQC43308.1	Component of the Sec23p-Sec24p heterodimer of the COPII vesicle coat [*Saccharomyces boulardii*]	103.6	137.39	3.89	1	3	3	3	926	6.23
KQC42514.1	Subunit d of the V0 integral membrane domain of V-ATPase [*Saccharomyces boulardii*]	39.8	137.34	12.17	1	3	3	4	345	4.60
KQC40835.1	Transcription factor IIIA (TFIIIA) [*Saccharomyces boulardii*]	55.2	137.12	5.34	1	1	2	3	506	7.09
KQC42081.1	hypothetical protein AB282_03261 [*Saccharomyces boulardii*]	32.1	136.83	17.45	2	5	5	5	298	7.15
KQC42393.1	IMH1p involved in vesicular transport [*Saccharomyces boulardii*]	105.3	135.89	2.41	4	1	3	4	911	5.63
KQC45599.1	Subunit (90 kDa) of TFIID and SAGA complex [*Saccharomyces boulardii*]	88.9	135.79	3.63	1	2	2	3	798	7.40
KQC43703.1	SNG1p involved in resistance to nitrosoguanidine and 6-azauracil [*Saccharomyces boulardii*]	61.3	135.64	4.75	1	2	2	3	547	8.73
KQC41723.1	Plasma membrane protein [*Saccharomyces boulardii*]	33.8	135.28	22.85	1	4	4	4	302	7.47
KQC44050.1	Component of the p24 complex [*Saccharomyces boulardii*]	23.3	134.77	15.27	1	3	3	4	203	6.79
KQC42869.1	zinc metalloprotease [*Saccharomyces boulardii*]	52.3	134.60	8.83	1	4	4	5	453	7.91
KQC43879.1	C-3 sterol dehydrogenase [*Saccharomyces boulardii*]	38.7	134.02	8.60	1	3	3	4	349	6.68
KQC44784.1	Mitochondrial transcription elongation factor [*Saccharomyces boulardii*]	76.2	133.75	6.93	1	4	5	5	664	8.98
KQC40950.1	hypothetical protein AB282_04880 [*Saccharomyces boulardii*]	21.2	133.47	15.68	1	3	3	3	185	9.60
KQC42398.1	3-phosphoserine aminotransferase [*Saccharomyces boulardii*]	60.0	133.47	7.12	1	3	3	3	520	5.48
KQC43575.1	Ribosomal 60S subunit protein L14B [*Saccharomyces boulardii*]	15.1	133.41	25.36	1	4	4	5	138	10.93
KQC40993.1	Dynamin-like GTPase that mediates homotypic ER fusion [*Saccharomyces boulardii*]	89.4	133.33	4.64	1	3	3	3	776	5.29
KQC44170.1	Plasma membrane protein involved in maintaining membrane organization [*Saccharomyces boulardii*]	11.6	132.91	24.77	1	2	2	2	109	5.60
KQC43468.1	Component of the septin ring that is required for cytokinesis [*Saccharomyces boulardii*]	46.6	132.17	11.79	1	4	4	4	407	7.99
KQC41883.1	Glycoprotein subunit of transmembrane ring of nuclear pore complex [*Saccharomyces boulardii*]	151.5	131.98	3.52	1	3	3	3	1337	6.74
KQC44435.1	ADP-ribosylation factor GTPase activating protein (ARF GAP) [*Saccharomyces boulardii*]	55.0	131.97	6.69	1	3	4	4	493	8.05

KQC44850.1	Serine/threonine protein kinase involved in regulation of exocytosis [*Saccharomyces boulardii*]	120.0	131.68	3.48	1	3	3	3	1064	8.66
KQC44757.1	Adenylate kinase, required for purine metabolism [*Saccharomyces boulardii*]	24.2	131.57	15.77	1	3	4	4	222	6.70
KQC40904.1	HRK1p kinase [*Saccharomyces boulardii*]	85.0	131.14	8.36	1	4	4	4	754	7.37
KQC43483.1	GAR1p component of the H/ACA snoRNP pseudouridylase complex [*Saccharomyces boulardii*]	21.4	130.87	19.02	1	4	4	4	205	11.46
KQC41130.1	Glutamine-rich cytoplasmic cochaperone [*Saccharomyces boulardii*]	37.2	130.21	15.03	1	4	4	4	346	4.79
KQC45641.1	ATPase subunit [*Saccharomyces boulardii*]	123.8	130.11	4.68	5	4	5	5	1069	6.33
KQC42472.1	Ski complex component and putative RNA helicase [*Saccharomyces boulardii*]	145.8	129.99	2.49	1	2	2	2	1287	6.87
KQC43555.1	Argininosuccinate lyase [*Saccharomyces boulardii*]	52.0	129.89	7.34	1	3	3	3	463	5.73
KQC42676.1	Plasma membrane protein that may act to generate normal levels of PI4P [*Saccharomyces boulardii*]	40.5	128.60	3.97	1	1	1	2	353	7.27
KQC45277.1	Poly(A+) RNA-binding protein [*Saccharomyces boulardii*]	59.4	127.93	9.98	1	4	4	4	521	5.94
KQC44674.1	Mitochondrial ribosomal protein of the large subunit' [*Saccharomyces boulardii*]	42.8	127.11	7.63	1	2	2	4	367	9.60
KQC44808.1	Guanine nucleotide exchange factor (GEF) for ADP ribosylation factor [*Saccharomyces boulardii*]	227.0	127.02	2.14	2	4	5	5	2011	4.79
KQC45722.1	Member of the DRG family of GTP-binding protein [*Saccharomyces boulardii*]	40.7	126.70	18.97	1	5	6	6	369	8.34
KQC42644.1	URB1p [*Saccharomyces boulardii*]	203.3	126.36	2.44	1	3	3	4	1764	7.40
KQC45431.1	hypothetical protein AB282_00193 [*Saccharomyces boulardii*]	22.5	126.15	11.82	1	2	2	2	203	6.15
KQC45374.1	Scaffold protein [*Saccharomyces boulardii*]	150.6	126.13	3.04	1	3	3	3	1381	4.70
KQC43904.1	Glucosidase II catalytic subunit [*Saccharomyces boulardii*]	86.5	126.12	4.41	2	3	3	3	748	5.06
KQC41553.1	Phosphatidylinositol/phosphatidylcholine transfer protein [*Saccharomyces boulardii*]	40.7	125.95	10.26	1	3	3	3	351	7.96
KQC42803.1	Mitochondrial oxidoreductase [*Saccharomyces boulardii*]	40.8	125.93	6.99	1	2	2	2	372	4.39
KQC44323.1	Beta 4 subunit of the 20S proteasome [*Saccharomyces boulardii*]	22.5	125.91	19.19	1	4	4	4	198	6.23
KQC41928.1	hypothetical protein AB282_03992 [*Saccharomyces boulardii*]	31.1	125.42	6.20	1	1	1	1	274	6.05
KQC42159.1	Component of the RSC chromatin remodeling complex [*Saccharomyces boulardii*]	57.8	125.24	9.96	1	4	4	4	502	8.16
KQC41668.1	2-hexaprenyl-6-methoxy-1,4-benzoquinone methyltransferase [*Saccharomyces boulardii*]	34.7	125.24	11.07	1	2	2	3	307	6.57
KQC41848.1	Nucleolar protein [*Saccharomyces boulardii*]	57.7	125.07	7.80	1	3	3	3	513	9.31
KQC43977.1	AMP-activated serine/threonine protein kinase [*Saccharomyces boulardii*]	36.4	125.06	10.87	1	3	3	3	322	5.78
KOH52513.1	CCR4p Component of the CCR4-NOT transcriptional complex [*Saccharomyces boulardii*]	94.6	124.65	4.18	2	3	3	3	837	7.01
KQC44498.1	hypothetical protein AB282_00889 [*Saccharomyces boulardii*]	55.9	123.30	9.20	1	5	5	5	500	6.16
KQC43294.1	Mitochondrial ribosomal protein of the small subunit [*Saccharomyces boulardii*]	30.5	123.11	4.92	1	1	1	2	264	6.25
KQC40961.1	Imidazoleglycerol-phosphate dehydratase [*Saccharomyces boulardii*]	23.8	122.80	13.18	1	2	2	2	220	6.39
KOH50681.1	PMC1p Vacuolar Ca2+ ATPase involved in depleting cytosol of Ca2+ ions [*Saccharomyces boulardii*]	130.7	122.80	3.07	2	3	3	3	1173	7.65

KQC44611.1	Actin-associated protein [*Saccharomyces boulardii*]	52.7	122.59	7.47	1	3	3	3	482	6.01
KQC42505.1	L-ornithine transaminase (OTAse) [*Saccharomyces boulardii*]	46.1	122.32	5.19	1	2	2	2	424	6.95
KQC45017.1	Transport adaptor or symportin [*Saccharomyces boulardii*]	78.2	122.30	2.62	1	2	2	3	688	5.88
KQC45678.1	Subunit of the Ssh1 translocon complex [*Saccharomyces boulardii*]	53.3	122.22	4.69	1	2	2	3	490	8.09
KQC43700.1	hypothetical protein AB282_01956 [*Saccharomyces boulardii*]	26.3	121.78	10.22	1	2	2	2	225	5.55
KQC43533.1	Subunit G of the V1 peripheral membrane domain of V-ATPase [*Saccharomyces boulardii*]	12.7	121.49	21.05	1	2	2	2	114	8.98
KQC43304.1	Phosphoinositide PI4,5P(2) binding protein, forms a complex with Slm2p [*Saccharomyces boulardii*]	77.9	121.39	4.37	1	3	3	4	686	7.94
KQC43412.1	Component of U4/U6-U5 snRNP complex [*Saccharomyces boulardii*]	279.3	120.95	1.41	1	3	3	4	2413	7.46
KQC45818.1	Nuclear thiol peroxidase [*Saccharomyces boulardii*]	16.3	120.72	21.38	2	3	3	3	145	9.94
KQC45141.1	Mitochondrial GTP/GDP transporter [*Saccharomyces boulardii*]	33.2	120.63	16.67	1	5	5	5	300	10.02
KQC45429.1	hypothetical protein AB282_00191 [*Saccharomyces boulardii*]	15.3	120.46	11.76	1	2	2	4	136	11.43
KQC43077.1	NCA3p involved in mitochondrion organization [*Saccharomyces boulardii*]	35.4	119.82	8.61	1	2	2	2	337	4.46
KQC43736.1	Nucleolar protein [*Saccharomyces boulardii*]	44.7	119.78	9.86	1	3	3	3	416	4.93
KQC41355.1	Nucleolar protein [*Saccharomyces boulardii*]	36.5	119.70	15.22	4	5	5	5	322	9.45
KQC41640.1	Histone deacetylase, component of both the Rpd3S and Rpd3L complex [*Saccharomyces boulardii*]	48.9	119.60	7.39	1	2	2	2	433	5.52
KQC43639.1	YTA7p that localizes to chromatin [*Saccharomyces boulardii*]	157.4	119.51	3.26	1	4	4	4	1379	5.19
KQC43491.1	Nuclear exosome-associated nucleic acid binding protein [*Saccharomyces boulardii*]	21.0	119.49	10.33	1	2	2	3	184	9.58
KQC44836.1	Subunit of THO/TREX complex [*Saccharomyces boulardii*]	87.8	119.25	3.86	1	2	2	2	752	5.22
KQC42060.1	hypothetical protein AB282_04124 [*Saccharomyces boulardii*]	38.2	119.20	4.30	1	1	1	1	349	6.70
KQC44397.1	Nucleolar protein [*Saccharomyces boulardii*]	62.3	118.49	4.87	1	2	2	2	554	9.45
KQC41475.1	Mitochondrial ribosomal component of the small subunit [*Saccharomyces boulardii*]	56.3	118.44	9.26	1	4	4	4	486	9.64
KQC43135.1	Subunit of a Golgi mannosyltransferase complex [*Saccharomyces boulardii*]	47.8	118.27	9.95	1	4	4	4	422	8.53
KQC42497.1	Coronin [*Saccharomyces boulardii*]	72.5	118.03	5.53	1	3	3	3	651	5.73
KQC41718.1	Calcineurin A [*Saccharomyces boulardii*]	68.5	117.93	4.14	1	2	2	3	604	6.43
KQC42407.1	hypothetical protein AB282_03589 [*Saccharomyces boulardii*]	27.4	117.46	9.58	1	2	2	3	240	9.36
KQC41770.1	foot domain of RNA polymerase II [*Saccharomyces boulardii*]	59.6	117.02	4.89	1	2	2	3	511	5.50
KQC41738.1	RAD52p that stimulates strand exchange [*Saccharomyces boulardii*]	54.5	116.89	3.26	1	1	1	2	491	8.78
KQC45036.1	Mitochondrial external NADH dehydrogenase [*Saccharomyces boulardii*]	61.6	116.87	4.59	1	3	3	3	545	8.69
KQC43760.1	Subunit of the prohibitin complex (Phb1p-Phb2p) [*Saccharomyces boulardii*]	31.4	116.51	7.67	1	2	2	2	287	8.25
KQC45814.1	fusion protein, partial [*Saccharomyces boulardii*]	164.6	116.34	2.84	16	3	3	3	1446	7.87
KQC44231.1	Aminophospholipid translocase (flippase) [*Saccharomyces boulardii*]	177.7	116.19	1.91	1	2	2	2	1571	6.00
KOH49794.1	VPS53p Component of the GARP (Golgi-associated retrograde protein) complex [*Saccharomyces boulardii*]	95.4	116.17	2.19	2	1	1	1	822	5.59
KQC43292.1	Nucleolar protein [*Saccharomyces boulardii*]	84.0	116.07	4.16	1	2	2	2	721	6.11

KQC44702.1	Signal recognition particle (SRP) receptor alpha subunit [*Saccharomyces boulardii*]	69.1	114.93	3.54	1	1	1	3	621	7.17
KQC44038.1	hydroxyacid dehydrogenase [*Saccharomyces boulardii*]	43.0	114.85	5.01	6	1	2	3	379	7.99
KQC41825.1	UBX domain-containing protein that interacts with Cdc48p [*Saccharomyces boulardii*]	47.0	114.75	10.10	1	3	3	3	416	8.84
KOH47961.1	TOP1p Topoisomerase I [*Saccharomyces boulardii*]	89.9	114.69	6.24	2	5	5	6	769	8.73
KQC43579.1	Cdc42p-activated signal transducing kinase [*Saccharomyces boulardii*]	102.4	114.33	2.45	1	2	2	3	939	7.33
KQC44376.1	hypothetical protein AB282_01632 [*Saccharomyces boulardii*]	27.8	114.11	14.40	1	2	3	3	250	6.16
KQC41225.1	Subunit of heteropentameric Replication factor C (RF-C) [*Saccharomyces boulardii*]	36.1	114.10	8.36	1	2	2	2	323	9.10
KQC40875.1	Transcriptional coactivator [*Saccharomyces boulardii*]	16.4	114.07	31.79	1	4	4	4	151	10.61
KQC44615.1	Plasma membrane protein, putative ammonium transporter [*Saccharomyces boulardii*]	30.0	113.84	6.55	1	2	2	3	275	4.77
KQC42381.1	ATP synthase [*Saccharomyces boulardii*]	14.1	113.84	11.29	1	1	1	2	124	4.68
KQC45108.1	Cytoplasmic DExD/H-box helicase, stimulates mRNA decapping [*Saccharomyces boulardii*]	57.5	112.82	8.30	1	3	3	3	506	7.97
KQC45302.1	hypothetical protein AB282_00574 [*Saccharomyces boulardii*]	87.1	112.53	3.55	1	3	3	4	760	5.35
KQC44087.1	Subunit of RNAPII-associated chromatin remodeling Paf1 complex [*Saccharomyces boulardii*]	65.8	112.40	6.81	1	4	4	4	558	5.39
KQC41193.1	5-phospho-ribosyl-1(alpha)-pyrophosphate synthetase [*Saccharomyces boulardii*]	53.5	111.86	3.63	1	1	1	1	496	7.28
KQC43653.1	Alpha 5 subunit of the 20S proteasome [*Saccharomyces boulardii*]	28.6	111.83	14.23	1	3	3	3	260	4.73
KQC43320.1	Bifunctional triacylglycerol lipase and 1-acyl DHAP reductase [*Saccharomyces boulardii*]	32.8	111.80	9.43	1	2	2	2	297	9.16
KQC40997.1	hypothetical protein AB282_04927 [*Saccharomyces boulardii*]	60.2	111.42	6.49	1	3	3	4	539	8.29
KQC40370.1	NAD(+)-dependent formate dehydrogenase [*Saccharomyces boulardii*]	38.4	111.33	17.63	3	4	4	5	346	7.20
KOH52083.1	PAT1p Deadenylation-dependent mRNA-decapping factor [*Saccharomyces boulardii*]	88.5	111.26	5.90	2	4	5	5	796	7.39
KQC42819.1	5-oxoprolinase [*Saccharomyces boulardii*]	140.4	111.22	3.03	1	3	3	3	1286	6.77
KQC41123.1	Member of the p24 family involved in ER to Golgi transport [*Saccharomyces boulardii*]	23.5	111.17	12.08	1	2	2	2	207	6.79
KQC45705.1	Peroxisomal integral membrane protein [*Saccharomyces boulardii*]	19.9	111.05	10.56	1	1	1	1	180	8.05
KQC43944.1	RNA-Dependent ATPase, member of DExD/H-box family [*Saccharomyces boulardii*]	58.8	110.80	5.16	3	2	3	3	523	9.13
KQC42617.1	Endosomal Rab family GTPase [*Saccharomyces boulardii*]	26.1	110.73	13.25	2	2	3	3	234	4.82
KQC41165.1	1,3-beta-glucanosyltransferase [*Saccharomyces boulardii*]	51.9	110.64	8.68	1	3	3	4	484	4.64
KQC41470.1	KRE33p required for biogenesis of the small ribosomal subunit [*Saccharomyces boulardii*]	119.3	110.61	3.79	1	3	4	5	1056	7.94
KQC45157.1	rRNA-binding protein required for 40S ribosomal subunit biogenesis [*Saccharomyces boulardii*]	25.2	110.55	16.00	1	2	2	3	225	10.08
KQC42997.1	DEAD box RNA helicase [*Saccharomyces boulardii*]	87.1	110.47	6.88	1	4	4	4	770	7.42
KQC44260.1	Vacuolar proteinase B (yscB) with H3 N-terminal endopeptidase activity [*Saccharomyces boulardii*]	69.6	110.23	3.31	1	2	2	3	635	6.46

KQC42454.1	Conserved ER protein translocation channel [*Saccharomyces boulardii*]	52.9	109.33	7.08	1	4	4	4	480	9.35
KQC42984.1	Vacuolar membrane polyphosphate polymerase [*Saccharomyces boulardii*]	38.2	108.99	10.88	1	3	3	3	340	5.60
KQC42804.1	Vesicle membrane protein (v-SNARE) with acyltransferase activity [*Saccharomyces boulardii*]	22.7	108.33	17.50	1	3	3	4	200	5.69
KQC45458.1	Multidrug transporter of the major facilitator superfamily [*Saccharomyces boulardii*]	181.6	107.89	2.42	2	3	3	4	1613	8.65
KQC42722.1	hypothetical protein AB282_03150 [*Saccharomyces boulardii*]	67.6	107.66	3.75	1	2	2	2	587	5.57
KOH50683.1	LEU1p Isopropylmalate isomerase [*Saccharomyces boulardii*]	85.8	107.58	4.11	2	4	4	5	779	5.90
KQC40633.1	Constituent of 66S pre-ribosomal particle [*Saccharomyces boulardii*]	26.4	107.45	12.65	1	2	2	2	245	4.64
KQC43475.1	Subunit of SAGA and NuA4 histone acetyltransferase complex [*Saccharomyces boulardii*]	432.9	107.38	1.10	1	3	4	4	3744	6.57
KQC40913.1	Calcium-binding component of the spindle pole body (SPB) half-bridge [*Saccharomyces boulardii*]	18.7	107.06	26.71	1	3	3	3	161	4.56
KOH51392.1	GET3p Guanine nucleotide exchange factor for Gpa1p [*Saccharomyces boulardii*]	39.3	106.98	10.73	2	3	3	3	354	5.03
KQC41736.1	AMP deaminase [*Saccharomyces boulardii*]	93.2	106.88	6.42	1	4	4	5	810	6.61
KQC42516.1	Peptidyl-prolyl cis-trans isomerase (PPIase) [*Saccharomyces boulardii*]	43.9	106.76	10.20	1	1	3	3	392	4.65
KQC40640.1	hypothetical protein AB282_05275 [*Saccharomyces boulardii*]	45.1	106.48	9.98	1	3	3	3	401	8.31
KQC42744.1	Phosphoglucomutase, minor isoform [*Saccharomyces boulardii*]	63.0	106.36	3.68	2	2	2	3	570	7.03
KQC42983.1	Subunit of vacuolar transporter chaperone (VTC) complex [*Saccharomyces boulardii*]	41.3	106.18	3.14	1	1	1	2	350	7.33
KQC41933.1	Plasma membrane t-SNARE [*Saccharomyces boulardii*]	33.7	106.16	9.15	1	1	2	2	295	4.91
KQC45645.1	3-deoxy-D-arabino-heptulosonate-7-phosphate (DAHP) synthase [*Saccharomyces boulardii*]	39.7	106.12	6.76	1	2	2	2	370	6.95
KQC44802.1	Mitochondrial ribosomal protein of the small subunit [*Saccharomyces boulardii*]	37.4	105.94	12.54	1	3	3	3	319	9.23
KQC44361.1	Fe(II)/2-oxoglutarate-dependent dioxygenase family member [*Saccharomyces boulardii*]	74.0	105.88	4.50	1	2	2	2	644	5.43
KQC41953.1	Component of the TOM (translocase of outer membrane) complex [*Saccharomyces boulardii*]	42.0	105.76	6.98	1	2	2	2	387	5.54
KQC40948.1	Subunit of heteropentameric Replication factor C (RF-C) [*Saccharomyces boulardii*]	94.8	105.47	3.83	1	3	3	3	861	9.22
KQC44661.1	Karyopherin [*Saccharomyces boulardii*]	142.1	105.47	1.47	1	1	1	1	1224	5.35
KQC40441.1	hypothetical protein AB282_05075 [*Saccharomyces boulardii*]	26.7	105.23	10.42	1	3	3	3	240	6.79
KQC40566.1	Essential component of the TIM23 complex [*Saccharomyces boulardii*]	55.1	105.14	5.67	7	2	3	3	476	7.24
KOH52314.1	ECM33p GPI-anchored protein [*Saccharomyces boulardii*]	43.8	105.03	5.83	2	2	2	2	429	4.98
KQC43008.1	Flavoprotein subunit of succinate dehydrogenase [*Saccharomyces boulardii*]	66.0	104.79	8.32	2	2	4	4	601	7.36
KQC43961.1	Nucleotide exchange factor for Gsp1p [*Saccharomyces boulardii*]	53.0	104.78	7.68	1	4	4	4	482	6.10
KQC41420.1	Component of the SSU processome [*Saccharomyces boulardii*]	33.5	104.50	11.03	1	3	3	3	290	9.06
KQC43295.1	Homo-isocitrate dehydrogenase [*Saccharomyces boulardii*]	40.0	104.14	9.43	1	3	3	3	371	8.02

KQC42462.1	Cytoplasmic 60S subunit biogenesis factor [*Saccharomyces boulardii*]	49.7	103.80	12.96	1	3	3	3	432	7.66
KQC43379.1	hypothetical protein AB282_02375 [*Saccharomyces boulardii*]	34.1	103.71	7.74	1	2	2	3	310	9.06
KQC42310.1	Structural component of 3 distinct complex [*Saccharomyces boulardii*]	33.0	103.65	13.47	1	5	5	5	297	5.73
KQC41272.1	Peroxisomal protein required for medium-chain fatty acid oxidation [*Saccharomyces boulardii*]	26.9	103.51	14.83	1	3	3	4	236	9.83
KQC44992.1	putative ATP-dependent RNA helicase of the DEAD-box protein family [*Saccharomyces boulardii*]	112.9	103.18	2.71	1	3	3	3	995	9.09
KQC42117.1	PUF3p of the mitochondrial outer surface [*Saccharomyces boulardii*]	98.0	103.12	5.01	1	4	4	4	879	7.08
KQC45492.1	Subunit of the SAGA transcriptional regulatory complex [*Saccharomyces boulardii*]	152.4	102.92	1.43	7	1	2	2	1332	4.87
KOH48186.1	SIN4p Subunit of the RNA polymerase II mediator complex [*Saccharomyces boulardii*]	284.6	102.77	1.94	3	4	4	4	2527	6.37
KOH52648.1	OSH2p Member of an oxysterol-binding protein family with seven members [*Saccharomyces boulardii*]	126.7	102.07	3.57	3	3	3	3	1121	6.65
KQC42704.1	Subunit C of the V1 peripheral membrane domain of V-ATPase [*Saccharomyces boulardii*]	44.2	101.93	6.38	1	2	2	2	392	6.67
KQC41228.1	Cytoplasmic tryptophanyl-tRNA synthetase [*Saccharomyces boulardii*]	49.3	101.91	10.42	1	4	4	4	432	6.81
KQC45546.1	serine type carboxypeptidase [*Saccharomyces boulardii*]	57.7	101.90	6.30	1	3	3	4	508	5.45
KQC44422.1	RNA export factor associated with the nuclear pore complex (NPC) [*Saccharomyces boulardii*]	40.5	101.85	3.29	1	1	1	2	365	8.48
KQC44314.1	hypothetical protein AB282_01570 [*Saccharomyces boulardii*]	25.1	101.83	18.61	1	4	4	4	231	9.25
KQC43333.1	hypothetical protein AB282_01410 [*Saccharomyces boulardii*]	107.6	101.60	3.07	1	2	2	2	946	6.48
KQC45755.1	Translation elongation factor 1 beta [*Saccharomyces boulardii*]	22.7	101.35	17.39	1	2	2	2	207	4.42
KQC43205.1	Cytosolic J-domain-containing protein [*Saccharomyces boulardii*]	48.5	100.94	8.33	1	2	2	3	432	5.16
KQC45792.1	hypothetical protein AB282_05488 [*Saccharomyces boulardii*]	21.0	100.92	11.40	1	2	2	3	193	6.95
KQC44295.1	putative indolepyruvate decarboxylase family protein [*Saccharomyces boulardii*]	61.2	100.77	4.82	1	2	2	2	560	7.66
KQC43235.1	RNA polymerase II third largest subunit B44 [*Saccharomyces boulardii*]	35.3	100.62	5.66	1	1	1	3	318	4.67
KOH51395.1	RPN6p, non-ATPase regulatory subunit of the 26S proteasome lid [*Saccharomyces boulardii*]	49.7	100.30	6.22	2	2	2	2	434	6.28
KQC42455.1	Phosphatidylinositol transfer protein [*Saccharomyces boulardii*]	47.4	99.96	3.43	1	1	1	2	408	6.62
KQC43593.1	hypothetical protein AB282_02589 [*Saccharomyces boulardii*]	53.0	99.77	5.59	1	2	2	2	465	7.55
KQC41406.1	Nuclear actin-related protein involved in chromatin remodeling [*Saccharomyces boulardii*]	87.4	99.39	4.37	1	2	2	2	755	5.59
KQC40857.1	N-acetylglucosaminyltransferase [*Saccharomyces boulardii*]	57.5	99.11	6.72	1	3	3	3	491	8.37
KQC40985.1	Broad-specificity lysophospholipid acyltransferase [*Saccharomyces boulardii*]	72.2	98.90	5.33	2	2	3	4	619	9.48
KQC42137.1	Essential protein required for ribosomal large subunit biogenesis [*Saccharomyces boulardii*]	21.8	98.79	10.00	1	2	2	2	180	9.74
KQC41067.1	hypothetical protein AB282_04509 [*Saccharomyces boulardii*]	43.7	98.57	3.62	1	1	1	1	387	8.18
KOH49430.1	RSC1p Component of the RSC chromatin remodeling complex [*Saccharomyces boulardii*]	72.2	98.48	3.68	2	2	2	3	625	5.24
KQC44895.1	Polyamine acetyltransferase [*Saccharomyces boulardii*]	21.9	98.21	11.52	1	2	2	2	191	5.82

KQC45073.1	Ubiquitin-specific protease [*Saccharomyces boulardii*]	84.9	98.15	6.59	1	4	4	4	743	4.83
KQC44483.1	Methylglyoxalase that converts methylglyoxal to D-lactate [*Saccharomyces boulardii*]	25.6	98.01	10.55	1	2	2	2	237	5.50
KQC41046.1	Polyphosphatidylinositol phosphatase [*Saccharomyces boulardii*]	124.5	97.29	2.17	1	2	2	3	1107	7.20
KQC42897.1	hypothetical protein AB282_02682 [*Saccharomyces boulardii*]	33.9	97.22	14.73	1	3	3	3	292	5.91
KQC40454.1	Component of the Rpd3L histone deacetylase complex [*Saccharomyces boulardii*]	57.0	97.13	2.57	1	1	1	2	506	4.92
KQC40909.1	Essential non-ATPase regulatory subunit of the 26S proteasome [*Saccharomyces boulardii*]	38.3	97.09	7.99	1	3	3	3	338	5.64
KQC45715.1	Subunit of holo-CPF [*Saccharomyces boulardii*]	88.4	96.95	3.18	1	2	2	2	785	6.15
KQC45074.1	hypothetical protein AB282_01157 [*Saccharomyces boulardii*]	35.6	96.76	4.49	1	1	1	1	312	5.95
KQC41472.1	hypothetical protein AB282_04333 [*Saccharomyces boulardii*]	41.1	96.74	5.59	1	2	2	2	376	6.21
KQC41428.1	EH domain-containing protein involved in endocytosis [*Saccharomyces boulardii*]	40.3	96.59	6.30	1	2	2	2	349	5.47
KQC40942.1	RNA polymerase subunit ABC14.5 [*Saccharomyces boulardii*]	16.5	96.49	10.96	1	1	1	1	146	4.70
KQC44881.1	Integral membrane protein [*Saccharomyces boulardii*]	23.1	96.41	12.06	1	2	2	2	199	8.68
KQC45121.1	RNA-binding subunit of the TRAMP nuclear RNA surveillance complex [*Saccharomyces boulardii*]	33.6	96.20	5.78	2	1	1	1	294	8.56
KQC44753.1	Mitochondrial inner membrane protein [*Saccharomyces boulardii*]	23.6	96.06	9.76	1	2	2	2	205	9.23
KQC45253.1	Negative regulator of the H(+)-ATPase Pma1p [*Saccharomyces boulardii*]	37.0	95.97	11.45	2	3	3	3	332	5.21
KQC41563.1	translation initiation factor eIF1 [*Saccharomyces boulardii*]	12.3	95.88	27.78	1	2	2	2	108	7.97
KQC40987.1	m(7)GpppX pyrophosphatase regulator [*Saccharomyces boulardii*]	41.0	95.65	5.93	1	2	2	3	354	6.64
KQC41967.1	GMP synthase [*Saccharomyces boulardii*]	58.4	94.94	7.24	1	4	4	4	525	6.49
KQC42691.1	Nucleoside diphosphate kinase [*Saccharomyces boulardii*]	17.2	94.40	18.95	1	2	2	2	153	8.60
KQC42688.1	hypothetical protein AB282_03116 [*Saccharomyces boulardii*]	19.0	94.40	11.38	2	2	2	3	167	9.39
KQC40426.1	Mitochondrial protein required for assembly of cytochrome bc1 complex [*Saccharomyces boulardii*]	39.1	94.05	7.46	1	2	2	2	335	9.35
KQC45752.1	Mitochondrial protein [*Saccharomyces boulardii*]	22.1	93.94	9.60	1	1	1	1	198	10.37
KQC40489.1	oxysterol binding protein family [*Saccharomyces boulardii*]	49.4	93.91	6.91	1	3	3	3	434	6.02
KQC41519.1	Chitin synthase I [*Saccharomyces boulardii*]	167.2	93.72	1.43	2	2	2	3	1466	6.35
KQC45315.1	Nucleolar protein required for rRNA synthesis and ribosomal assembly [*Saccharomyces boulardii*]	37.1	93.59	10.13	1	3	3	3	316	9.39
KQC44907.1	hypothetical protein AB282_00973 [*Saccharomyces boulardii*]	131.9	93.55	2.24	2	2	2	2	1204	6.32
KQC41163.1	Mitochondrial protein [*Saccharomyces boulardii*]	65.0	93.54	4.54	1	2	2	2	573	7.77
KOH47373.1	AIM45p protein [*Saccharomyces boulardii*]	36.8	93.18	9.30	4	2	3	3	344	8.51
KQC41580.1	Integral membrane protein [*Saccharomyces boulardii*]	35.5	93.08	4.46	1	1	1	1	314	8.87
KOH47277.1	ELP4p Subunit of hexameric RecA-like ATPase Elp456 Elongator subcomplex [*Saccharomyces boulardii*]	51.1	93.07	5.26	2	2	2	2	456	9.45
KQC43496.1	NMD2p involved in the nonsense-mediated mRNA decay (NMD) pathway [*Saccharomyces boulardii*]	126.5	93.04	3.12	2	1	3	3	1089	5.11
KQC43853.1	Highly-conserved N-terminally anchored AAA-ATPase [*Saccharomyces boulardii*]	40.3	92.99	8.01	1	2	2	2	362	5.72

KQC40436.1	HRR25p kinase [*Saccharomyces boulardii*]	57.3	92.62	4.66	1	3	3	3	494	9.26
KQC41265.1	Acetylornithine aminotransferase [*Saccharomyces boulardii*]	46.7	91.85	2.60	1	1	1	2	423	7.78
KQC43565.1	Mitochondrial manganese superoxide dismutase [*Saccharomyces boulardii*]	25.8	91.84	9.87	1	2	2	2	233	8.48
KQC45217.1	Peptidyl-prolyl cis-trans isomerase (cyclophilin) [*Saccharomyces boulardii*]	35.7	91.63	12.26	1	3	3	4	318	7.09
KQC44145.1	hypothetical protein AB282_01809 [*Saccharomyces boulardii*]	90.9	91.56	2.92	8	1	3	3	787	7.61
KQC43721.1	Squalene epoxidase [*Saccharomyces boulardii*]	55.1	91.50	6.65	1	3	3	3	496	6.48
KQC43874.1	TFIIF (Transcription Factor II) middle subunit [*Saccharomyces boulardii*]	46.6	91.31	5.50	1	2	2	2	400	7.17
KQC45630.1	Subunit of the ARP2/3 complex [*Saccharomyces boulardii*]	42.4	91.25	5.99	1	2	2	2	384	7.05
KQC41065.1	hypothetical protein AB282_04995 [*Saccharomyces boulardii*]	133.4	91.01	4.30	1	3	3	3	1186	7.31
KQC41509.1	Non-essential small GTPase of the Rho/Rac family of Ras-like protein [*Saccharomyces boulardii*]	36.8	90.94	4.23	1	1	1	1	331	8.48
KQC42289.1	Transcription cofactor [*Saccharomyces boulardii*]	90.5	90.82	4.36	1	2	2	2	803	4.88
KOH52297.1	PBY1p putative tubulin tyrosine ligase associated with P-bodies [*Saccharomyces boulardii*]	52.7	90.81	3.74	2	1	1	1	454	4.75
KQC44731.1	Component of GID Complex that confers ubiquitin ligase (U3) activity [*Saccharomyces boulardii*]	49.1	90.77	4.51	1	1	2	3	421	8.24
KQC43370.1	Mitochondrial branched-chain amino acid (BCAA) aminotransferase [*Saccharomyces boulardii*]	43.6	90.62	7.12	2	2	2	2	393	8.91
KQC43261.1	Ribosomal 60S subunit protein L34B [*Saccharomyces boulardii*]	13.6	90.47	26.45	2	3	3	4	121	10.83
KQC44695.1	SSU and 90S preribosome component [*Saccharomyces boulardii*]	62.2	90.34	6.45	1	3	3	3	543	4.68
KQC44281.1	Subunit of the alpha-1,6 mannosyltransferase complex [*Saccharomyces boulardii*]	58.0	89.75	7.01	1	3	3	3	499	5.33
KQC41187.1	Trifunctional enzyme of thiamine biosynthesis, degradation and salvage [*Saccharomyces boulardii*]	61.2	89.58	3.81	1	2	2	2	551	6.16
KQC41053.1	Prephenate dehydrogenase involved in tyrosine biosynthesis [*Saccharomyces boulardii*]	46.0	89.58	8.14	1	3	3	3	393	6.62
KQC45568.1	Covalently-bound cell wall protein [*Saccharomyces boulardii*]	47.9	89.50	5.27	1	2	2	2	455	4.69
KOH50951.1	LOC1p Nuclear protein involved in asymmetric localization of ASH1 mRNA [*Saccharomyces boulardii*]	23.6	89.41	9.80	2	2	2	2	204	10.26
KQC40989.1	Sphingoid long-chain base kinase [*Saccharomyces boulardii*]	69.7	89.40	3.04	1	1	1	1	624	6.44
KQC41803.1	Copper chaperone for superoxide dismutase Sod1p [*Saccharomyces boulardii*]	27.3	89.00	5.22	1	1	1	2	249	6.67
KQC42202.1	hypothetical protein AB282_03382 [*Saccharomyces boulardii*]	50.9	88.73	9.46	1	3	3	3	444	9.17
KQC44708.1	Diacylglycerol pyrophosphate (DGPP) phosphatase [*Saccharomyces boulardii*]	33.5	88.49	22.15	1	5	5	5	289	6.44
KQC43245.1	Alpha catalytic subunit of casein kinase 2 (CK2) [*Saccharomyces boulardii*]	44.6	88.45	7.80	1	3	3	4	372	8.59
KQC41649.1	hypothetical protein AB282_03712 [*Saccharomyces boulardii*]	40.0	88.40	6.30	4	1	2	3	365	8.13
KQC41814.1	Subunit a of the vacuolar-ATPase V0 domain [*Saccharomyces boulardii*]	101.6	87.92	4.16	1	3	3	3	890	5.38
KQC43727.1	hypothetical protein AB282_01983 [*Saccharomyces boulardii*]	10.5	87.27	14.13	1	1	1	2	92	9.99
KQC43926.1	Delta(9) fatty acid desaturase [*Saccharomyces boulardii*]	58.4	87.15	6.27	1	2	2	2	510	9.01

KQC41092.1	NOB1p involved in proteasomal and 40S ribosomal subunit biogenesis [*Saccharomyces boulardii*]	51.7	87.02	5.01	1	2	2	3	459	8.46
KQC43240.1	Subunit of a kinetochore-microtubule binding complex [*Saccharomyces boulardii*]	15.9	86.82	9.22	1	1	1	1	141	8.12
KQC44043.1	Subunit VIa of cytochrome c oxidase [*Saccharomyces boulardii*]	15.0	86.77	21.71	1	3	3	4	129	8.18
KQC41837.1	ATP-dependent RNA helicase of the SFI superfamily [*Saccharomyces boulardii*]	109.4	86.67	2.27	1	1	1	1	971	6.48
KQC45331.1	ECM21p involved in regulating endocytosis of plasma membrane protein [*Saccharomyces boulardii*]	123.5	86.63	3.22	1	3	3	3	1117	6.89
KQC45373.1	AAA ATPase and SNARE disassembly chaperone [*Saccharomyces boulardii*]	32.0	86.44	7.34	1	2	2	2	286	5.02
KQC41219.1	Member of the protein disulfide isomerase (PDI) family [*Saccharomyces boulardii*]	32.3	86.39	11.19	1	2	3	3	277	6.33
KQC41101.1	Ubiquitin-binding protein [*Saccharomyces boulardii*]	46.8	85.81	5.35	1	2	2	2	411	4.78
KQC44140.1	hypothetical protein AB282_01804 [*Saccharomyces boulardii*]	41.1	85.56	4.41	1	2	2	2	363	5.27
KQC44309.1	Beta subunit of the oligosaccharyl transferase glycoprotein complex [*Saccharomyces boulardii*]	49.4	85.44	6.05	1	2	2	2	430	5.90
KQC42405.1	hypothetical protein AB282_03587 [*Saccharomyces boulardii*]	59.4	85.16	2.87	1	1	1	1	523	5.68
KQC42978.1	Adenylate cyclase [*Saccharomyces boulardii*]	228.7	85.01	1.18	3	2	3	3	2034	7.55
KQC43561.1	Target membrane receptor (t-SNARE) [*Saccharomyces boulardii*]	31.0	84.92	9.22	1	2	2	3	282	4.65
KQC43789.1	Cytoplasmic GTPase-activating protein [*Saccharomyces boulardii*]	109.2	84.38	2.53	1	2	2	2	950	5.39
KQC41673.1	Subunit of the signal recognition particle (SRP) [*Saccharomyces boulardii*]	31.2	84.22	16.12	1	4	4	4	273	9.07
KQC44332.1	Nucleotide binding alpha subunit of the heterotrimeric G protein [*Saccharomyces boulardii*]	50.3	84.14	4.01	1	1	1	1	449	6.71
KQC43260.1	Mitochondrial protein required for transamination of isoleucine [*Saccharomyces boulardii*]	15.9	84.10	10.34	1	1	1	1	145	9.28
KQC45176.1	hypothetical protein AB282_01269 [*Saccharomyces boulardii*]	44.3	84.09	3.59	1	1	1	2	390	5.71
KQC43897.1	Subunit of the GET complex [*Saccharomyces boulardii*]	27.1	83.79	11.06	1	2	2	2	235	9.16
KQC40570.1	Plasma membrane ATP-binding cassette (ABC) transporter [*Saccharomyces boulardii*]	170.9	83.75	2.05	1	3	3	3	1511	6.87
KQC42169.1	Microtubule-associated protein (MAP) of the XMAP215/Dis1 family [*Saccharomyces boulardii*]	100.9	83.71	3.38	1	2	2	2	888	8.57
KQC41985.1	GTPase activating protein (GAP) for Gsp1p [*Saccharomyces boulardii*]	45.8	83.64	4.42	1	1	1	1	407	4.67
KQC41544.1	Adenylosuccinate synthase [*Saccharomyces boulardii*]	48.2	83.23	4.39	1	2	2	2	433	8.35
KQC40917.1	Subunit of protein N-terminal acetyltransferase NatA [*Saccharomyces boulardii*]	19.7	82.98	17.61	1	2	2	2	176	7.91
KQC43258.1	putative aminophospholipid translocase (flippase) [*Saccharomyces boulardii*]	130.1	82.62	0.96	1	1	1	2	1151	6.47
KQC41514.1	Ubiquitin-specific protease involved in transport and osmotic response [*Saccharomyces boulardii*]	88.1	82.07	1.78	1	1	1	1	788	5.58
KQC45518.1	AIM3p that inhibits barbed-end actin filament elongation [*Saccharomyces boulardii*]	103.4	81.86	1.38	1	1	1	2	942	9.25
KQC45716.1	ERV29p [*Saccharomyces boulardii*]	46.2	81.82	5.78	1	2	2	2	415	6.83
KQC42907.1	Alpha-1,6-mannosyltransferase [*Saccharomyces boulardii*]	46.3	81.74	4.55	1	2	2	2	396	8.10

KQC42132.1	putative plasma membrane permease [*Saccharomyces boulardii*]	91.6	81.66	2.58	1	2	2	2	813	8.25
KQC43899.1	Subunit of the oligosaccharyltransferase complex of the ER lumen [*Saccharomyces boulardii*]	81.4	81.33	4.60	1	3	3	3	718	8.18
KQC43399.1	Conserved NADPH oxidoreductase containing flavin mononucleotide (FMN) [*Saccharomyces boulardii*]	45.0	81.31	2.25	1	1	1	2	400	6.39
KQC40994.1	GET4p [*Saccharomyces boulardii*]	36.2	81.25	8.33	1	2	2	2	312	5.20
KQC42913.1	Subunit of heteropentameric Replication factor C (RF-C) [*Saccharomyces boulardii*]	39.8	81.13	6.80	1	2	2	2	353	8.59
KQC45040.1	FG-nucleoporin component of central core of nuclear pore complex (NPC) [*Saccharomyces boulardii*]	58.8	80.90	3.41	1	1	1	1	528	7.68
KQC44981.1	ERP3p with similarity to Emp24p and Erv25p [*Saccharomyces boulardii*]	20.7	80.78	9.66	1	1	1	1	176	7.47
KQC43916.1	Aminolevulinate dehydratase [*Saccharomyces boulardii*]	37.7	80.69	5.56	1	1	1	1	342	6.14
KQC41833.1	Cohesion maintenance factor [*Saccharomyces boulardii*]	142.3	80.61	2.10	2	2	2	2	1237	6.39
AGT20490.1	alkaline phosphatase [*Saccharomyces boulardii*]	63.0	80.50	2.65	1	1	1	3	566	5.59
KQC41976.1	NADP(+)-dependent serine dehydrogenase and carbonyl reductase [*Saccharomyces boulardii*]	29.2	80.44	5.24	1	1	1	1	267	6.81
KQC45699.1	NADP(+)-dependent glutamate dehydrogenase [*Saccharomyces boulardii*]	49.6	79.58	8.97	1	1	4	4	457	5.47
KOH50954.1	RPN11p Metalloprotease subunit of 19S regulatory particle [*Saccharomyces boulardii*]	28.5	79.42	11.02	2	3	3	3	254	6.65
KQC44963.1	Ran GTPase binding protein [*Saccharomyces boulardii*]	23.9	79.20	5.26	1	1	1	2	209	6.43
KQC44152.1	hypothetical protein AB282_01816 [*Saccharomyces boulardii*]	55.4	79.13	3.73	1	1	1	1	482	5.88
KQC40764.1	hypothetical protein AB282_05399 [*Saccharomyces boulardii*]	22.5	79.12	12.50	9	2	3	3	200	6.55
KQC41104.1	Subunit of HIR nucleosome assembly complex [*Saccharomyces boulardii*]	98.4	78.90	3.20	1	2	2	2	875	8.46
KQC42923.1	putative serine/threonine protein kinase [*Saccharomyces boulardii*]	91.4	78.71	4.40	1	3	3	3	818	8.97
KQC43517.1	Component of the RSC chromatin remodeling complex [*Saccharomyces boulardii*]	101.4	78.67	2.49	3	2	3	3	883	8.03
KQC43043.1	Nuclear actin-related protein involved in chromatin remodeling [*Saccharomyces boulardii*]	54.8	78.63	5.93	1	3	3	3	489	5.45
KQC41119.1	Mitochondrial matrix co-chaperonin [*Saccharomyces boulardii*]	11.4	78.30	19.81	1	2	2	2	106	9.00
KQC43503.1	tRNA(m(1)G37)methyltransferase [*Saccharomyces boulardii*]	54.3	78.29	3.13	1	1	1	1	480	8.54
KOH49746.1	IML2p protein [*Saccharomyces boulardii*]	82.5	78.07	3.15	2	1	3	3	731	6.64
KQC42542.1	Integral membrane peptide transporter [*Saccharomyces boulardii*]	67.9	77.70	6.32	1	3	3	3	601	5.39
KQC43189.1	18kDa catalytic subunit of the Signal Peptidase Complex (SPC) [*Saccharomyces boulardii*]	18.7	77.69	14.37	1	2	2	2	167	9.16
KQC43253.1	Microsomal cytochrome b reductase [*Saccharomyces boulardii*]	31.4	77.65	9.86	1	3	3	3	284	8.78
KQC44460.1	Coiled-coil 12S polarisome subunit [*Saccharomyces boulardii*]	48.2	77.58	2.38	1	1	1	2	420	5.59
KQC43986.1	Major isozyme of methylenetetrahydrofolate reductase [*Saccharomyces boulardii*]	68.5	77.55	4.50	1	2	2	2	600	5.80
KQC42354.1	Rab family GTPase [*Saccharomyces boulardii*]	29.1	77.53	9.27	1	1	2	2	259	8.60
KQC41301.1	putative GTPase [*Saccharomyces boulardii*]	55.5	77.38	4.94	1	2	2	2	486	9.50
KQC40509.1	Vacuolar RNase of the T(2) family [*Saccharomyces boulardii*]	50.1	77.33	6.45	1	2	2	2	434	8.05

KQC42714.1	GTPase activating factor for Rsr1p/Bud1p [*Saccharomyces boulardii*]	126.6	77.30	1.63	5	1	2	2	1104	8.82
KQC45080.1	Ribosomal Protein P1 Beta [*Saccharomyces boulardii*]	10.7	77.14	15.09	1	1	1	1	106	4.01
KQC44814.1	Sm-like protein [*Saccharomyces boulardii*]	83.4	76.99	3.31	1	2	2	2	724	7.30
KQC43110.1	Fructose-2,6-bisphosphatase, required for glucose metabolism [*Saccharomyces boulardii*]	52.6	76.99	3.98	1	2	2	2	452	6.81
KQC44377.1	Conserved nuclear RNA-binding protein [*Saccharomyces boulardii*]	24.1	76.86	9.17	1	2	2	2	218	7.25
KQC42596.1	Essential subunit of the Dam1 complex (aka DASH complex) [*Saccharomyces boulardii*]	34.1	76.85	8.14	1	2	2	3	295	8.29
KQC41971.1	Found in Mitochondrial Proteome [*Saccharomyces boulardii*]	56.2	76.78	2.18	1	1	1	1	504	7.08
KQC43702.1	Cargo-transport protein involved in endocytosis [*Saccharomyces boulardii*]	182.3	75.93	1.78	1	2	2	2	1570	6.16
KOH52414.1	RIB1p GTP cyclohydrolase II [*Saccharomyces boulardii*]	38.3	75.65	5.22	1	1	1	1	345	6.34
KQC42211.1	Alanine transaminase (glutamic pyruvic transaminase) [*Saccharomyces boulardii*]	62.6	75.63	7.71	1	3	3	3	558	6.60
KQC43286.1	Subunit of the CoA-Synthesizing Protein Complex (CoA-SPC) [*Saccharomyces boulardii*]	41.8	75.29	7.95	1	2	2	2	365	8.27
KQC40497.1	Conserved protein of the mitochondrial matrix [*Saccharomyces boulardii*]	17.9	75.13	12.73	1	2	2	2	165	9.58
KQC44649.1	putative GPI-anchored aspartic protease [*Saccharomyces boulardii*]	64.5	75.08	2.85	1	1	1	2	596	4.92
KQC45557.1	hypothetical protein AB282_00319 [*Saccharomyces boulardii*]	35.7	74.90	6.96	1	2	2	2	316	7.18
KQC44125.1	Subunit 6 of the ubiquinol cytochrome-c reductase complex [*Saccharomyces boulardii*]	17.2	74.90	9.52	1	1	1	1	147	4.09
KQC44932.1	ENA5p [*Saccharomyces boulardii*]	120.1	74.90	2.20	1	2	2	2	1091	5.54
KQC43127.1	Subunit of heterotrimeric Replication Protein A (RPA) [*Saccharomyces boulardii*]	13.8	74.86	12.30	1	1	1	1	122	4.79
KQC43282.1	Epsilon-COP subunit of the coatomer [*Saccharomyces boulardii*]	33.8	74.69	8.78	1	2	2	2	296	4.55
KQC43792.1	Component of RNA polymerase II holoenzyme [*Saccharomyces boulardii*]	126.8	74.49	1.92	1	1	1	1	1146	9.29
KQC44225.1	RNA-dependent ATPase RNA helicase (DEIH box) [*Saccharomyces boulardii*]	245.8	74.34	0.65	1	1	2	2	2162	5.53
KQC43628.1	Plasma membrane ATP-binding cassette (ABC) transporter [*Saccharomyces boulardii*]	166.6	74.22	0.95	1	1	1	1	1477	7.68
KQC41223.1	putative vacuolar membrane transporter [*Saccharomyces boulardii*]	34.9	74.08	6.49	1	1	1	1	308	4.92
KQC43382.1	Nucleolar protein [*Saccharomyces boulardii*]	65.2	73.95	4.87	1	2	2	3	575	4.86
KQC43914.1	Mannosyltransferase of the cis-Golgi apparatus [*Saccharomyces boulardii*]	55.1	73.86	2.08	1	1	1	1	480	7.68
KQC42322.1	Nucleolar protein [*Saccharomyces boulardii*]	91.0	73.81	1.59	1	1	1	1	817	5.43
KQC40577.1	Subunit of Golgi mannosyltransferase complex [*Saccharomyces boulardii*]	37.6	73.69	6.13	1	2	2	2	326	6.09
KQC45219.1	Integral ER membrane protein that stimulates Sar1p GTPase activity [*Saccharomyces boulardii*]	114.0	73.65	1.60	1	1	2	2	1065	4.73
KQC43361.1	hypothetical protein AB282_01438 [*Saccharomyces boulardii*]	85.0	73.55	0.93	4	1	1	2	864	4.45
KQC41259.1	RING-H2 domain core subunit of multiple ubiquitin ligase complex [*Saccharomyces boulardii*]	13.9	73.42	11.57	1	1	1	1	121	5.81
KQC44313.1	Mannose-6-phosphate isomerase [*Saccharomyces boulardii*]	48.2	73.40	4.20	1	2	2	2	429	5.99

KQC42909.1	Methylene-fatty-acyl-phospholipid synthase [*Saccharomyces boulardii*]	23.1	73.38	13.59	1	2	2	2	206	8.98
KQC42099.1	putative ATPase of the AAA family [*Saccharomyces boulardii*]	93.0	73.33	2.03	4	2	3	4	837	5.48
KQC41066.1	Gamma subunit of the oligosaccharyltransferase complex of the ER lumen [*Saccharomyces boulardii*]	39.4	73.32	5.43	1	2	2	2	350	8.48
KQC43669.1	SPG1p required for high temperature survival during stationary phase [*Saccharomyces boulardii*]	10.5	73.17	11.58	1	1	1	1	95	8.46
KQC45837.1	Arginase, catabolizes arginine to ornithine and urea [*Saccharomyces boulardii*]	35.6	73.01	4.80	1	2	2	2	333	5.64
KQC45758.1	FG-nucleoporin component of central core of the nuclear pore complex [*Saccharomyces boulardii*]	56.4	72.96	5.68	1	2	2	2	511	9.41
KQC41035.1	Glycerol dehydrogenase [*Saccharomyces boulardii*]	35.1	72.93	7.69	1	2	2	2	312	7.99
KQC42643.1	Subunit of the ARP2/3 complex [*Saccharomyces boulardii*]	19.9	72.84	9.36	1	1	1	1	171	5.40
KQC43501.1	Lanosterol synthase [*Saccharomyces boulardii*]	83.4	72.84	3.01	2	2	2	2	731	6.61
KQC40549.1	Essential protein involved in regulation of transcription [*Saccharomyces boulardii*]	209.8	72.81	1.02	1	2	2	2	1867	6.57
KOH52291.1	EXO84p Exocyst subunit with dual roles in exocytosis and spliceosome assembly [*Saccharomyces boulardii*]	85.5	72.67	1.46	2	1	1	1	753	7.94
KQC41024.1	Subunit of the membrane-associated retromer complex [*Saccharomyces boulardii*]	63.0	72.66	3.81	1	3	3	3	551	7.58
KQC40935.1	Mitochondrial matrix cochaperone [*Saccharomyces boulardii*]	26.1	72.41	9.65	1	2	2	2	228	8.24
KQC44185.1	Alanine:glyoxylate aminotransferase (AGT) [*Saccharomyces boulardii*]	41.9	72.35	7.01	1	2	2	3	385	8.00
KQC41939.1	P subunit of the mitochondrial glycine decarboxylase complex [*Saccharomyces boulardii*]	114.4	71.86	0.97	1	1	1	2	1034	7.25
KQC40429.1	tRNA:pseudouridine synthase [*Saccharomyces boulardii*]	62.1	71.85	3.68	1	1	2	3	544	8.13
KQC41776.1	High-affinity glucose transporter of the major facilitator superfamily [*Saccharomyces boulardii*]	59.8	71.57	3.14	4	1	2	2	541	8.32
KQC44312.1	Constituent of 66S pre-ribosomal particle [*Saccharomyces boulardii*]	27.0	71.56	5.19	1	1	1	1	231	9.17
KQC45296.1	Cytoplasmic RNA-binding protein [*Saccharomyces boulardii*]	51.8	71.55	7.08	1	2	2	2	466	8.72
KQC42843.1	hypothetical protein AB282_02628 [*Saccharomyces boulardii*]	30.1	71.53	8.55	1	2	2	2	269	9.32
KQC44416.1	Lipid raft associated protein [*Saccharomyces boulardii*]	48.3	71.49	4.19	1	2	2	2	430	8.95
KQC42223.1	3-keto sterol reductase [*Saccharomyces boulardii*]	39.7	71.30	7.49	1	3	3	3	347	8.73
KQC44089.1	Nuclear protein with decapping endonuclease activity [*Saccharomyces boulardii*]	44.5	70.75	6.20	1	2	2	2	387	6.57
KQC44159.1	putative ATP-dependent RNA helicase [*Saccharomyces boulardii*]	69.4	70.28	1.98	1	1	1	1	606	9.04
KQC45570.1	Soluble GTPase with a role in regulation of membrane traffic [*Saccharomyces boulardii*]	20.4	70.12	6.01	1	1	1	1	183	4.92
KQC41050.1	Exomer-dependent cargo protein [*Saccharomyces boulardii*]	32.1	70.10	4.26	1	1	1	1	282	5.77
KQC44589.1	Component of the pre-60S pre-ribosomal particle [*Saccharomyces boulardii*]	28.0	70.03	3.83	1	1	1	1	235	9.38
KQC44439.1	NSA2p constituent of 66S pre-ribosomal particle [*Saccharomyces boulardii*]	29.7	70.00	14.18	1	3	3	4	261	10.24
KQC43373.1	AGC family protein kinase [*Saccharomyces boulardii*]	91.8	69.64	2.43	1	2	2	3	824	6.06
KQC41148.1	Histone variant H2AZ [*Saccharomyces boulardii*]	14.3	69.61	14.18	1	2	2	2	134	10.65

KQC42675.1	hypothetical protein AB282_03103 [*Saccharomyces boulardii*]	103.2	69.57	1.74	1	1	2	2	922	6.10
KQC43764.1	Component of the small-subunit (SSU) processome [*Saccharomyces boulardii*]	80.1	69.53	2.52	1	1	1	1	713	5.17
KQC43324.1	Component of cytosolic iron-sulfur protein assembly (CIA) machinery [*Saccharomyces boulardii*]	117.8	69.52	1.94	1	1	1	1	1032	5.90
KQC45233.1	Mitochondrial ribosomal protein of the large subunit [*Saccharomyces boulardii*]	19.4	69.38	6.51	1	1	1	1	169	10.39
KQC41765.1	Monomeric glyoxalase I [*Saccharomyces boulardii*]	37.2	69.38	8.59	1	2	2	3	326	6.65
KQC43418.1	KEL1p required for proper cell fusion and cell morphology [*Saccharomyces boulardii*]	131.1	69.29	2.66	1	2	2	2	1164	5.35
KQC40396.1	hypothetical protein AB282_05030 [*Saccharomyces boulardii*]	29.5	69.14	6.11	1	2	2	2	262	7.49
KQC43430.1	CRP1p [*Saccharomyces boulardii*]	51.1	69.01	4.95	1	2	2	2	465	7.34
KQC43739.1	Essential component of CPF (cleavage and polyadenylation factor) [*Saccharomyces boulardii*]	47.0	68.97	2.35	1	1	1	1	425	6.27
KQC43306.1	6-phosphofructo-2-kinase [*Saccharomyces boulardii*]	93.5	68.89	3.02	1	2	2	2	827	8.46
KQC44007.1	Bifunctional chorismate synthase and flavin reductase [*Saccharomyces boulardii*]	40.8	68.51	5.85	1	2	2	2	376	7.80
KQC42300.1	N-myristoyl transferase [*Saccharomyces boulardii*]	52.7	68.46	2.42	1	1	1	1	455	7.69
KQC44914.1	Component of serine palmitoyltransferase [*Saccharomyces boulardii*]	63.1	68.26	3.03	1	2	2	2	561	8.10
KQC41543.1	Mannosyltransferase, involved in N-linked glycosylation [*Saccharomyces boulardii*]	51.6	68.17	2.65	1	1	1	1	453	8.98
KQC40745.1	Peroxisomal adenine nucleotide transporter [*Saccharomyces boulardii*]	36.3	68.16	3.35	1	1	1	1	328	9.77
KOH47943.1	RTS1p B-type regulatory subunit of protein phosphatase 2A (PP2A) [*Saccharomyces boulardii*]	85.3	68.04	2.91	2	2	2	3	757	6.32
KQC41061.1	hypothetical protein AB282_04991 [*Saccharomyces boulardii*]	39.5	68.04	3.77	1	1	1	1	345	9.33
KQC45327.1	PKC1p serine/threonine kinase [*Saccharomyces boulardii*]	131.4	67.99	1.82	1	2	2	3	1151	7.11
KQC44462.1	Ubiquitin-specific protease [*Saccharomyces boulardii*]	101.8	67.69	1.64	1	1	1	1	912	7.84
KQC43692.1	Cytoplasmic thioredoxin isoenzyme [*Saccharomyces boulardii*]	11.2	67.60	23.08	1	2	2	2	104	4.93
KQC40583.1	Nucleolar protein [*Saccharomyces boulardii*]	77.7	67.57	2.77	1	2	2	2	685	9.03
KQC44724.1	hypothetical protein AB282_00772 [*Saccharomyces boulardii*]	30.6	67.51	9.56	1	2	2	2	272	5.41
KQC45757.1	Subunit of RNA polymerase III transcription initiation factor complex [*Saccharomyces boulardii*]	132.1	67.40	1.03	1	1	1	2	1160	9.32
KQC44366.1	putative regulatory subunit of protein phosphatase Glc7p [*Saccharomyces boulardii*]	62.1	67.37	2.37	1	1	1	1	548	8.78
KQC44327.1	Microtubule plus end-tracking protein [*Saccharomyces boulardii*]	34.1	67.36	5.25	1	1	1	1	305	5.44
KQC43504.1	Exosome non-catalytic core component [*Saccharomyces boulardii*]	39.4	67.30	3.34	1	1	1	1	359	6.25
KQC44645.1	Cytoplasmic thioredoxin reductase [*Saccharomyces boulardii*]	34.2	67.26	15.36	2	2	2	2	319	5.94
KQC45488.1	hypothetical protein AB282_00250 [*Saccharomyces boulardii*]	18.3	66.95	13.58	1	2	2	2	162	5.05
KQC42931.1	Fumarate reductase, catalyzes the reduction of fumarate to succinate [*Saccharomyces boulardii*]	53.9	66.86	6.21	1	2	2	2	483	9.16
KQC41753.1	Asn rich cytoplasmic protein [*Saccharomyces boulardii*]	63.7	66.74	3.47	1	1	1	1	576	8.40
KQC43408.1	ATPase, putative RNA helicase of the DEAD-box family [*Saccharomyces boulardii*]	47.8	66.43	5.57	1	2	2	2	431	9.61

KQC45838.1	Peripheral peroxisomal membrane peroxin [*Saccharomyces boulardii*]	44.9	66.32	5.58	1	1	2	2	394	9.03
KQC42755.1	Subunit of succinate dehydrogenase and of TIM22 translocase [*Saccharomyces boulardii*]	22.1	66.26	5.56	1	1	1	1	198	10.23
KOH47919.1	WHI2p Protein required for full activation of the general stress response [*Saccharomyces boulardii*]	52.6	66.21	4.35	2	2	2	2	460	6.07
KQC40781.1	RNA polymerase III subunit C82 [*Saccharomyces boulardii*]	74.0	66.20	3.06	1	2	2	2	654	6.67
KQC45707.1	hypothetical protein AB282_00012 [*Saccharomyces boulardii*]	87.4	65.85	2.30	1	2	2	2	783	7.94
KQC42424.1	hypothetical protein AB282_03606 [*Saccharomyces boulardii*]	58.4	65.78	2.55	1	1	1	1	509	6.44
KOH49762.1	MRPL8p Mitochondrial ribosomal protein of the large subunit [*Saccharomyces boulardii*]	26.9	65.67	10.92	2	1	1	2	238	9.73
KQC43806.1	Delta subunit of the translation initiation factor eIF2B [*Saccharomyces boulardii*]	70.8	65.41	4.76	1	2	2	2	651	9.42
KQC43960.1	Homeodomain-containing protein and putative transcription factor [*Saccharomyces boulardii*]	31.2	65.38	3.99	1	1	1	2	276	10.23
KQC44861.1	TMN2p with a role in cellular adhesion and filamentous growth [*Saccharomyces boulardii*]	76.3	65.26	3.13	1	1	2	2	672	7.49
KQC44381.1	hypothetical protein AB282_01637 [*Saccharomyces boulardii*]	19.0	65.11	13.04	1	2	2	2	161	6.18
KQC42698.1	Molecular chaperone of the endoplasmic reticulum lumen [*Saccharomyces boulardii*]	99.6	65.01	2.38	1	2	2	2	881	5.31
KQC45416.1	Nucleolar protein required for export of tRNAs from the nucleus [*Saccharomyces boulardii*]	287.4	64.90	1.24	1	3	4	4	2493	6.96
KQC40498.1	Mitochondrial inner membrane transporter [*Saccharomyces boulardii*]	34.2	64.85	6.77	1	2	2	2	310	9.86
KQC41030.1	Isoamyl acetate-hydrolyzing esterase [*Saccharomyces boulardii*]	27.3	64.79	5.88	1	1	1	1	238	5.50
KQC43545.1	Dipeptidyl aminopeptidase [*Saccharomyces boulardii*]	93.4	64.74	1.83	1	1	1	1	818	5.52
KOH47374.1	HAL1p Cytoplasmic protein involved in halotolerance [*Saccharomyces boulardii*]	33.0	64.51	6.12	2	1	1	1	294	8.79
KQC40702.1	Beta 5 subunit of the 20S proteasome [*Saccharomyces boulardii*]	31.6	64.32	12.89	1	3	3	3	287	6.23
KQC44806.1	Small heat shock protein (sHSP) with chaperone activity [*Saccharomyces boulardii*]	42.8	64.26	4.53	1	2	2	3	375	5.08
KQC42238.1	Component of commitment complex [*Saccharomyces boulardii*]	46.7	64.26	2.88	2	1	1	1	416	9.55
KQC43861.1	Subunit F of the V1 peripheral membrane domain of V-ATPase [*Saccharomyces boulardii*]	13.5	64.18	28.81	1	3	3	3	118	5.05
KQC41620.1	Essential nucleolar protein required for 40S ribosome biogenesis [*Saccharomyces boulardii*]	68.6	63.98	2.37	1	1	1	1	591	5.10
KQC41771.1	TFIID subunit (48 kDa) [*Saccharomyces boulardii*]	42.2	63.91	2.84	1	1	1	1	388	9.66
KQC44078.1	Essential 113 kDa subunit of the exocyst complex [*Saccharomyces boulardii*]	133.0	63.40	1.74	1	2	2	2	1150	5.12
KQC41632.1	Calcium/H+ antiporter localized to the endoplasmic reticulum membrane [*Saccharomyces boulardii*]	102.4	63.31	2.42	1	2	2	2	908	7.15
KOH51147.1	MEI4p Meiosis-specific protein involved in forming DSBs [*Saccharomyces boulardii*]	51.9	63.30	2.48	2	1	1	2	444	9.42
KQC45226.1	Conserved 90S pre-ribosomal component [*Saccharomyces boulardii*]	103.9	63.19	1.08	1	1	1	1	923	5.10
KOH52197.1	FTH1p putative high affinity iron transporter [*Saccharomyces boulardii*]	51.4	63.16	3.87	2	2	2	2	465	5.76
KQC40613.1	Zeta subunit of the coatomer complex (COPI) [*Saccharomyces boulardii*]	21.7	63.13	13.76	1	2	2	2	189	5.16

KQC41052.1	Mitochondrial inner membrane carnitine transporter [*Saccharomyces boulardii*]	34.8	63.10	7.34	1	2	2	2	327	9.88
KQC43834.1	Mitochondrial outer membrane protein [*Saccharomyces boulardii*]	20.1	63.07	6.42	1	2	2	3	187	8.53
KQC41327.1	Peptidyl-prolyl cis-trans isomerase (cyclophilin) [*Saccharomyces boulardii*]	33.4	63.05	8.78	2	1	1	1	296	6.81
KQC43521.1	Subunit VI of cytochrome c oxidase (Complex IV) [*Saccharomyces boulardii*]	17.3	62.83	18.24	1	3	3	3	148	6.05
KQC44108.1	Subunit of the 19S regulatory particle of the 26S proteasome lid [*Saccharomyces boulardii*]	31.9	62.60	5.11	5	1	2	2	274	4.92
KQC44298.1	GTT3p involved in glutathione metabolism [*Saccharomyces boulardii*]	38.2	62.48	6.53	1	1	1	1	337	5.03
KQC45538.1	SHE3p adaptor between Myo4p and the She2p-mRNA complex [*Saccharomyces boulardii*]	47.5	62.41	2.82	1	1	1	1	425	8.87
KOH52645.1	TSC13p Enoyl reductase [*Saccharomyces boulardii*]	36.8	62.40	5.81	2	2	2	2	310	9.54
KQC45725.1	hypothetical protein AB282_00030 [*Saccharomyces boulardii*]	47.0	62.24	2.91	1	1	1	1	413	9.86
KQC44643.1	Inner plaque spindle pole body (SPB) component [*Saccharomyces boulardii*]	111.7	62.15	1.59	1	1	1	1	944	7.08
KQC45009.1	glucose deprivation induced stress granule component [*Saccharomyces boulardii*]	19.8	62.11	7.57	1	1	1	1	185	10.35
KQC42815.1	Indole-3-glycerol-phosphate synthase [*Saccharomyces boulardii*]	53.5	61.83	4.96	1	2	2	2	484	6.92
KQC44852.1	tRNA methyltransferase [*Saccharomyces boulardii*]	62.2	61.47	4.15	1	2	2	2	554	9.09
KQC42305.1	COQ9p required for ubiquinone biosynthesis and respiratory growth [*Saccharomyces boulardii*]	29.9	61.43	5.38	1	1	1	1	260	8.79
KQC40589.1	Subunit beta1 of the nascent polypeptide-associated complex (NAC) [*Saccharomyces boulardii*]	17.0	61.38	12.10	1	2	2	2	157	6.55
KQC40722.1	Polyamine transporter of the major facilitator superfamily [*Saccharomyces boulardii*]	68.1	61.22	2.09	1	1	1	1	622	7.06
KQC41839.1	Mitochondrial alcohol dehydrogenase isozyme III [*Saccharomyces boulardii*]	40.3	61.09	4.80	1	2	2	2	375	8.43
KQC41221.1	MSH2p [*Saccharomyces boulardii*]	108.8	60.88	2.59	1	2	2	2	964	6.15
KQC43715.1	E3 ubiquitin ligase (N-recognin) [*Saccharomyces boulardii*]	224.6	60.84	1.08	1	1	1	1	1950	5.57
KQC43319.1	SIM1p of the SUN family (Sim1p, Uth1p, Nca3p, Sun4p) [*Saccharomyces boulardii*]	48.0	60.82	3.80	1	2	2	2	474	4.60
KQC41451.1	Constituent of 66S pre-ribosomal particle [*Saccharomyces boulardii*]	25.4	60.77	9.55	1	2	2	2	220	9.28
KQC44328.1	Mitochondrial inner membrane m-AAA protease component [*Saccharomyces boulardii*]	84.5	60.67	2.63	2	2	2	2	761	9.16
KQC43738.1	Phosphatidylethanolamine methyltransferase (PEMT) [*Saccharomyces boulardii*]	101.2	60.65	1.73	1	1	2	2	869	8.51
KQC44700.1	Dihydrosphingosine phosphate lyase [*Saccharomyces boulardii*]	65.5	60.43	3.57	1	3	3	3	589	8.03
KQC43552.1	RPS27Bp component of the small (40S) ribosomal subunit [*Saccharomyces boulardii*]	9.9	60.25	16.48	1	2	2	2	91	8.50
KQC42144.1	Methylthioadenosine phosphorylase (MTAP) [*Saccharomyces boulardii*]	35.6	60.16	6.92	1	2	2	2	318	6.60
KQC42577.1	Glycogenin glucosyltransferase [*Saccharomyces boulardii*]	69.7	60.11	3.25	1	2	2	2	616	4.36
KQC43122.1	Subunit 8 of ubiquinol cytochrome-c reductase (Complex III) [*Saccharomyces boulardii*]	11.0	59.95	12.77	1	1	1	1	94	9.70

KQC44740.1	Subunit of a Golgi mannosyltransferase complex [*Saccharomyces boulardii*]	46.7	59.91	7.63	1	3	3	3	393	6.89
KQC42006.1	Translation initiation factor eIF1A [*Saccharomyces boulardii*]	17.5	59.88	7.19	1	1	1	1	153	4.83
KQC45565.1	GPI-anchored aspartyl protease [*Saccharomyces boulardii*]	38.7	59.83	3.17	1	1	1	1	347	9.42
KQC45008.1	1-acyl-sn-glycerol-3-phosphate acyltransferase [*Saccharomyces boulardii*]	33.8	59.83	2.97	1	1	1	1	303	9.54
KQC40488.1	Nucleolar protein [*Saccharomyces boulardii*]	52.7	59.62	2.19	1	1	1	1	456	9.31
KOH49710.1	MTC1p protein [*Saccharomyces boulardii*]	50.5	59.60	2.86	2	1	1	1	454	4.93
KQC43723.1	RBG2p with a role in translation [*Saccharomyces boulardii*]	41.0	59.42	5.43	1	2	2	2	368	8.56
KQC44671.1	Subunit of U3-containing 90S preribosome and SSU processome complex [*Saccharomyces boulardii*]	87.7	59.41	2.32	1	1	2	2	776	6.57
KOH52035.1	CWH43p putative sensor/transporter protein involved in cell wall biogenesis [*Saccharomyces boulardii*]	107.8	59.40	1.26	1	1	1	1	953	6.25
KQC44097.1	High-affinity zinc transporter of the plasma membrane [*Saccharomyces boulardii*]	41.3	59.25	3.48	1	1	1	2	374	5.60
KQC44365.1	Mitochondrial copper and phosphate carrier [*Saccharomyces boulardii*]	33.5	59.24	3.33	1	1	1	1	300	9.39
KQC44318.1	hypothetical protein AB282_01574 [*Saccharomyces boulardii*]	20.2	59.24	11.05	1	2	2	2	181	7.03
KQC43429.1	Mitochondrial ribosomal protein of the large subunit [*Saccharomyces boulardii*]	23.8	59.17	6.54	1	1	1	1	214	9.96
KQC42595.1	ATPase of HSP70/DnaK family [*Saccharomyces boulardii*]	46.6	59.12	3.80	1	1	1	1	421	7.42
KQC43458.1	Mitochondrial outer membrane protein [*Saccharomyces boulardii*]	71.8	58.90	1.56	1	1	1	2	639	6.14
KQC45294.1	Glutathione-dependent disulfide oxidoreductase [*Saccharomyces boulardii*]	12.4	58.78	14.55	1	1	1	1	110	5.06
KQC42862.1	ENT3p containing an N-terminal epsin-like domain [*Saccharomyces boulardii*]	45.1	58.75	5.64	1	2	2	2	408	4.84
KQC42776.1	cAMP-dependent protein kinase catalytic subunit [*Saccharomyces boulardii*]	45.9	58.74	3.02	1	1	1	1	398	6.34
KQC43613.1	Transcription factor involved in iron utilization and homeostasis [*Saccharomyces boulardii*]	70.8	58.59	1.75	1	1	1	1	627	8.34
KQC40617.1	Vacuolar membrane protein [*Saccharomyces boulardii*]	125.7	58.40	2.25	2	2	2	2	1110	5.19
KQC45330.1	Tetra-spanning membrane protein found mostly in the late Golgi [*Saccharomyces boulardii*]	24.3	58.38	5.58	1	1	1	1	215	8.84
KQC43476.1	hypothetical protein AB282_02472 [*Saccharomyces boulardii*]	103.9	58.38	2.26	1	2	2	2	929	6.29
KQC42554.1	hypothetical protein AB282_02982 [*Saccharomyces boulardii*]	39.6	58.36	2.62	1	1	1	1	344	9.09
KQC43499.1	Glutamine-dependent NAD(+) synthetase [*Saccharomyces boulardii*]	80.7	58.36	2.10	1	1	1	1	714	6.54
KQC41583.1	Phosphatidylinositol 4-kinase [*Saccharomyces boulardii*]	119.9	58.15	1.50	1	1	1	1	1066	6.40
KQC42180.1	C-5 sterol desaturase [*Saccharomyces boulardii*]	42.7	57.95	4.66	1	2	2	3	365	7.69
KQC40680.1	Subunit of CCR4-NOT global transcriptional regulator [*Saccharomyces boulardii*]	65.8	57.94	6.07	1	2	2	2	560	5.43
KQC43449.1	hypothetical protein AB282_02445 [*Saccharomyces boulardii*]	27.0	57.43	8.23	1	2	2	2	243	10.54
KQC44008.1	ATPase and nucleosome spacing factor [*Saccharomyces boulardii*]	171.3	57.26	2.35	1	3	3	3	1489	5.78
KQC41389.1	hypothetical protein AB282_04250 [*Saccharomyces boulardii*]	50.9	57.25	2.85	1	1	1	1	456	6.55
KQC44353.1	Methionine-S-sulfoxide reductase [*Saccharomyces boulardii*]	18.3	57.24	5.00	1	1	1	2	160	7.39

KQC43244.1	Beta subunit of the capping protein heterodimer (Cap1p and Cap2p) [*Saccharomyces boulardii*]	32.6	57.23	4.53	1	1	1	1	287	4.72
KQC41816.1	Mitochondrial inner membrane ADP/ATP translocator [*Saccharomyces boulardii*]	34.1	57.10	8.74	1	2	3	3	309	9.70
KQC43435.1	Ribosomal 60S subunit protein L41A [*Saccharomyces boulardii*]	12.2	57.10	7.55	1	1	1	2	106	10.59
KQC41157.1	Exosome core complex catalytic subunit [*Saccharomyces boulardii*]	113.6	56.91	1.40	1	1	2	2	1001	6.67
KQC40920.1	Component of the cleavage and polyadenylation factor I (CF I) [*Saccharomyces boulardii*]	50.2	56.68	2.70	1	1	1	1	445	5.87
KQC41908.1	hypothetical protein AB282_03972 [*Saccharomyces boulardii*]	29.0	56.43	6.27	1	1	1	1	255	9.32
KQC41357.1	Poly(A+) RNA-binding protein [*Saccharomyces boulardii*]	49.1	56.42	4.90	2	2	2	2	429	6.77
KQC42237.1	Subunit of the mRNA cleavage and polyadenlylation factor (CPF) [*Saccharomyces boulardii*]	29.0	56.35	8.08	2	2	2	2	260	9.76
KQC40910.1	Gamma subunit of the translation initiation factor eIF2B [*Saccharomyces boulardii*]	65.7	56.24	4.84	1	2	2	2	578	4.96
KQC43432.1	RNA polymerase II second largest subunit B150 [*Saccharomyces boulardii*]	7.7	56.18	28.57	1	1	1	1	70	9.76
KQC44554.1	Vacuolar endopolyphosphatase with a role in phosphate metabolism [*Saccharomyces boulardii*]	76.9	55.94	2.27	1	1	1	1	661	6.74
KQC45643.1	ENP1p [*Saccharomyces boulardii*]	55.1	55.87	4.97	1	2	2	2	483	4.94
KQC42378.1	Beta subunit of the translation initiation factor eIF2B [*Saccharomyces boulardii*]	42.5	55.66	4.72	1	2	2	2	381	7.93
KQC45508.1	hypothetical protein AB282_00270 [*Saccharomyces boulardii*]	27.1	55.59	5.22	1	1	1	1	230	8.44
KOH52030.1	ADP1p putative ATP-dependent permease of the ABC transporter family [*Saccharomyces boulardii*]	117.2	55.53	2.00	2	2	2	2	1049	5.16
KOH52292.1	FES1p Hsp70 (Ssa1p) nucleotide exchange factor [*Saccharomyces boulardii*]	32.6	55.47	5.52	2	2	2	2	290	5.06
KQC41748.1	Adenine phosphoribosyltransferase [*Saccharomyces boulardii*]	20.5	55.16	12.83	1	2	2	2	187	5.21
KQC42956.1	Peroxisomal acyl-CoA thioesterase [*Saccharomyces boulardii*]	40.2	54.98	7.16	1	2	2	2	349	8.79
KQC41720.1	Subunit of signal peptidase complex [*Saccharomyces boulardii*]	20.8	54.73	8.43	1	1	1	1	178	9.23
KQC43276.1	Acidic protein of the mitochondrial matrix [*Saccharomyces boulardii*]	30.1	54.65	10.53	1	2	2	2	266	4.58
KQC45659.1	Component of the MICOS complex [*Saccharomyces boulardii*]	12.4	54.55	11.32	1	1	1	1	106	8.40
KQC42097.1	Splicing factor associated with the spliceosome [*Saccharomyces boulardii*]	56.5	54.44	2.19	1	1	1	1	503	5.17
KQC45840.1	Rho GTPase activating protein (RhoGAP) [*Saccharomyces boulardii*]	124.8	54.39	1.68	1	1	1	1	1128	6.84
KQC44794.1	putative lipid phosphatase of the endoplasmic reticulum [*Saccharomyces boulardii*]	57.1	54.37	1.63	1	1	1	2	491	9.44
KQC44079.1	Enzyme of the 'de novo' purine nucleotide biosynthetic pathway [*Saccharomyces boulardii*]	86.0	54.12	2.99	1	2	2	2	802	5.27
KQC41017.1	Nuclear actin-related protein involved in chromatin remodeling [*Saccharomyces boulardii*]	100.3	54.08	0.91	1	1	1	1	881	4.82
KQC40964.1	hypothetical protein AB282_04894 [*Saccharomyces boulardii*]	48.0	54.06	4.17	1	1	1	1	432	5.43
KQC44667.1	Peroxisomal membrane protein (PMP) [*Saccharomyces boulardii*]	50.7	53.92	1.81	1	1	1	1	441	6.35
KQC43670.1	Component of the MICOS complex [*Saccharomyces boulardii*]	26.9	53.89	6.44	1	1	1	1	233	9.26
KQC42189.1	Subunit of signal peptidase complex [*Saccharomyces boulardii*]	21.3	53.72	11.41	1	2	2	2	184	8.70

KQC42598.1	Class E protein of the vacuolar protein-sorting (Vps) pathway [*Saccharomyces boulardii*]	23.1	53.67	6.37	4	1	2	2	204	6.87
KQC43300.1	Intracellular sporulation-specific glucoamylase [*Saccharomyces boulardii*]	61.4	53.53	3.46	1	2	2	2	549	5.07
KQC43385.1	Alpha subunit of the nascent polypeptide-associated complex (NAC) [*Saccharomyces boulardii*]	18.7	53.47	12.07	1	2	2	2	174	4.94
KQC40623.1	Dual specificity mitochondrial citrate and methylcitrate synthase [*Saccharomyces boulardii*]	53.8	53.38	4.12	2	2	2	2	486	8.59
KQC42624.1	hypothetical protein AB282_03052 [*Saccharomyces boulardii*]	31.5	53.34	6.18	1	1	1	1	275	9.29
KQC41268.1	Lumazine synthase (DMRL synthase) [*Saccharomyces boulardii*]	18.5	53.32	4.14	1	1	1	1	169	6.80
KQC43989.1	Mitochondrial ribosomal protein of the small subunit [*Saccharomyces boulardii*]	50.8	52.92	4.44	1	2	2	2	450	9.73
KQC41894.1	hypothetical protein AB282_03958 [*Saccharomyces boulardii*]	55.8	52.89	5.32	1	2	2	2	489	7.84
KQC40636.1	Essential helicase component of heterohexameric MCM2-7 complex [*Saccharomyces boulardii*]	105.0	52.86	0.96	1	1	1	1	933	6.21
KQC42291.1	Methyltransferase for rRNA [*Saccharomyces boulardii*]	27.8	52.74	4.37	1	1	1	1	252	8.41
KQC41032.1	Ubiquitin-specific protease [*Saccharomyces boulardii*]	146.3	52.62	0.86	1	1	1	1	1272	5.21
KQC44448.1	GDP dissociation inhibitor [*Saccharomyces boulardii*]	51.2	52.45	7.32	1	2	2	2	451	5.99
KOH52096.1	CDC39p Component of the CCR4-NOT1 core complex [*Saccharomyces boulardii*]	240.2	52.27	0.90	2	2	2	2	2108	6.44
KQC41136.1	Nuclear exosome exonuclease component [*Saccharomyces boulardii*]	84.0	52.25	1.77	1	1	1	1	733	7.14
KQC42731.1	Fe(II)/2-oxoglutarate-dependent dioxygenase family member [*Saccharomyces boulardii*]	35.3	52.02	5.11	1	2	2	2	313	7.99
KQC40649.1	Effector of Rab GTPase Sec4p [*Saccharomyces boulardii*]	114.4	51.97	1.84	1	1	1	2	1033	8.43
KQC44810.1	Essential Hsp90p co-chaperone [*Saccharomyces boulardii*]	58.3	51.86	3.56	1	1	1	1	506	5.06
KQC44722.1	Palmitoyl transferase involved in protein palmitoylation [*Saccharomyces boulardii*]	85.8	51.84	1.18	1	1	1	1	764	5.72
KQC40462.1	Mitochondrial ribosomal protein of the large subunit [*Saccharomyces boulardii*]	33.7	51.84	6.40	1	2	2	2	297	9.45
KQC43203.1	hypothetical protein AB282_01474 [*Saccharomyces boulardii*]	158.2	51.83	1.37	2	2	2	2	1461	5.26
KQC44164.1	Subunit of the multiprotein cohesin complex [*Saccharomyces boulardii*]	141.1	51.55	1.39	1	1	1	1	1225	6.33
KQC43641.1	GTP-cyclohydrolase I [*Saccharomyces boulardii*]	27.8	51.52	8.64	1	2	2	2	243	7.23
KQC42122.1	Nucleolar DEAD-box protein required for ribosome assembly and function [*Saccharomyces boulardii*]	85.0	51.48	1.59	1	1	1	1	754	5.54
KQC41178.1	PAS-domain containing serine/threonine protein kinase [*Saccharomyces boulardii*]	109.0	51.34	1.04	4	1	1	1	960	5.54
KOH49814.1	MPP10p Component of the SSU processome and 90S preribosome [*Saccharomyces boulardii*]	66.9	51.31	4.38	2	2	2	2	593	4.68
KQC42706.1	Essential protein, constituent of 66S pre-ribosomal particle [*Saccharomyces boulardii*]	50.5	51.14	5.53	1	2	2	2	434	9.57
KQC41804.1	Transcriptional coactivator [*Saccharomyces boulardii*]	33.1	51.04	7.88	1	2	2	2	292	5.63
KQC43522.1	Divalent metal ion transporter involved in manganese homeostasis [*Saccharomyces boulardii*]	59.7	51.03	1.82	1	1	1	1	549	5.71
KQC45054.1	UDP-N-acetylglucosamine pyrophosphorylase [*Saccharomyces boulardii*]	53.4	50.96	3.35	2	1	2	2	477	7.42

KQC41248.1	Divalent metal ion transporter [*Saccharomyces boulardii*]	63.1	50.89	3.65	1	1	1	2	575	6.73
KQC44427.1	hypothetical protein AB282_01683 [*Saccharomyces boulardii*]	81.5	50.88	1.42	1	1	1	1	706	7.43
KQC41275.1	hypothetical protein AB282_04717 [*Saccharomyces boulardii*]	38.1	50.73	2.05	1	1	1	1	342	6.00
KQC42607.1	putative ATP-dependent RNA helicase of the DEAD-box family [*Saccharomyces boulardii*]	83.3	50.68	2.02	1	1	1	1	742	9.28
KQC40443.1	Delta adaptin-like subunit of the clathrin associated protein complex [*Saccharomyces boulardii*]	106.8	50.65	1.61	1	1	1	1	932	5.08
KQC42918.1	Subunit of the cytosolic chaperonin Cct ring complex [*Saccharomyces boulardii*]	61.9	50.59	5.34	1	3	3	3	562	5.53
KQC43699.1	Cholinephosphate cytidylyltransferase [*Saccharomyces boulardii*]	49.3	50.57	4.48	1	2	2	2	424	8.57
KQC42428.1	ORM2p that mediates sphingolipid homeostasis [*Saccharomyces boulardii*]	24.8	50.50	8.33	2	2	2	2	216	8.40
KQC44644.1	Anthranilate phosphoribosyl transferase [*Saccharomyces boulardii*]	41.3	50.47	3.16	1	1	1	2	380	6.67
KQC42879.1	Adenosine kinase [*Saccharomyces boulardii*]	36.3	50.43	4.12	1	1	1	1	340	5.16
KQC40447.1	RNA-binding protein, subunit of Nrd1 complex (Nrd1p-Nab3p-Sen1p) [*Saccharomyces boulardii*]	90.6	50.37	3.11	1	2	2	2	804	4.51
KQC44651.1	Mitochondrial ribosomal protein of the small subunit [*Saccharomyces boulardii*]	36.7	50.15	4.36	1	1	1	1	321	9.06
KQC40687.1	Glycine-tRNA synthetase [*Saccharomyces boulardii*]	71.0	50.05	2.27	1	1	1	1	618	7.52
KQC42133.1	Subunit of the core form of RNA polymerase transcription factor TFIIH [*Saccharomyces boulardii*]	52.3	50.00	6.29	1	2	2	2	461	7.80
KQC41152.1	Calmodulin-dependent protein kinase [*Saccharomyces boulardii*]	50.4	49.97	2.46	1	1	1	1	447	6.04
KQC42233.1	Mitogen-activated protein kinase involved in osmoregulation [*Saccharomyces boulardii*]	48.9	49.81	2.76	1	1	1	1	435	5.55
KQC40494.1	Component of both the Rpd3S and Rpd3L histone deacetylase complex [*Saccharomyces boulardii*]	51.0	49.72	5.65	1	2	2	2	460	5.25
KQC42215.1	v-SNARE component of the vacuolar SNARE complex [*Saccharomyces boulardii*]	28.9	49.63	2.77	1	1	1	1	253	5.66
KOH51396.1	PMT1p Protein O-mannosyltransferase of the ER membrane [*Saccharomyces boulardii*]	92.6	49.46	1.35	2	1	1	1	817	6.68
KQC40655.1	Flavin-linked sulfhydryl oxidase localized to the ER lumen [*Saccharomyces boulardii*]	22.1	49.27	6.63	1	1	1	1	196	7.43
KOH52362.1	CHS3p Chitin synthase III [*Saccharomyces boulardii*]	131.5	49.16	2.32	3	2	2	2	1165	6.71
KQC42253.1	Choline kinase [*Saccharomyces boulardii*]	66.3	49.07	1.89	1	1	1	1	582	5.58
KQC43083.1	Subunit of tRNA (1-methyladenosine) methyltransferase with Gcd14p [*Saccharomyces boulardii*]	43.9	49.04	2.87	1	1	1	1	383	7.59
KQC44049.1	YIP4p that interacts with Rab GTPase [*Saccharomyces boulardii*]	26.0	48.89	5.11	1	1	1	1	235	7.01
KQC43271.1	FIS1p involved in mitochondrial fission and peroxisome abundance [*Saccharomyces boulardii*]	17.7	48.86	11.61	1	2	2	2	155	9.10
KQC44414.1	5-phospho-ribosyl-1(alpha)-pyrophosphate synthetase, synthesizes PRPP [*Saccharomyces boulardii*]	34.7	48.68	2.83	2	1	1	1	318	6.46
KQC41324.1	MAP kinase kinase kinase of HOG1 mitogen-activated signaling pathway [*Saccharomyces boulardii*]	180.3	48.63	0.89	1	1	1	1	1579	5.78
KQC45444.1	2-enoyl thioester reductase [*Saccharomyces boulardii*]	42.0	48.34	2.63	1	1	1	1	380	9.00
KQC43694.1	Mitochondrial protein that interacts with frataxin (Yfh1p) [*Saccharomyces boulardii*]	28.7	48.20	8.43	1	2	2	2	261	8.44

KQC44570.1	Transmembrane protein [*Saccharomyces boulardii*]	60.8	48.08	2.25	1	1	1	1	534	4.79
KQC42119.1	SOF1p required for biogenesis of 40S (small) ribosomal subunit [*Saccharomyces boulardii*]	56.8	48.07	3.27	1	1	1	1	489	9.41
KQC44826.1	NUM1p required for nuclear migration [*Saccharomyces boulardii*]	290.6	47.89	5.02	1	1	1	1	2549	5.35
KQC41333.1	hypothetical protein AB282_04194 [*Saccharomyces boulardii*]	47.1	47.84	5.45	1	2	2	2	404	9.39
KQC45016.1	TSR1p required for processing of 20S pre-rRNA in the cytoplasm [*Saccharomyces boulardii*]	90.8	47.80	4.06	4	2	3	3	788	5.69
KQC42946.1	RAD26p involved in transcription-coupled nucleotide excision repair [*Saccharomyces boulardii*]	124.5	47.78	1.38	2	1	2	2	1085	7.47
KQC43249.1	hypothetical protein AB282_01325 [*Saccharomyces boulardii*]	54.9	47.70	3.38	1	2	2	2	473	5.73
KQC44337.1	Phosphatidylserine synthase [*Saccharomyces boulardii*]	30.9	47.69	7.97	1	1	1	1	276	6.27
KQC40974.1	hypothetical protein AB282_04904 [*Saccharomyces boulardii*]	129.6	47.64	2.03	1	1	2	2	1134	9.10
KQC41531.1	Essential serine kinase involved in the processing of 20S pre-rRNA [*Saccharomyces boulardii*]	49.1	47.58	2.35	1	1	1	1	425	5.29
KQC44855.1	hypothetical protein AB282_00916 [*Saccharomyces boulardii*]	88.7	47.39	2.89	1	2	2	2	796	9.20
KQC42192.1	Mitochondrial elongation factor involved in translational elongation [*Saccharomyces boulardii*]	84.6	47.38	2.63	1	1	2	2	761	6.86
KQC41532.1	hypothetical protein AB282_04393 [*Saccharomyces boulardii*]	20.8	47.34	11.11	1	1	1	1	207	7.64
KQC42309.1	ER membrane protein that plays a central role in ERAD [*Saccharomyces boulardii*]	94.1	47.26	1.34	1	1	1	1	820	6.51
KQC40893.1	putative serine hydrolase [*Saccharomyces boulardii*]	30.4	47.14	4.14	1	1	1	1	266	6.32
KQC42349.1	Zinc finger transcription factor [*Saccharomyces boulardii*]	164.1	47.04	0.88	1	1	2	2	1483	7.44
KQC40637.1	Subunit g of the mitochondrial F1F0 ATP synthase [*Saccharomyces boulardii*]	12.9	46.96	16.52	1	2	2	2	115	9.82
KQC45552.1	Mitochondrial ribosomal protein of the small subunit [*Saccharomyces boulardii*]	31.9	46.95	7.91	1	1	1	1	278	10.29
KQC43376.1	hypothetical protein AB282_02372 [*Saccharomyces boulardii*]	69.0	46.91	1.99	1	1	1	1	602	6.23
KQC45845.1	Non-essential INO80 chromatin remodeling complex subunit [*Saccharomyces boulardii*]	22.6	46.84	8.25	2	1	1	1	194	5.45
KQC41249.1	Component of the cleavage and polyadenylation factor I (CF I) [*Saccharomyces boulardii*]	59.7	46.70	2.81	1	1	1	1	534	5.66
KQC41829.1	Mitochondrial DNA-binding protein [*Saccharomyces boulardii*]	21.5	46.67	6.56	1	1	1	1	183	9.69
KQC42569.1	Mitochondrial cytochrome-c peroxidase [*Saccharomyces boulardii*]	40.4	46.57	6.63	1	2	2	2	362	6.64
KQC44446.1	Type 1 serine/threonine protein phosphatase catalytic subunit [*Saccharomyces boulardii*]	35.9	46.50	4.49	3	1	2	2	312	5.49
KQC41325.1	Alpha-1,6-mannosyltransferase localized to the ER [*Saccharomyces boulardii*]	62.6	46.15	1.45	1	1	1	1	551	7.09
KQC42194.1	Subunit of the RNA polymerase II mediator complex [*Saccharomyces boulardii*]	122.4	46.06	1.12	1	1	1	1	1073	8.81
KQC44432.1	Vacuolar aspartate and glutamate exporter [*Saccharomyces boulardii*]	48.8	45.93	4.24	1	1	1	1	448	8.66
KQC42440.1	Signal transducing MEK kinase [*Saccharomyces boulardii*]	83.1	45.90	1.49	1	1	1	1	738	7.23
KQC45446.1	Phosphatidate cytidylyltransferase (CDP-diglyceride synthetase) [*Saccharomyces boulardii*]	51.8	45.88	3.28	1	1	2	2	457	8.57
KOH49753.1	SMC3p Subunit of the multiprotein cohesin complex [*Saccharomyces boulardii*]	48.3	45.84	3.85	10	1	2	2	416	8.59

KQC41359.1	LST8p required for the transport of Gap1p [*Saccharomyces boulardii*]	34.0	45.81	3.63	1	1	1	1	303	6.52
KQC45213.1	SOL2p with a possible role in tRNA export [*Saccharomyces boulardii*]	34.5	45.78	9.84	1	1	2	2	315	6.70
KQC42337.1	Calcium permeable gated cation channel [*Saccharomyces boulardii*]	89.3	45.76	1.15	1	1	1	1	782	8.12
KQC42435.1	Component of the RSC chromatin remodeling complex [*Saccharomyces boulardii*]	102.2	45.68	2.92	1	2	2	2	889	8.91
KQC42818.1	Member of the REF (RNA and export factor binding protein) family [*Saccharomyces boulardii*]	23.8	45.60	9.85	1	2	2	2	203	9.41
KQC44602.1	Subunit of U3-containing Small Subunit (SSU) processome complex [*Saccharomyces boulardii*]	71.9	45.56	1.71	1	1	1	1	643	4.94
KOH49752.1	SMC3p Subunit of the multiprotein cohesin complex [*Saccharomyces boulardii*]	82.0	45.53	3.47	2	1	1	1	720	5.80
KQC43437.1	hypothetical protein AB282_02433 [*Saccharomyces boulardii*]	34.2	45.49	6.44	1	1	1	3	326	5.41
KQC43735.1	hypothetical protein AB282_01991 [*Saccharomyces boulardii*]	29.2	45.47	3.80	1	1	1	1	263	9.98
KQC43534.1	hypothetical protein AB282_02530 [*Saccharomyces boulardii*]	71.3	45.31	1.55	1	1	1	1	644	7.69
KQC41320.1	SOL1p with a possible role in tRNA export [*Saccharomyces boulardii*]	35.6	45.28	7.17	1	1	2	2	321	7.55
KQC43743.1	Nuclear 5' to 3' single-stranded RNA exonuclease [*Saccharomyces boulardii*]	30.4	45.27	3.31	1	1	1	1	272	9.17
KQC40645.1	Membrane protein that interacts with Yip1p to mediate membrane traffic [*Saccharomyces boulardii*]	22.8	45.19	3.96	1	1	1	1	202	9.03
KQC43046.1	Alpha arrestin [*Saccharomyces boulardii*]	114.8	45.07	1.17	2	1	1	1	1023	7.15
KQC45151.1	NHP2p [*Saccharomyces boulardii*]	17.1	45.05	5.13	1	1	1	1	156	9.72
KQC45729.1	Vesicle membrane receptor protein (v-SNARE) [*Saccharomyces boulardii*]	14.0	45.03	9.68	1	1	1	1	124	8.18
KQC43471.1	Aldose reductase [*Saccharomyces boulardii*]	37.1	45.02	4.89	1	2	2	2	327	7.08
KQC41712.1	Subunit of the THO complex [*Saccharomyces boulardii*]	45.0	44.85	3.06	4	1	2	2	392	4.54
KQC44947.1	FAF1p required for pre-rRNA processing [*Saccharomyces boulardii*]	45.2	44.83	2.76	1	1	1	1	399	9.00
KQC41001.1	Beta 2 subunit of the 20S proteasome [*Saccharomyces boulardii*]	28.2	44.73	8.05	1	2	2	2	261	6.79
KQC43883.1	Component of the conserved oligomeric Golgi complex [*Saccharomyces boulardii*]	31.8	44.56	6.09	1	1	1	1	279	4.68
KQC44271.1	Soluble fumarate reductase [*Saccharomyces boulardii*]	67.7	44.08	1.62	1	1	1	1	618	5.44
KQC41610.1	MON2p with a role in endocytosis and vacuole integrity [*Saccharomyces boulardii*]	186.7	44.02	0.98	1	1	2	2	1636	5.73
KOH52340.1	REB1p RNA polymerase I enhancer binding protein [*Saccharomyces boulardii*]	91.8	43.96	2.35	2	2	2	2	810	5.16
KQC43113.1	Mannose-containing glycoprotein constituent of the cell wall [*Saccharomyces boulardii*]	23.0	43.65	3.56	5	1	1	1	225	4.68
KQC40780.1	Ski complex component and TPR protein [*Saccharomyces boulardii*]	163.7	43.59	1.12	1	1	1	1	1432	5.38
KOH50127.1	PRI1p Subunit of DNA primase [*Saccharomyces boulardii*]	24.1	43.52	5.39	2	1	1	1	204	9.22
KQC45425.1	Succinate semialdehyde dehydrogenase [*Saccharomyces boulardii*]	54.2	43.47	3.82	1	1	1	1	497	6.65
KQC43004.1	Microtubule-associated protein involved in microtubule organization [*Saccharomyces boulardii*]	194.5	43.44	1.17	2	2	2	2	1796	5.94
KQC45290.1	Essential protein involved in rRNA processing and ribosome biogenesis [*Saccharomyces boulardii*]	34.4	43.20	3.03	1	1	1	1	297	8.87

KQC42278.1	Mitochondrial intermembrane space protein [*Saccharomyces boulardii*]	42.2	43.11	2.45	1	1	1	1	367	7.93
KQC45229.1	Component of the RSC chromatin remodeling complex [*Saccharomyces boulardii*]	54.2	42.97	4.14	1	1	1	2	483	4.86
KQC41058.1	Glucose-repressible ADP-ribosylation factor [*Saccharomyces boulardii*]	20.7	42.96	5.46	1	1	1	1	183	9.41
KQC40896.1	Phosphoprotein of the mRNA cap-binding complex [*Saccharomyces boulardii*]	18.2	42.81	8.70	1	1	1	1	161	5.96
KQC41526.1	epimerase [*Saccharomyces boulardii*]	27.5	42.37	6.50	1	1	1	1	246	8.29
KQC43312.1	Histidinol-phosphate aminotransferase [*Saccharomyces boulardii*]	42.6	42.20	3.64	1	1	1	1	385	6.43
KQC44576.1	Sorting nexin [*Saccharomyces boulardii*]	70.7	42.06	5.28	1	2	2	2	625	6.83
KQC41702.1	Component of the conserved oligomeric Golgi complex [*Saccharomyces boulardii*]	31.1	41.94	5.22	2	1	1	1	268	4.70
KQC43129.1	Subunit of the SWI/SNF chromatin remodeling complex [*Saccharomyces boulardii*]	92.9	41.83	1.09	1	1	1	1	825	4.86
KQC40419.1	hypothetical protein AB282_05053 [*Saccharomyces boulardii*]	78.3	41.79	2.33	1	1	1	1	688	5.44
KQC43577.1	Ceramide synthase component [*Saccharomyces boulardii*]	48.4	41.69	4.87	1	1	1	1	411	8.79
KQC45150.1	Cytoplasmic nucleoporin required for polyadenylated mRNA export [*Saccharomyces boulardii*]	62.1	41.65	2.04	1	1	1	1	538	8.92
KQC41947.1	VTI1p involved in cis-Golgi membrane traffic [*Saccharomyces boulardii*]	24.7	41.50	6.45	1	1	1	1	217	6.62
KQC44522.1	3,4-dihydroxy-2-butanone-4-phosphate synthase (DHBP synthase) [*Saccharomyces boulardii*]	22.6	40.87	3.37	1	1	1	1	208	5.78
KQC43625.1	Constituent of 66S pre-ribosomal particle [*Saccharomyces boulardii*]	35.0	40.66	3.87	1	1	1	1	310	9.63
KQC43000.1	Sorting nexin [*Saccharomyces boulardii*]	49.0	40.56	2.60	1	1	1	1	423	6.49
KQC44301.1	Phosphorylated and palmitoylated vacuolar membrane protein [*Saccharomyces boulardii*]	63.2	40.54	4.15	1	1	2	2	578	5.14
KQC42807.1	putative positive regulator of mannosylphosphate transferase Mnn6p [*Saccharomyces boulardii*]	138.5	40.51	1.71	1	2	2	2	1171	6.61
KQC40978.1	3-phosphoserine aminotransferase [*Saccharomyces boulardii*]	43.4	40.43	5.06	1	1	1	1	395	6.54
KQC44758.1	Histone H2A [*Saccharomyces boulardii*]	14.0	40.37	6.82	2	1	1	1	132	10.67
KQC40926.1	Pseudouridine synthase [*Saccharomyces boulardii*]	77.0	40.31	3.40	1	1	1	1	676	7.80
KQC44179.1	Small subunit of the heterodimeric cap binding complex with Sto1p [*Saccharomyces boulardii*]	93.3	40.30	1.37	2	1	1	1	802	8.95
KQC45166.1	Component of the septin ring that is required for cytokinesis [*Saccharomyces boulardii*]	62.6	40.25	4.72	1	2	2	2	551	5.53
KQC40744.1	Repressor of translation initiation [*Saccharomyces boulardii*]	39.2	40.13	4.58	1	1	1	1	349	8.43
KQC42301.1	PWP1p with WD-40 repeats involved in rRNA processing [*Saccharomyces boulardii*]	63.8	40.06	2.26	1	1	1	1	576	4.60
KQC44991.1	U1 snRNP protein involved in splicing [*Saccharomyces boulardii*]	63.0	39.92	1.89	1	1	1	1	530	6.57
KQC40416.1	hypothetical protein AB282_05050 [*Saccharomyces boulardii*]	17.4	39.86	15.75	1	2	2	2	146	5.30
KQC43927.1	SDS23p involved in cell separation during budding [*Saccharomyces boulardii*]	56.7	39.86	2.33	1	1	1	1	515	9.04
KQC44698.1	MHR1p [*Saccharomyces boulardii*]	26.9	39.75	6.19	1	1	1	1	226	9.50
KQC44519.1	Serine/threonine protein kinase [*Saccharomyces boulardii*]	86.1	39.74	1.44	1	1	1	1	766	9.23

KQC45063.1	Exosome non-catalytic core component [*Saccharomyces boulardii*]	29.0	39.61	5.28	1	1	1	1	265	5.33
KQC41964.1	SCJ1p [*Saccharomyces boulardii*]	41.5	39.60	4.24	1	1	1	1	377	7.43
KQC43889.1	C-24(28) sterol reductase [*Saccharomyces boulardii*]	56.0	39.48	3.81	1	2	2	2	473	8.60
KQC42306.1	Specific translational activator for the mitochondrial COX1 mRNA [*Saccharomyces boulardii*]	50.8	39.40	2.06	1	1	1	1	436	8.73
KQC41335.1	Acyl-CoA:sterol acyltransferase [*Saccharomyces boulardii*]	74.0	39.27	2.65	1	2	2	2	642	7.72
KQC41358.1	Mitochondrial ribosomal protein of the large subunit [*Saccharomyces boulardii*]	43.2	39.16	3.50	1	1	1	1	371	9.82
KQC40876.1	hypothetical protein AB282_04806 [*Saccharomyces boulardii*]	57.2	39.13	2.51	4	1	2	2	479	9.17
KQC42716.1	Monoglyceride lipase (MGL) [*Saccharomyces boulardii*]	35.5	39.08	5.43	1	1	1	1	313	7.87
KQC43878.1	hypothetical protein AB282_02134 [*Saccharomyces boulardii*]	28.3	39.04	6.10	1	1	1	1	246	4.88
KQC42553.1	Subunit of Nup84p subcomplex of nuclear pore complex (NPC) [*Saccharomyces boulardii*]	133.2	38.57	0.86	1	1	1	1	1157	5.11
KQC43656.1	hypothetical protein AB282_01912 [*Saccharomyces boulardii*]	89.5	38.52	2.18	1	2	2	2	781	5.40
KQC45230.1	hypothetical protein AB282_00502 [*Saccharomyces boulardii*]	24.7	38.45	4.05	1	1	1	1	222	4.63
KQC40515.1	Isopentenyl diphosphate:dimethylallyl diphosphate isomerase [*Saccharomyces boulardii*]	33.3	38.44	10.42	3	2	2	2	288	4.98
KQC44360.1	Cysteine desulfurase (Nfs1p) activator [*Saccharomyces boulardii*]	11.2	38.25	8.51	1	1	1	1	94	10.27
KQC40884.1	ATPase component of the RSC chromatin remodeling complex [*Saccharomyces boulardii*]	194.3	38.01	0.70	1	1	2	2	1706	6.93
KQC43731.1	Mitochondrial ribosomal protein of the small subunit [*Saccharomyces boulardii*]	39.6	37.96	3.77	1	1	1	1	345	9.88
KQC44340.1	Core Sm protein Sm B [*Saccharomyces boulardii*]	22.4	37.92	12.76	1	2	2	2	196	10.36
KQC45313.1	Conserved component of the MICOS complex [*Saccharomyces boulardii*]	10.4	37.91	10.31	1	1	1	1	97	10.20
KQC44141.1	Cytoplasmic protein that inhibits Gdb1p glycogen debranching activity [*Saccharomyces boulardii*]	21.8	37.73	7.69	1	2	2	2	195	7.18
KQC40678.1	Spermidine synthase [*Saccharomyces boulardii*]	33.3	37.72	7.17	1	2	2	2	293	5.53
KQC42507.1	Mitochondrial ribosomal protein of the large subunit [*Saccharomyces boulardii*]	36.9	37.70	4.08	1	1	1	1	319	8.25
KQC45394.1	Lsm (Like Sm) protein [*Saccharomyces boulardii*]	11.2	37.55	13.68	1	1	1	1	95	8.13
KQC40395.1	hypothetical protein AB282_05029 [*Saccharomyces boulardii*]	57.3	37.49	2.49	1	1	1	2	523	5.81
KQC43145.1	Elongase I, medium-chain acyl elongase [*Saccharomyces boulardii*]	36.3	37.48	5.48	1	2	2	2	310	9.39
KQC40378.1	Essential 18S rRNA dimethylase (dimethyladenosine transferase) [*Saccharomyces boulardii*]	35.9	37.42	3.14	1	1	1	1	318	9.55
KQC43573.1	ubiquinol--cytochrome-c reductase subunit 10 [*Saccharomyces boulardii*]	8.6	37.39	9.09	1	1	1	1	77	8.53
KQC40759.1	Mitochondrial ribosomal protein of the small subunit [*Saccharomyces boulardii*]	13.5	37.19	6.09	1	1	1	1	115	11.03
KQC42690.1	Endoplasmic reticulum transmembrane protein [*Saccharomyces boulardii*]	23.4	37.18	4.85	1	1	1	1	206	9.77
KQC45099.1	Nucleolar protein [*Saccharomyces boulardii*]	94.3	37.10	1.98	1	1	1	1	810	7.18
KQC41043.1	Component of nuclear aminoacylation-dependent tRNA export pathway [*Saccharomyces boulardii*]	84.7	36.91	1.31	1	1	1	2	761	5.41

KQC42821.1	Monocarboxylate/proton symporter of the plasma membrane [*Saccharomyces boulardii*]	69.4	36.83	4.22	1	1	1	1	616	5.80
KQC41196.1	Bisphosphate-3'-nucleotidase [*Saccharomyces boulardii*]	39.1	36.82	3.64	1	1	1	1	357	6.20
KQC44268.1	Subunit D of the V1 peripheral membrane domain of V-ATPase [*Saccharomyces boulardii*]	29.2	36.80	11.33	1	1	1	1	256	5.92
KQC41861.1	SPG4p required for high temperature survival during stationary phase [*Saccharomyces boulardii*]	13.2	36.66	9.57	1	1	1	1	115	6.80
KQC41172.1	Ribosomal protein P2 alpha [*Saccharomyces boulardii*]	10.7	36.56	11.32	1	1	1	1	106	4.04
KQC44330.1	Inositol phosphosphingolipid phospholipase C [*Saccharomyces boulardii*]	53.9	36.54	3.77	1	1	1	1	477	7.53
KQC45301.1	Subunit of the mitochondrial (mt) i-AAA protease supercomplex [*Saccharomyces boulardii*]	47.1	36.44	2.40	1	1	1	1	417	9.32
KOH51847.1	RPB7p RNA polymerase II subunit B16 [*Saccharomyces boulardii*]	16.6	36.33	8.67	2	1	1	1	150	7.30
KQC43338.1	Component of RNA polymerase transcription factor TFIIH holoenzyme [*Saccharomyces boulardii*]	95.3	36.30	1.07	1	1	1	1	843	6.05
KQC44386.1	Subunit of the vacuolar transporter chaperone (VTC) complex [*Saccharomyces boulardii*]	14.4	36.29	4.65	1	1	1	1	129	10.52
KQC41489.1	hypothetical protein AB282_04350 [*Saccharomyces boulardii*]	33.6	36.28	8.03	1	1	2	2	299	6.79
KQC45764.1	N-succinyl-5-aminoimidazole-4-carboxamide ribotide synthetase [*Saccharomyces boulardii*]	34.6	36.12	8.17	1	1	1	1	306	5.95
KQC42883.1	Mitochondrial ribosomal protein of the small subunit [*Saccharomyces boulardii*]	30.2	36.02	4.51	1	1	1	1	266	8.59
KOH49410.1	SET3p Defining member of the SET3 histone deacetylase complex [*Saccharomyces boulardii*]	85.4	35.91	2.13	2	1	1	1	751	8.73
KQC41452.1	Cytochrome b5 [*Saccharomyces boulardii*]	13.3	35.70	14.17	1	2	2	2	120	4.49
KQC44693.1	RNA-binding subunit of the mRNA cleavage and polyadenylation factor [*Saccharomyces boulardii*]	153.3	35.67	0.59	1	1	1	1	1357	5.91
KQC43891.1	Member of the PUF protein family [*Saccharomyces boulardii*]	98.3	35.60	3.36	1	1	2	2	893	6.55
KQC45344.1	hypothetical protein AB282_00106 [*Saccharomyces boulardii*]	52.0	35.58	4.29	1	1	2	2	466	9.06
KQC42844.1	PMT4p O-mannosyltransferase [*Saccharomyces boulardii*]	87.9	35.57	1.44	1	1	1	1	762	6.93
KQC40828.1	hypothetical protein AB282_04758 [*Saccharomyces boulardii*]	79.9	35.49	3.61	1	1	1	1	692	8.15
KQC41408.1	Subunit of tRNA (1-methyladenosine) methyltransferase [*Saccharomyces boulardii*]	54.3	35.35	2.09	1	1	1	1	478	7.61
KQC44074.1	Central component of GID Complex, involved in FBPase degradation [*Saccharomyces boulardii*]	108.1	35.34	1.15	1	1	1	1	958	4.65
KQC42550.1	Mitochondrial ribosomal protein of the large subunit [*Saccharomyces boulardii*]	22.4	35.32	4.62	1	1	1	1	195	10.11
KQC44879.1	Subunit of the Sec61p translocation complex (Sec61p-Sss1p-Sbh1p) [*Saccharomyces boulardii*]	8.9	35.11	12.50	1	1	1	1	80	9.45
KQC40594.1	tRNA(Ser) Um(44) 2'-O-methyltransferase [*Saccharomyces boulardii*]	64.9	35.03	1.76	1	1	1	2	567	8.56
KQC42630.1	Adenylylsulfate kinase [*Saccharomyces boulardii*]	23.0	35.01	6.44	1	1	1	1	202	8.13
KQC43486.1	RNA binding protein, component of the U1 snRNP protein [*Saccharomyces boulardii*]	56.9	34.86	2.10	1	1	1	1	523	8.37
KQC44640.1	Component of the NuA4 histone acetyltransferase complex [*Saccharomyces boulardii*]	112.4	34.83	1.22	1	1	1	1	982	9.04
KQC44144.1	Calmodulin-dependent protein kinase [*Saccharomyces boulardii*]	50.3	34.80	2.24	1	1	1	1	446	6.27

KQC41458.1	Catalytic subunit of the Dcp1p-Dcp2p decapping enzyme complex [*Saccharomyces boulardii*]	108.5	34.79	0.72	1	1	1	1	969	5.88
KQC41832.1	Essential component of the Rpd3S histone deacetylase complex [*Saccharomyces boulardii*]	78.8	34.77	1.90	1	1	1	1	684	8.78
KQC44867.1	Integral membrane protein [*Saccharomyces boulardii*]	15.9	34.50	18.88	1	2	2	2	143	4.56
KQC40925.1	Catalytic subunit of the histone acetyltransferase complex (NuA4) [*Saccharomyces boulardii*]	52.6	34.44	3.15	1	1	1	1	445	7.71
KQC42941.1	Subunit of the Nup84p subcomplex of the nuclear pore complex (NPC) [*Saccharomyces boulardii*]	84.8	34.41	1.61	1	1	1	1	744	4.65
KQC41042.1	Zinc-finger transcription factor [*Saccharomyces boulardii*]	100.7	33.88	1.21	1	1	1	1	910	7.36
KQC41871.1	Component of the kinetochore-associated Ndc80 complex [*Saccharomyces boulardii*]	24.6	33.82	5.63	1	1	1	1	213	4.96
KQC42451.1	Zinc-finger protein [*Saccharomyces boulardii*]	36.9	33.78	3.58	1	1	1	1	335	9.25
KQC43500.1	Member of an oxysterol-binding protein family [*Saccharomyces boulardii*]	113.7	33.76	1.61	1	1	1	1	996	8.57
KQC42943.1	Voltage-gated chloride channel [*Saccharomyces boulardii*]	72.1	33.60	2.79	2	1	1	1	646	5.41
KQC43548.1	homoserine kinase [*Saccharomyces boulardii*]	38.7	33.47	2.52	1	1	1	1	357	5.41
KQC40424.1	GTPase required for ribosomal subunit synthesis and rRNA processing [*Saccharomyces boulardii*]	135.5	33.42	0.85	1	1	1	1	1183	6.81
KQC44389.1	Integral membrane protein required for ER to Golgi transport [*Saccharomyces boulardii*]	9.4	33.29	18.82	1	1	1	1	85	9.52
KQC43661.1	SDA1p required for actin organization and passage through Start [*Saccharomyces boulardii*]	86.6	33.27	1.04	1	1	1	1	767	6.93
KQC43696.1	ATP-binding protein [*Saccharomyces boulardii*]	33.3	33.26	3.10	1	1	1	1	290	5.41
KOH51140.1	RSM18p Mitochondrial ribosomal protein of the small subunit [*Saccharomyces boulardii*]	17.4	32.86	8.50	2	1	1	1	153	10.83
KQC41594.1	C-14 sterol reductase [*Saccharomyces boulardii*]	50.6	32.79	1.60	1	1	1	1	438	8.85
KQC45235.1	PER1p of the endoplasmic reticulum [*Saccharomyces boulardii*]	57.8	32.76	2.45	1	1	1	1	490	6.27
KQC41773.1	Phospholipase B (lysophospholipase) involved in lipid metabolism [*Saccharomyces boulardii*]	71.1	32.76	1.67	1	1	1	1	658	4.75
KQC45637.1	hypothetical protein AB282_00399 [*Saccharomyces boulardii*]	53.6	32.73	1.64	1	1	1	1	488	7.20
KQC41517.1	Karyopherin alpha [*Saccharomyces boulardii*]	60.4	32.72	1.29	1	1	1	1	542	4.91
KQC45632.1	Methyltransferase [*Saccharomyces boulardii*]	50.4	32.71	2.29	1	1	1	1	436	7.72
KQC42033.1	3'-5' exoribonuclease [*Saccharomyces boulardii*]	110.7	32.56	1.03	1	1	1	1	969	9.04
KQC42911.1	Deoxyhypusine hydroxylase [*Saccharomyces boulardii*]	36.1	32.46	2.77	1	1	1	1	325	4.87
KQC40627.1	2-methylisocitrate lyase of the mitochondrial matrix [*Saccharomyces boulardii*]	64.9	32.38	1.57	1	1	1	1	575	7.56
KQC44684.1	Subunit of TFIIH and nucleotide excision repair factor 3 complex [*Saccharomyces boulardii*]	72.8	32.26	2.34	1	1	1	1	642	6.90
KQC40754.1	Cyclin (Bur2p)-dependent protein kinase [*Saccharomyces boulardii*]	74.2	32.10	3.04	1	2	2	2	657	9.52
KQC42768.1	Aminopeptidase yscII [*Saccharomyces boulardii*]	97.4	31.92	1.86	1	1	1	1	861	5.99
KQC44709.1	GCN2p kinase [*Saccharomyces boulardii*]	187.0	31.90	0.37	1	1	1	1	1633	6.48
KQC43039.1	Core subunit of the RENT complex [*Saccharomyces boulardii*]	126.7	31.90	0.85	1	1	1	1	1173	7.55

KQC44496.1	EUG1p disulfide isomerase of the endoplasmic reticulum lumen [*Saccharomyces boulardii*]	59.0	31.61	2.51	1	1	1	1	517	4.96
KQC40405.1	hypothetical protein AB282_05039 [*Saccharomyces boulardii*]	40.7	31.49	2.75	1	1	1	1	364	5.16
KQC43463.1	cystathionine beta-lyase [*Saccharomyces boulardii*]	42.4	31.42	3.17	1	1	1	1	378	5.91
KQC43691.1	hypothetical protein AB282_01947 [*Saccharomyces boulardii*]	45.2	31.41	1.46	1	1	1	1	411	8.03
KQC40667.1	Essential 121 kDa subunit of the exocyst complex [*Saccharomyces boulardii*]	160.9	31.32	1.20	1	1	1	1	1412	7.28
KQC44917.1	Ubiquitin-conjugating enzyme [*Saccharomyces boulardii*]	16.3	31.29	7.43	2	1	1	1	148	6.77
KQC45312.1	Zinc metalloendopeptidase [*Saccharomyces boulardii*]	78.8	31.14	1.31	1	1	1	1	686	5.72
KQC43289.1	hypothetical protein AB282_01365 [*Saccharomyces boulardii*]	16.9	31.13	5.10	1	1	1	1	157	9.99
KQC42489.1	Dihydroorotase [*Saccharomyces boulardii*]	40.4	31.12	2.20	1	1	1	1	364	6.54
KQC40840.1	RNA polymerase I subunit A43 [*Saccharomyces boulardii*]	36.2	31.12	3.68	1	1	1	1	326	4.89
KQC44036.1	GTS1p involved in Arf3p regulation and in transcription regulation [*Saccharomyces boulardii*]	44.3	31.08	3.30	1	1	1	1	394	7.78
KQC40846.1	Mitochondrial inner membrane Mg(2+) channel [*Saccharomyces boulardii*]	54.2	30.87	1.06	1	1	1	1	470	9.38
KQC43027.1	Mitochondrial protein required for cytochrome c oxidase assembly [*Saccharomyces boulardii*]	9.9	30.81	24.71	1	1	1	1	85	9.36
KQC44783.1	RNA-binding protein [*Saccharomyces boulardii*]	59.8	30.80	1.88	1	1	1	1	533	9.63
KQC42339.1	Methionine aminopeptidase [*Saccharomyces boulardii*]	43.3	30.75	6.20	1	2	2	2	387	7.88
KQC44796.1	Subunit of cohesin loading factor (Scc2p-Scc4p) [*Saccharomyces boulardii*]	171.0	30.74	0.40	1	1	1	1	1493	6.90
KQC45049.1	Cis-golgi localized protein involved in ER to Golgi transport [*Saccharomyces boulardii*]	38.2	30.73	4.40	1	1	1	1	341	4.58
KQC43390.1	Subunit of the glycosylphosphatidylinositol transamidase complex [*Saccharomyces boulardii*]	68.7	30.58	1.97	1	1	1	1	610	5.31
KQC41459.1	Ubiquitin-binding protein [*Saccharomyces boulardii*]	56.4	30.50	1.62	1	1	1	1	493	6.13
KQC41207.1	Non-catalytic subunit of the NatB N-terminal acetyltransferase [*Saccharomyces boulardii*]	92.7	30.50	1.13	1	1	1	1	796	7.84
KQC41536.1	Cytoplasmic protein [*Saccharomyces boulardii*]	88.8	30.42	1.15	1	1	1	1	782	5.10
KQC45419.1	hypothetical protein AB282_00181 [*Saccharomyces boulardii*]	11.5	30.26	5.77	1	1	1	1	104	6.68
KQC41986.1	Subunit (17 kDa) of TFIID and SAGA complex [*Saccharomyces boulardii*]	17.3	30.22	15.92	1	1	1	1	157	6.32
KOH47293.1	SEC16p COPII vesicle coat protein required for ER transport vesicle budding [*Saccharomyces boulardii*]	241.5	30.09	0.46	2	1	1	1	2195	5.38
KQC43272.1	Minor isoform of large subunit of ribonucleotide-diphosphate reductase [*Saccharomyces boulardii*]	99.2	30.01	0.90	2	1	1	1	885	6.21
KQC42086.1	Cofilin, involved in pH-dependent actin filament depolarization [*Saccharomyces boulardii*]	17.6	29.98	8.97	1	1	1	1	156	5.68
KQC42575.1	hypothetical protein AB282_03003 [*Saccharomyces boulardii*]	31.6	29.79	3.28	1	1	1	1	274	9.57
KQC44134.1	LSB3p containing a C-terminal SH3 domain [*Saccharomyces boulardii*]	48.5	29.68	1.77	2	1	1	1	451	6.05
KQC44131.1	Plasma membrane associated protein phosphatase [*Saccharomyces boulardii*]	61.9	29.68	2.36	1	1	1	1	551	7.97
KQC44221.1	DNA dependent ATPase/DNA helicase [*Saccharomyces boulardii*]	126.9	29.64	0.80	1	1	1	1	1121	9.42

KQC40855.1	Alpha-arrestin involved in ubiquitin-dependent endocytosis [*Saccharomyces boulardii*]	89.8	29.60	1.83	1	1	1	1	818	8.54
KQC41604.1	Subunit of heteropentameric Replication factor C (RF-C) [*Saccharomyces boulardii*]	38.2	29.52	1.76	1	1	1	1	340	6.44
KQC40453.1	WD40 domain-containing protein involved in endosomal recycling [*Saccharomyces boulardii*]	114.4	29.46	0.79	1	1	1	1	1013	6.18
KQC43674.1	Subunit of the prohibitin complex (Phb1p-Phb2p) [*Saccharomyces boulardii*]	34.4	29.44	8.39	1	2	2	2	310	9.92
KQC44525.1	Component of the GARP (Golgi-associated retrograde protein) complex [*Saccharomyces boulardii*]	74.3	29.40	1.87	1	1	1	1	641	5.76
KQC42795.1	FATty acid transporter [*Saccharomyces boulardii*]	81.0	29.22	2.00	1	1	2	2	750	5.05
KQC45480.1	High-affinity leucine permease [*Saccharomyces boulardii*]	67.7	29.17	1.64	1	1	1	1	609	8.24
KQC44834.1	Golgi-localized, leucine-zipper domain containing protein [*Saccharomyces boulardii*]	194.6	29.09	0.41	1	1	1	1	1698	5.69
KQC44057.1	One of three beta subunits of the Snf1 kinase complex [*Saccharomyces boulardii*]	46.5	29.06	1.93	2	1	1	1	415	6.23
KQC40537.1	Essential subunit of Sec63 complex [*Saccharomyces boulardii*]	31.3	28.98	3.65	1	1	1	1	274	9.72
KQC40506.1	U3 snoRNP protein [*Saccharomyces boulardii*]	101.1	28.92	1.12	1	1	1	1	896	6.32
KQC41997.1	RING domain E3 ubiquitin ligase [*Saccharomyces boulardii*]	180.1	28.85	0.70	1	1	1	1	1562	5.53
KOH48992.1	REX2p 3'-5' RNA exonuclease [*Saccharomyces boulardii*]	30.9	28.72	4.46	2	1	1	1	269	6.86
KQC42362.1	hypothetical protein AB282_03544 [*Saccharomyces boulardii*]	31.9	28.66	2.55	1	1	1	1	274	6.81
KQC42461.1	Enzyme regulator [*Saccharomyces boulardii*]	99.7	28.58	0.91	1	1	1	1	880	5.50
KQC42285.1	Inhibitor of carboxypeptidase Y (Prc1p), and Ras GAP (Ira2p) [*Saccharomyces boulardii*]	24.3	28.57	6.85	1	1	1	1	219	6.42
KQC41659.1	hypothetical protein AB282_03723 [*Saccharomyces boulardii*]	40.1	28.54	5.60	1	1	1	1	357	9.64
KQC45719.1	Cytochrome c heme lyase (holocytochrome c synthase) [*Saccharomyces boulardii*]	30.1	28.53	3.35	1	1	1	1	269	5.50
KQC43815.1	Component of the nonsense-mediated mRNA decay (NMD) pathway [*Saccharomyces boulardii*]	45.0	28.47	2.84	1	1	1	1	387	9.85
KQC44535.1	Splicing factor [*Saccharomyces boulardii*]	55.8	28.37	1.92	1	1	1	1	469	8.57
KOH48286.1	RPC19p RNA polymerase subunit AC19 [*Saccharomyces boulardii*]	11.5	28.33	11.00	2	1	1	1	100	4.35
KQC42888.1	reductase superfamily protein [*Saccharomyces boulardii*]	32.3	28.00	3.55	1	1	1	1	282	6.93
KQC41988.1	putative mannosidase [*Saccharomyces boulardii*]	50.5	27.91	1.75	1	1	1	1	458	4.75
KQC40747.1	Mitochondrial inner membrane protein [*Saccharomyces boulardii*]	52.1	27.89	2.86	1	1	1	1	454	9.33
KQC44009.1	Component of the RNA polymerase II mediator complex [*Saccharomyces boulardii*]	128.7	27.84	1.06	1	1	1	1	1132	5.10
KQC44949.1	T subunit of the mitochondrial glycine decarboxylase complex [*Saccharomyces boulardii*]	44.4	27.69	2.25	1	1	1	1	400	8.84
KQC41307.1	Ser/Thr protein kinase [*Saccharomyces boulardii*]	100.5	27.57	2.13	1	1	1	1	893	8.09
KQC44481.1	Glycerol proton symporter of the plasma membrane [*Saccharomyces boulardii*]	63.6	27.52	1.76	1	1	1	1	569	6.55
KQC41473.1	Peptidyl-prolyl cis-trans isomerase (PPIase) [*Saccharomyces boulardii*]	12.2	27.50	7.02	1	1	1	1	114	6.04
KQC41300.1	Essential nucleolar protein involved in pre-18S rRNA processing [*Saccharomyces boulardii*]	36.4	27.46	3.16	1	1	1	1	316	7.97

KQC42903.1	AIM24p with a role in determining mitochondrial architecture [*Saccharomyces boulardii*]	44.4	27.40	1.78	1	1	1	1	394	9.80
KQC43089.1	Mitochondrial translation initiation factor 3 (IF3, mIF3) [*Saccharomyces boulardii*]	36.3	27.38	3.85	1	1	1	1	312	9.89
KQC43299.1	Mitochondrial matrix protein [*Saccharomyces boulardii*]	18.4	27.37	5.16	1	1	1	1	155	9.67
KQC41569.1	RNA-binding subunit of Nrd1 complex [*Saccharomyces boulardii*]	63.8	27.37	1.22	1	1	1	1	575	9.17
KQC44190.1	Activator of Cbk1p kinase [*Saccharomyces boulardii*]	33.2	27.33	4.88	1	1	1	1	287	9.60
KQC42043.1	Ceramide synthase component [*Saccharomyces boulardii*]	17.2	27.32	6.67	1	1	1	1	150	6.67
KQC43824.1	Formylglycinamidine-ribonucleotide (FGAM)-synthetase [*Saccharomyces boulardii*]	148.8	27.30	0.66	1	1	1	1	1358	5.27
KQC40651.1	Component of both the SWI/SNF and RSC chromatin remodeling complex [*Saccharomyces boulardii*]	53.8	27.24	1.47	1	1	1	1	477	5.48
KQC41794.1	FAR8p involved in recovery from arrest in response to pheromone [*Saccharomyces boulardii*]	59.2	27.22	2.10	1	1	1	1	523	5.24
KQC42266.1	Core Sm protein Sm D3 [*Saccharomyces boulardii*]	11.2	27.20	8.91	1	1	1	1	101	9.99
KQC41255.1	Vacuolar membrane protein [*Saccharomyces boulardii*]	20.1	27.20	2.72	1	1	1	1	184	5.45
KQC40466.1	Poly(A)RNA binding protein involved in nuclear mRNA export [*Saccharomyces boulardii*]	67.3	27.17	2.67	1	1	1	1	599	9.20
KQC41319.1	hypothetical protein AB282_04180 [*Saccharomyces boulardii*]	10.8	27.14	8.16	1	1	1	1	98	8.97
KQC45173.1	GTPase-activating protein [*Saccharomyces boulardii*]	87.3	27.14	1.34	1	1	1	1	746	5.16
KQC44524.1	Htz1p-binding component of the SWR1 complex [*Saccharomyces boulardii*]	90.4	27.12	1.01	2	1	1	1	795	5.24
KQC45424.1	RCR1p of the ER membrane involved in cell wall chitin deposition [*Saccharomyces boulardii*]	23.9	27.09	6.10	1	1	1	1	213	7.05
KQC44404.1	Type 2C protein phosphatase (PP2C) [*Saccharomyces boulardii*]	47.8	27.05	1.81	2	1	1	1	442	4.98
KQC41606.1	Pseudouridine synthase [*Saccharomyces boulardii*]	45.2	27.03	2.73	1	1	1	1	403	6.58
KQC41097.1	5'-3' exonuclease and flap-endonuclease [*Saccharomyces boulardii*]	115.8	26.94	0.99	1	1	1	1	1006	6.81
KQC40420.1	putative FAD transporter [*Saccharomyces boulardii*]	89.2	26.81	1.39	1	1	1	1	793	7.93
KQC41194.1	Cargo-binding mu subunit of AP-2 [*Saccharomyces boulardii*]	55.5	26.70	1.63	1	1	1	1	491	8.27
KQC44027.1	RNA-dependent ATPase [*Saccharomyces boulardii*]	63.6	26.65	1.06	1	1	1	1	564	9.20
KQC45844.1	Cytoplasmic protein required for sporulation [*Saccharomyces boulardii*]	33.8	26.54	2.06	1	1	1	1	291	5.07
KQC44729.1	Ribosomal lysine methyltransferase [*Saccharomyces boulardii*]	57.3	26.52	2.43	1	1	1	1	494	5.05
KQC43454.1	LSM12p function in RNA processing [*Saccharomyces boulardii*]	21.3	26.30	4.81	1	1	1	1	187	9.39
KQC45027.1	Component of the Rpd3L histone deacetylase complex [*Saccharomyces boulardii*]	33.8	26.28	3.74	1	1	1	1	294	6.54
KQC40695.1	Lipid-binding ER protein, enriched at nucleus-vacuolar junctions (NVJ) [*Saccharomyces boulardii*]	87.2	26.23	1.56	1	1	1	1	770	7.44
KQC41393.1	hypothetical protein AB282_04254 [*Saccharomyces boulardii*]	72.8	26.15	1.27	1	1	1	1	632	5.99
KQC45078.1	Vacuolar membrane antiporter with Ca2+/H+ and K+/H+ exchange activity [*Saccharomyces boulardii*]	44.7	26.00	2.68	1	1	1	1	411	5.40
KQC41938.1	Mitochondrial ribosomal protein of the large subunit [*Saccharomyces boulardii*]	26.0	26.00	4.05	2	1	1	1	222	9.44

KQC41557.1	Kexin, a subtilisin-like protease (proprotein convertase) [*Saccharomyces boulardii*]	89.9	25.84	1.23	1	1	1	1	814	4.98
KQC41882.1	Essential DEAH-box ATP-dependent RNA helicase specific to U3 snoRNP [*Saccharomyces boulardii*]	144.9	25.81	1.42	1	2	2	2	1267	6.39
KQC44562.1	Subunit of the RNA polymerase II mediator complex [*Saccharomyces boulardii*]	159.8	25.81	0.70	1	1	1	1	1420	5.66
KQC40918.1	hypothetical protein AB282_04848 [*Saccharomyces boulardii*]	20.8	25.76	6.18	1	1	1	1	178	10.07
KQC45236.1	TFIID subunit (150 kDa) [*Saccharomyces boulardii*]	159.1	25.68	1.15	3	1	2	2	1387	5.90
KQC43542.1	DNA helicase and DNA-dependent ATPase [*Saccharomyces boulardii*]	81.6	25.63	1.24	1	1	1	1	723	9.72
KOH50996.1	RMD8p Cytosolic protein required for sporulation [*Saccharomyces boulardii*]	75.8	25.54	1.66	1	1	1	1	662	7.28
KQC43099.1	Serine-threonine protein kinase [*Saccharomyces boulardii*]	91.2	25.46	0.74	1	1	1	1	807	8.38
KQC41739.1	Subunit of the transmembrane ring of the nuclear pore complex (NPC) [*Saccharomyces boulardii*]	74.1	25.40	1.53	1	1	1	1	655	8.34
KQC43352.1	Mitochondrial inner membrane protein [*Saccharomyces boulardii*]	22.1	25.32	4.59	1	1	1	1	196	9.58
KQC43080.1	Shuttling pre-60S factor [*Saccharomyces boulardii*]	19.4	25.30	4.57	1	1	1	1	175	10.10
KQC45335.1	hypothetical protein AB282_00097 [*Saccharomyces boulardii*]	30.5	25.29	3.33	1	1	1	1	270	7.90
KQC45684.1	Subunit of the SWI/SNF chromatin remodeling complex [*Saccharomyces boulardii*]	102.6	25.28	1.10	1	1	1	1	906	8.28
KQC42396.1	Mitochondrial ribosomal protein of the large subunit [*Saccharomyces boulardii*]	28.1	25.25	4.74	1	1	1	1	253	9.35
KQC42372.1	S-adenosylmethionine-dependent methyltransferase [*Saccharomyces boulardii*]	29.6	25.23	3.45	1	1	1	1	261	5.20
KQC44042.1	Non-essential regulatory subunit B of protein phosphatase 2A (PP2A) [*Saccharomyces boulardii*]	59.6	25.18	1.71	1	1	1	1	526	6.49
KQC41367.1	Cytosolic inhibitor of vacuolar proteinase B (PRB1) [*Saccharomyces boulardii*]	8.6	25.11	12.00	1	1	1	1	75	6.80
KQC45134.1	Ubiquitin chain assembly factor (E4) [*Saccharomyces boulardii*]	109.8	25.10	1.14	1	1	1	1	961	5.86
KQC44061.1	Vacuolar SNARE protein [*Saccharomyces boulardii*]	36.7	25.05	2.85	1	1	1	1	316	8.05
KQC42034.1	U2-snRNP associated splicing factor [*Saccharomyces boulardii*]	109.9	24.99	0.93	1	1	1	1	971	7.20
KQC41341.1	Low-affinity vacuolar phosphate transporter [*Saccharomyces boulardii*]	99.5	24.61	1.34	1	1	1	1	894	8.02
KQC42958.1	Dihydroxyacid dehydratase [*Saccharomyces boulardii*]	62.8	24.43	2.22	1	1	1	1	585	7.83
KQC44191.1	Mitochondrial RNA polymerase [*Saccharomyces boulardii*]	153.0	24.42	0.81	1	1	1	1	1351	8.85
KQC44716.1	Mitochondrial glyoxalase II [*Saccharomyces boulardii*]	31.3	24.21	4.74	1	1	1	1	274	6.19
KQC44610.1	GTPase activating protein (GAP) for Rho1p [*Saccharomyces boulardii*]	73.6	24.02	2.14	1	1	1	1	654	9.51
KQC42314.1	putative glycoside hydrolase of the spore wall envelope [*Saccharomyces boulardii*]	47.3	23.91	1.42	1	1	1	1	422	7.80
KQC41368.1	Poly (A)+ RNA-binding protein [*Saccharomyces boulardii*]	50.7	23.83	1.77	1	1	1	1	453	5.11
KQC44691.1	Component of the RSC chromatin remodeling complex [*Saccharomyces boulardii*]	101.7	23.80	1.24	1	1	1	1	885	8.03
KQC40604.1	Subunit of vacuolar transporter chaperone (VTC) complex [*Saccharomyces boulardii*]	96.4	23.78	1.56	1	1	1	1	835	7.64

KOH48001.1	GAL11p Subunit of the RNA polymerase II mediator complex [*Saccharomyces boulardii*]	121.2	23.65	0.64	2	1	1	1	1088	9.79
KOH47275.1	MSD1p Mitochondrial aspartyl-tRNA synthetase [*Saccharomyces boulardii*]	75.4	23.62	1.37	2	1	1	1	658	8.72
KQC43507.1	Constituent of 66S pre-ribosomal particle [*Saccharomyces boulardii*]	51.7	23.59	2.21	2	1	1	1	453	9.26
KQC41987.1	Member of the ChAPs family (Chs5p-Arf1p-binding protein) [*Saccharomyces boulardii*]	82.0	23.42	2.35	1	1	1	1	724	5.36
KQC43247.1	Basic leucine zipper (bZIP) transcription factor, in ATF/CREB family [*Saccharomyces boulardii*]	47.2	23.42	3.01	2	1	1	1	432	7.39
KQC43403.1	putative low-affinity copper transporter of the vacuolar membrane [*Saccharomyces boulardii*]	21.6	23.41	3.17	1	1	1	1	189	7.42
KQC43464.1	E1-like protein that activates Urm1p before urmylation [*Saccharomyces boulardii*]	49.3	23.16	2.50	1	1	1	1	440	6.54
KQC43511.1	Subunit of both RNase MRP and nuclear RNase P [*Saccharomyces boulardii*]	32.2	23.10	4.10	1	1	1	1	293	9.66
KQC44931.1	Mitochondrial ribosomal protein of the small subunit [*Saccharomyces boulardii*]	23.4	23.07	5.42	1	1	1	1	203	9.28
KQC43397.1	Integral membrane protein of the early Golgi apparatus and ER [*Saccharomyces boulardii*]	26.3	22.78	5.26	1	1	1	1	228	8.40
KQC42721.1	Subunit of U3-containing Small Subunit (SSU) processome complex [*Saccharomyces boulardii*]	29.6	22.72	4.80	1	1	1	1	250	10.18
KQC41095.1	Nuclear protein that inhibits replication of Brome mosaic virus [*Saccharomyces boulardii*]	47.4	22.57	2.91	1	1	1	1	412	4.83
KQC43151.1	Subunit of the SF3a splicing factor complex [*Saccharomyces boulardii*]	33.0	22.46	6.79	1	1	1	1	280	8.24
KOH47250.1	TAF14p Subunit of TFIID, TFIIF, INO80, SWI/SNF, and NuA3 complexes [*Saccharomyces boulardii*]	50.7	22.42	1.55	2	1	1	1	452	4.61
KQC45068.1	Subunit of the Nup84p subcomplex of the nuclear pore complex (NPC) [*Saccharomyces boulardii*]	83.6	22.29	0.96	1	1	1	1	726	4.86
KQC43722.1	Mitochondrial protein required for assembly of cytochrome bc1 complex [*Saccharomyces boulardii*]	17.5	22.22	6.80	1	1	1	1	147	9.17
KQC40770.1	Subunit of the HDA1 histone deacetylase complex [*Saccharomyces boulardii*]	75.4	22.18	1.07	1	1	1	1	655	6.27
KOH51390.1	POL3p Catalytic subunit of DNA polymerase delta [*Saccharomyces boulardii*]	124.6	22.14	0.82	2	1	1	1	1097	8.07
KQC45675.1	F-Box protein involved in proteasome-dependent degradation of Aah1p [*Saccharomyces boulardii*]	70.5	21.99	1.26	1	1	1	1	637	6.28
KQC40433.1	RIF1p that binds to the Rap1p C-terminus [*Saccharomyces boulardii*]	67.1	21.29	2.92	1	1	1	1	583	5.14
KQC42701.1	hypothetical protein AB282_03129 [*Saccharomyces boulardii*]	46.0	20.93	2.55	1	1	1	1	392	8.91
KQC41064.1	Vacuolar cation channel [*Saccharomyces boulardii*]	77.9	20.82	1.48	1	1	1	1	675	5.38
KQC45734.1	Essential nuclear protein [*Saccharomyces boulardii*]	35.7	20.71	3.59	1	1	1	1	306	5.25
KQC44788.1	Hydrophilic protein involved in ER/Golgi vesicle trafficking [*Saccharomyces boulardii*]	74.6	20.37	4.05	1	1	1	1	666	5.22
KQC43856.1	tRNAHis guanylyltransferase [*Saccharomyces boulardii*]	27.8	20.25	2.53	1	1	1	1	237	8.19
KQC43946.1	hypothetical protein AB282_02202 [*Saccharomyces boulardii*]	15.0	20.10	7.69	1	1	1	1	130	9.44
KQC40600.1	MET12p with MTHFR activity in vitro [*Saccharomyces boulardii*]	73.9	20.08	0.91	1	1	1	1	657	5.36

KQC45468.1	Subunit of the SF3a splicing factor complex [*Saccharomyces boulardii*]	104.2	19.59	0.78	1	1	1	1	899	8.13
