# Supplementary material for: Isolation and Characteristics of Extracellular Vesicles Produced by Probiotics: Yeast Saccharomyces boulardii CNCM I-745 and Bacterium Streptococcus salivarius K12
Source: Probiotics Antimicrob Proteins. 2023 May 20;16(3):936–48. doi: 10.1007/s12602-023-10085-3 (PMC11126510; doi:10.1007/s12602-023-10085-3)
Supplement: Supplementary file 2 — Supplementary file2 (PDF 741 kb) [file 12602_2023_10085_MOESM2_ESM.pdf]

Isolation and characteristics of extracellular vesicles produced by probiotics: yeast *Saccharomyces boulardii* CNCM I-745 and bacterium *Streptococcus salivarius* K12

Kamila Kulig[1], Katarzyna Kowalik[1], Magdalena Surowiec[1,2], Elzbieta Karnas[3], Olga Barczyk-Woznicka[4], Ewa Zuba-Surma[3], Elzbieta Pyza[4], Andrzej Kozik[5], Maria Rapala-Kozik[1], Justyna Karkowska-Kuleta[1*]

[1] Department of Comparative Biochemistry and Bioanalytics, Faculty of Biochemistry, Biophysics and Biotechnology, Jagiellonian University, Kraków, Poland

[2] Doctoral School of Exact and Natural Sciences, Jagiellonian University, Kraków, Poland

[3] Department of Cell Biology, Faculty of Biochemistry, Biophysics and Biotechnology, Jagiellonian University, Kraków, Poland

[4] Department of Cell Biology and Imaging, Institute of Zoology and Biomedical Research, Jagiellonian University, Kraków, Poland

[5] Department of Analytical Biochemistry, Faculty of Biochemistry, Biophysics and Biotechnology, Jagiellonian University, Kraków, Poland

[*]Corresponding author: justyna.karkowska@uj.edu.pl

Supplementary Table 2. Mass spectrometry identification of *Streptococcus salivarius* proteins in EVs. The proteomic analysis of extracellular vesicles was performed with samples prepared with a total amount of proteins equal to 11 µg. After digestion with Trypsin/Lys-C Mix the resulting peptides were analyzed using UltiMate 3000 RSLCnano System coupled with Q-Exactive mass spectrometer with DPV-550 Digital PicoView nanospray source. The obtained lists of peaks were searched against the NCBI protein database with the locally installed MASCOT search engine using the taxonomy restrictions: *Streptococcus salivarius*. The Target Decoy PSM Validator was applied with the maximum false discovery rate (FDR) for peptides set to 0.01. Proteins identified with a score > 20 and a number of identified peptides ≥ 1 are only listed.

Accession number	Description	Molecular mass [kDa]	Score	Coverage	# Proteins	# Unique Peptides	# Peptides	# PSMs	# AAs	calc. pI
WP_002891301.1	KxYKxGKxW signal peptide domain-containing protein [*Streptococcus salivarius*]	133.1	8000.85	72.98	72	26	71	257	1166	5.35
WP_002890946.1	extracellular solute-binding protein [*Streptococcus salivarius*]	44.9	5925.35	75.18	51	6	23	131	415	5.34
WP_002891210.1	GH32 C-terminal domain-containing protein [*Streptococcus salivarius*]	144.8	5889.31	53.24	17	10	53	168	1298	4.98
WP_002891316.1	hypothetical protein [*Streptococcus salivarius*]	118.8	5307.92	53.30	22	48	48	157	1060	5.29
WP_037597470.1	SH3 domain-containing protein [*Streptococcus salivarius*]	92.8	4738.35	62.75	97	1	36	111	851	6.00
WP_084914426.1	GH32 C-terminal domain-containing protein [*Streptococcus salivarius*]	145.2	4599.09	37.22	2	1	38	125	1303	4.93
WP_201552173.1	GH32 C-terminal domain-containing protein [*Streptococcus salivarius*]	145.1	4491.93	37.15	3	1	39	131	1303	4.97
WP_181670414.1	GH32 C-terminal domain-containing protein [*Streptococcus salivarius*]	145.6	4436.89	36.84	2	1	38	118	1303	4.94
HBJ22654.1	hypothetical protein [*Streptococcus salivarius*]	92.7	4413.30	53.47	54	0	33	102	851	6.00
WP_002891318.1	KxYKxGKxW signal peptide domain-containing protein [*Streptococcus salivarius*]	95.5	4334.82	52.85	60	9	44	156	842	5.53
WP_061652463.1	extracellular solute-binding protein [*Streptococcus*]	44.8	3889.79	51.08	4	1	18	90	415	5.34
WP_201711931.1	SH3 domain-containing protein [*Streptococcus salivarius*]	92.7	3667.86	46.06	34	1	27	87	851	5.78
WP_156209578.1	KxYKxGKxW signal peptide domain-containing protein [*Streptococcus salivarius*]	132.7	3350.40	35.06	2	1	39	121	1161	5.45
WP_037597887.1	BMP family protein [*Streptococcus*]	37.3	2977.04	62.36	24	4	16	78	356	5.41
WP_195187351.1	phosphopyruvate hydratase, partial [*Streptococcus salivarius*]	46.2	2626.00	68.93	33	4	20	51	428	4.75
WP_037597995.1	KxYKxGKxW signal peptide domain-containing protein [*Streptococcus*]	122.9	2623.83	52.25	61	43	46	70	1089	5.31
WP_197092241.1	Ig-like domain-containing protein [*Streptococcus salivarius*]	436.1	2500.31	50.68	39	10	42	58	4416	4.44
MBS6885968.1	SH3 domain-containing protein [*Streptococcus salivarius*]	45.3	2483.58	46.17	10	1	12	42	418	8.44
WP_002884208.1	peptide ABC transporter substrate-binding protein [*Streptococcus salivarius*]	71.9	2417.05	57.93	61	2	29	58	656	5.24
WP_002890213.1	elongation factor Tu [*Streptococcus*]	43.8	2301.09	62.06	15	6	20	56	398	4.93
WP_037598293.1	peptide ABC transporter substrate-binding protein [*Streptococcus salivarius*]	72.9	2056.28	51.83	10	1	28	64	656	5.47
MBZ5837697.1	YSIRK signal domain/LPXTG anchor domain surface protein [*Streptococcus salivarius*]	233.7	1863.24	33.74	81	3	41	48	2107	5.00
WP_002884461.1	type I glyceraldehyde-3-phosphate dehydrogenase [*Streptococcus*]	36.0	1858.91	70.24	26	19	19	50	336	5.58
WP_224111213.1	glucosyl transferase, partial [*Streptococcus salivarius*]	81.6	1840.05	52.47	18	24	29	47	730	5.16
WP_002886978.1	peptide ABC transporter substrate-binding protein [*Streptococcus salivarius*]	72.9	1839.44	56.25	39	25	27	49	656	5.36
WP_002889856.1	penicillin-binding protein PBP1A [*Streptococcus salivarius*]	83.9	1837.78	45.03	78	27	27	47	764	5.54
WP_002891330.1	glucosyl transferase [*Streptococcus salivarius*]	107.7	1834.86	46.18	24	27	36	53	955	5.39

WP_002892041.1	DNA-directed RNA polymerase subunit beta [*Streptococcus*]	133.1	1809.39	43.12	36	41	41	66	1192	5.12
EJO16087.1	alpha-C protein [*Streptococcus salivarius* K12]	166.7	1800.18	37.97	120	32	37	47	1633	4.88
WP_037598248.1	YSIRK-type signal peptide-containing protein, partial [*Streptococcus salivarius*]	180.3	1645.70	26.12	31	2	35	42	1654	4.98
WP_155212158.1	G5 domain-containing protein [*Streptococcus salivarius*]	216.9	1593.06	26.69	146	41	43	46	1967	5.25
WP_247922735.1	transporter substrate-binding domain-containing protein [*Streptococcus salivarius*]	28.3	1546.13	52.65	12	1	11	30	264	5.36
WP_002890008.1	glycoside hydrolase family 68 protein [*Streptococcus salivarius*]	104.2	1514.82	34.71	88	23	23	41	971	4.96
WP_002891317.1	KxYKxGKxW signal peptide domain-containing protein [*Streptococcus salivarius*]	68.7	1513.09	44.44	80	25	26	44	621	5.24
WP_002887576.1	elongation factor Tu [*Streptococcus*]	43.8	1422.02	44.97	1	1	13	38	398	5.05
MBS6319243.1	DNA-directed RNA polymerase subunit alpha [*Streptococcus salivarius*]	34.3	1395.30	50.32	17	19	19	39	312	4.94
WP_224111202.1	GBS Bsp-like repeat-containing protein, partial [*Streptococcus salivarius*]	128.3	1332.93	37.55	71	8	29	40	1177	6.61
WP_038675638.1	N4-gp56 family major capsid protein [*Streptococcus salivarius*]	30.2	1323.81	50.89	4	5	14	39	281	4.96
WP_002890936.1	EfeM/EfeO family lipoprotein [*Streptococcus*]	32.4	1295.52	60.69	54	14	14	32	290	4.97
MBS5094002.1	NADP-dependent glyceraldehyde-3-phosphate dehydrogenase [*Streptococcus salivarius*]	50.9	1294.23	55.77	27	20	20	32	477	4.98
WP_037597787.1	GBS Bsp-like repeat-containing protein [*Streptococcus salivarius*]	128.5	1274.69	28.74	104	3	30	36	1183	6.61
WP_002889937.1	amino acid ABC transporter substrate-binding protein [*Streptococcus*]	31.4	1244.09	52.84	22	3	11	31	282	8.31
WP_002886342.1	30S ribosomal protein S2 [*Streptococcus*]	28.3	1233.33	56.47	10	13	13	40	255	5.16
WP_002890793.1	pyruvate kinase [*Streptococcus*]	54.4	1226.03	54.60	26	8	22	36	500	5.06
WP_002891326.1	glucosyl transferase [*Streptococcus salivarius*]	83.9	1208.82	35.55	12	17	21	29	751	5.40
MBS7215133.1	DUF1080 domain-containing protein [*Streptococcus salivarius*]	61.0	1134.69	22.55	1	1	10	34	550	4.97
WP_014633924.1	ABC transporter substrate-binding protein [*Streptococcus*]	41.7	1130.76	57.29	42	19	19	33	391	6.65
WP_195187315.1	F0F1 ATP synthase subunit beta, partial [*Streptococcus salivarius*]	50.3	1121.68	52.05	21	16	17	29	463	4.81
WP_155123969.1	GBS Bsp-like repeat-containing protein [*Streptococcus salivarius*]	142.7	1109.35	23.35	151	0	28	32	1319	6.70
WP_049545960.1	metal ABC transporter substrate-binding protein [*Streptococcus salivarius*]	34.4	1105.68	55.05	48	14	14	41	307	7.85
MBS6973642.1	ABC transporter substrate-binding protein [*Streptococcus salivarius*]	37.4	1094.43	52.07	60	4	15	31	338	5.43
WP_002890136.1	peptidyl-prolyl cis-trans isomerase [*Streptococcus*]	40.8	1059.69	50.79	31	15	15	22	380	5.25
WP_004182359.1	L-lactate dehydrogenase [*Streptococcus*]	35.3	1048.91	49.70	20	8	14	26	328	5.16
WP_002891141.1	phospho-sugar mutase [*Streptococcus salivarius*]	62.8	1040.00	42.31	44	17	17	23	572	4.88
WP_002885626.1	30S ribosomal protein S5 [*Streptococcus*]	17.1	1006.18	60.98	7	10	10	31	164	9.85
WP_037597997.1	KxYKxGKxW signal peptide domain-containing protein [*Streptococcus salivarius*]	72.3	997.06	40.56	65	19	22	26	646	5.21
MBS7214128.1	class B sortase [*Streptococcus salivarius*]	24.0	976.64	68.27	16	12	12	23	208	5.01
MBS4822640.1	DUF5105 domain-containing protein [*Streptococcus salivarius*]	42.5	857.85	51.85	52	16	16	19	378	5.01
WP_002885642.1	30S ribosomal protein S8 [*Streptococcus*]	14.8	822.42	58.33	8	12	12	32	132	9.52
WP_002891098.1	ZinT/AdcA family metal-binding protein [*Streptococcus*]	57.9	807.96	37.35	59	18	18	24	514	5.91
WP_037598508.1	50S ribosomal protein L1 [*Streptococcus*]	24.4	805.61	50.66	10	11	11	27	229	9.23

ACN23005.1	mannose-specific phosphotransferase system component IIAB high [*Streptococcus salivarius*]	36.3	805.29	51.34	29	11	13	24	335	5.49
WP_002884710.1	glucose-1-phosphate adenylyltransferase subunit GlgD [*Streptococcus*]	42.3	790.48	44.71	47	16	16	18	378	5.59
WP_002890247.1	peptidylprolyl isomerase [*Streptococcus salivarius*]	27.8	785.14	59.13	41	13	13	22	252	9.20
WP_073922277.1	ABC transporter ATP-binding protein, partial [*Streptococcus salivarius*]	55.2	764.17	34.91	59	15	15	19	507	5.62
EIC81674.1	Hypothetical protein [*Streptococcus salivarius* PS4]	31.7	760.18	50.35	12	14	14	18	282	5.80
WP_003095623.1	50S ribosomal protein L10 [*Streptococcus*]	17.6	759.42	80.24	14	12	12	17	167	4.84
WP_155123927.1	trypsin-like peptidase domain-containing protein [*Streptococcus*]	41.3	758.16	45.34	32	12	12	17	397	4.84
HBJ22627.1	F0F1 ATP synthase subunit alpha [*Streptococcus salivarius*]	54.6	755.15	43.11	27	16	16	24	501	5.08
WP_002889939.1	MetQ/NlpA family ABC transporter substrate-binding protein [*Streptococcus*]	32.7	753.84	46.33	23	10	10	24	300	5.14
WP_002887066.1	50S ribosomal protein L23 [*Streptococcus*]	10.8	738.48	60.20	6	8	8	29	98	9.63
WP_021151430.1	amino acid ABC transporter substrate-binding protein [*Streptococcus*]	31.9	735.80	41.81	3	1	9	22	287	8.54
WP_253183498.1	substrate-binding domain-containing protein, partial [*Streptococcus salivarius*]	33.4	735.54	66.24	43	1	14	18	314	6.40
WP_037597698.1	KxYKxGKxW signal peptide domain-containing protein [*Streptococcus salivarius*]	176.3	725.08	16.89	118	21	21	23	1599	5.68
RGW04557.1	glucosyltransferase [*Streptococcus salivarius*]	167.7	716.86	18.51	103	23	24	24	1518	5.48
WP_002884537.1	elongation factor G [*Streptococcus*]	76.6	714.51	34.05	11	16	16	20	693	4.92
WP_143567035.1	N4-gp56 family major capsid protein [*Streptococcus salivarius*]	30.1	711.71	28.11	2	1	10	25	281	5.01
WP_211180922.1	ABC transporter substrate-binding protein [*Streptococcus salivarius*]	35.6	707.94	61.56	10	1	14	18	333	8.47
MBS6319482.1	phenylalanine--tRNA ligase subunit beta [*Streptococcus salivarius*]	86.9	693.20	25.53	89	16	16	18	799	4.84
WP_156246919.1	YSIRK-type signal peptide-containing protein [*Streptococcus salivarius*]	558.5	687.53	9.58	145	1	19	20	5092	5.08
MBS5181122.1	PTS transporter subunit IIBC [*Streptococcus salivarius*]	77.7	680.62	24.79	76	16	16	19	726	6.68
WP_002889982.1	PTS sugar transporter subunit IIB [*Streptococcus salivarius*]	35.5	667.50	56.97	19	15	16	24	330	5.15
WP_002884746.1	30S ribosomal protein S7 [*Streptococcus*]	17.7	658.75	66.67	6	11	11	22	156	10.17
MBS4923661.1	signal recognition particle protein [*Streptococcus salivarius*]	57.8	650.77	32.12	42	14	14	17	520	6.51
WP_253184019.1	transporter substrate-binding domain-containing protein [*Streptococcus salivarius*]	29.3	648.08	59.62	29	15	15	18	265	6.18
MBS5181476.1	alpha-ketoacid dehydrogenase subunit beta [*Streptococcus salivarius*]	35.8	647.57	40.36	34	15	15	18	332	5.02
WP_002884322.1	type I glutamate--ammonia ligase [*Streptococcus*]	50.1	640.07	34.45	14	13	13	17	447	4.96
WP_037597506.1	SH3 domain-containing protein [*Streptococcus salivarius*]	37.4	636.73	36.87	76	10	10	13	358	8.00
MBS6732218.1	sucrose-specific PTS transporter subunit IIBC [*Streptococcus salivarius*]	66.9	631.37	15.96	41	6	6	14	633	5.24
MBS5180052.1	sn-glycerol-3-phosphate ABC transporter ATP-binding protein UgpC [*Streptococcus salivarius*]	42.0	616.00	41.27	43	12	12	16	378	6.02
WP_110980828.1	DAK2 domain-containing protein [*Streptococcus*]	58.0	607.24	28.31	61	11	11	13	544	4.48
MBS6120854.1	DUF1002 domain-containing protein [*Streptococcus salivarius*]	32.4	596.21	40.73	49	10	10	16	302	4.94
WP_002885816.1	fructose-bisphosphate aldolase [*Streptococcus*]	31.5	594.49	43.00	13	9	9	15	293	4.91
WP_002883819.1	uracil phosphoribosyltransferase [*Streptococcus*]	23.0	588.04	57.42	8	9	9	17	209	5.67
WP_002886397.1	molecular chaperone DnaK [*Streptococcus*]	64.7	578.04	29.98	32	15	15	15	607	4.75

MBS6761793.1	D-alanyl-lipoteichoic acid biosynthesis protein DltD [*Streptococcus salivarius*]	48.4	571.94	32.70	66	11	11	13	422	9.14
WP_247924669.1	phage portal protein [*Streptococcus salivarius*]	57.0	568.05	39.64	10	15	15	16	502	4.88
WP_073689733.1	ABC transporter substrate-binding protein/permease, partial [*Streptococcus salivarius*]	53.8	544.21	29.80	54	9	12	13	490	7.43
WP_002890932.1	dihydrolipoyl dehydrogenase [*Streptococcus salivarius*]	62.0	539.97	30.43	113	13	15	15	585	4.93
WP_002890636.1	LemA family protein [*Streptococcus*]	20.7	535.03	52.15	22	7	8	14	186	5.71
MBS6760418.1	PTS mannose/fructose/sorbose transporter family subunit IID [*Streptococcus salivarius*]	33.2	533.19	29.28	31	7	7	15	304	9.39
MBS6760798.1	hypothetical protein [*Streptococcus salivarius*]	62.6	527.34	28.52	78	13	13	14	575	4.91
WP_002890555.1	hypothetical protein [*Streptococcus salivarius*]	22.1	526.42	41.18	47	5	5	8	204	5.31
WP_002890004.1	cation-transporting P-type ATPase [*Streptococcus salivarius*]	101.8	525.19	17.93	83	11	11	11	926	5.50
WP_002887056.1	50S ribosomal protein L6 [*Streptococcus*]	19.5	525.11	58.99	14	8	8	13	178	9.57
MBF1719103.1	ABC transporter ATP-binding protein [*Streptococcus salivarius*]	34.9	521.10	39.94	39	10	10	13	308	6.48
HBJ24143.1	septation ring formation regulator EzrA [*Streptococcus salivarius*]	66.4	520.96	21.08	54	12	12	14	574	4.77
WP_002890299.1	alanine dehydrogenase [*Streptococcus salivarius*]	38.7	520.37	40.54	63	12	12	15	370	5.96
MBS5421566.1	ABC transporter ATP-binding protein [*Streptococcus salivarius*]	39.9	519.33	36.57	43	11	11	16	361	5.03
WP_002891327.1	glucosyl transferase [*Streptococcus salivarius*]	107.4	512.22	16.39	100	6	13	14	958	5.08
MBS6531561.1	thiamine pyrophosphate-dependent dehydrogenase E1 component subunit alpha [*Streptococcus salivarius*]	39.3	503.47	31.93	21	9	9	13	357	5.20
MBS7214411.1	triose-phosphate isomerase [*Streptococcus salivarius*]	26.5	501.57	39.68	16	3	7	12	252	4.75
MBS6530050.1	penicillin-binding protein PBP2A [*Streptococcus salivarius*]	84.8	496.66	19.61	68	14	14	16	775	5.33
AEJ54136.1	urease alpha subunit [*Streptococcus salivarius* 57.I]	61.3	490.02	24.12	19	11	11	13	568	5.15
MBF1718705.1	D-alanyl-D-alanine carboxypeptidase family protein [*Streptococcus salivarius*]	29.4	486.28	50.94	51	7	7	11	265	5.96
WP_002890381.1	ATP-dependent Clp protease ATP-binding subunit [*Streptococcus salivarius*]	83.9	478.62	19.97	66	12	12	13	756	5.47
WP_002884739.1	phosphoglycerate kinase [*Streptococcus*]	42.0	473.55	34.34	23	11	11	14	399	5.05
WP_002889802.1	RIP metalloprotease RseP [*Streptococcus salivarius*]	45.7	470.29	26.67	48	12	12	14	420	6.29
WP_002891535.1	matrixin family metalloprotease [*Streptococcus salivarius*]	48.0	469.58	24.55	79	8	8	13	440	9.17
WP_002885755.1	50S ribosomal protein L3 [*Streptococcus*]	22.4	468.98	28.85	3	5	5	11	208	10.07
WP_002884784.1	GTP-sensing pleiotropic transcriptional regulator CodY [*Streptococcus*]	28.8	468.04	46.36	7	9	9	12	261	4.75
WP_002885709.1	50S ribosomal protein L17 [*Streptococcus*]	14.4	465.83	43.75	6	5	5	9	128	9.86
MBS6890365.1	large conductance mechanosensitive channel protein MscL [*Streptococcus salivarius*]	13.3	464.16	22.22	20	2	3	7	126	5.15
WP_155123925.1	30S ribosomal protein S3 [*Streptococcus*]	23.2	461.40	51.92	9	9	9	13	208	9.64
WP_002885833.1	30S ribosomal protein S4 [*Streptococcus*]	23.0	459.36	47.78	6	13	13	16	203	10.07
WP_002885764.1	ketol-acid reductoisomerase [*Streptococcus*]	37.3	455.93	30.29	19	6	9	12	340	4.87
WP_002883761.1	ABC transporter ATP-binding protein [*Streptococcus*]	25.5	451.86	38.56	15	10	10	12	236	5.71
WP_084913400.1	ATP-dependent Clp protease ATP-binding subunit ClpX, partial [*Streptococcus salivarius*]	38.8	451.19	27.35	22	9	9	10	351	4.68

WP_002890666.1	cell division protein FtsA [*Streptococcus*]	49.9	445.39	36.90	23	12	12	16	458	4.75
WP_002889882.1	endolytic transglycosylase MltG [*Streptococcus salivarius*]	72.9	444.40	20.52	77	12	12	14	658	5.90
WP_002890886.1	LCP family protein [*Streptococcus salivarius*]	53.6	443.65	26.13	64	10	10	11	486	8.62
WP_002890667.1	cell division protein FtsZ [*Streptococcus salivarius*]	46.3	443.24	30.00	28	10	10	12	440	4.65
WP_002891304.1	M13 family metallopeptidase [*Streptococcus salivarius*]	76.0	440.67	27.87	78	16	16	16	671	5.54
MBS6733013.1	LTA synthase family protein [*Streptococcus salivarius*]	66.9	438.80	23.70	68	14	14	15	595	6.00
MBS6732046.1	glucose-1-phosphate adenylyltransferase [*Streptococcus salivarius*]	41.6	435.47	25.53	40	8	8	9	380	4.92
WP_247914992.1	ABC transporter permease, partial [*Streptococcus salivarius*]	60.7	434.65	22.38	65	11	11	12	572	7.06
WP_002891489.1	amino acid ABC transporter ATP-binding protein [*Streptococcus*]	27.1	431.28	28.46	23	5	6	14	246	4.86
WP_002886286.1	ribose-phosphate diphosphokinase [*Streptococcus*]	35.1	429.38	24.92	15	6	6	10	321	5.67
WP_002884894.1	capsular polysaccharide biosynthesis protein [*Streptococcus*]	25.5	428.62	37.39	19	8	8	10	230	5.36
MBS5247515.1	cell division protein FtsZ [*Streptococcus salivarius*]	35.9	414.83	46.36	1	10	10	10	343	4.74
WP_002890189.1	aminoacyltransferase [*Streptococcus salivarius*]	45.2	407.90	27.62	57	11	11	12	391	8.84
WP_002886374.1	50S ribosomal protein L13 [*Streptococcus*]	16.2	407.28	52.03	10	6	6	13	148	9.91
WP_002889851.1	aminopeptidase C [*Streptococcus*]	50.3	405.85	37.75	69	15	15	16	445	5.03
WP_002890570.1	YtxH domain-containing protein [*Streptococcus*]	16.1	404.69	60.27	24	2	9	14	146	4.60
MBS5246263.1	metal ABC transporter ATP-binding protein [*Streptococcus salivarius*]	27.1	400.77	33.88	36	7	7	10	242	8.66
WP_002891173.1	class A sortase [*Streptococcus salivarius*]	28.0	390.24	25.30	37	5	5	7	253	8.68
WP_257214045.1	hypothetical protein [*Streptococcus salivarius*]	22.4	390.06	39.00	10	7	7	8	200	9.03
WP_002886343.1	translation elongation factor Ts [*Streptococcus*]	37.3	386.80	31.79	12	9	9	10	346	4.86
WP_155124383.1	PDZ domain-containing protein [*Streptococcus salivarius*]	36.6	385.62	31.07	78	9	9	11	338	7.21
MBS6731602.1	30S ribosome-binding factor RbfA [*Streptococcus salivarius*]	13.5	384.58	41.03	10	6	6	11	117	8.85
WP_037597852.1	(S)-acetoin forming diacetyl reductase [*Streptococcus salivarius*]	26.4	378.01	34.65	29	8	8	9	254	5.15
MBS6890062.1	tyrosine-protein kinase [*Streptococcus salivarius*]	26.8	377.64	36.99	33	8	8	10	246	8.98
MBS4923624.1	glucose-6-phosphate isomerase [*Streptococcus salivarius*]	49.9	377.12	23.83	29	10	10	11	449	4.98
MCB6443278.1	50S ribosomal protein L5 [*Streptococcus salivarius*]	9.5	375.47	23.17	1	1	2	11	82	5.52
WP_002885711.1	50S ribosomal protein L14 [*Streptococcus*]	13.1	375.43	38.52	43	6	6	11	122	10.27
MBS6732724.1	DUF3862 domain-containing protein [*Streptococcus salivarius*]	22.3	375.05	47.57	28	9	9	9	206	8.98
WP_002892304.1	hypothetical protein [*Streptococcus salivarius*]	19.6	374.17	37.23	62	2	5	6	188	6.00
WP_002883717.1	PTS system mannose/fructose/sorbose family transporter subunit IID [*Streptococcus*]	33.4	373.11	20.13	20	7	7	12	303	9.22
MBS6890701.1	6-phosphofructokinase [*Streptococcus salivarius*]	35.7	373.10	29.79	32	9	9	10	339	5.14
WP_002883786.1	ribosome-associated translation inhibitor RaiA [*Streptococcus*]	21.0	370.81	43.96	9	7	7	8	182	5.19
MBS4922978.1	phenylalanine--tRNA ligase subunit alpha [*Streptococcus salivarius*]	39.2	367.95	30.26	45	10	10	11	347	5.74
WP_037597789.1	FTR1 family iron permease [*Streptococcus salivarius*]	61.7	365.23	16.76	96	9	9	10	561	7.66
WP_002891100.1	C69 family dipeptidase [*Streptococcus*]	52.7	359.39	21.29	56	1	10	11	465	5.03
WP_002890539.1	alpha-ketoacid dehydrogenase subunit beta [*Streptococcus*]	37.3	353.68	39.07	21	12	12	12	343	4.98
WP_037597419.1	methionine ABC transporter ATP-binding protein [*Streptococcus salivarius*]	38.6	352.90	31.27	46	9	9	10	355	5.83

WP_073922275.1	phosphate signaling complex protein PhoU, partial [*Streptococcus salivarius*]	24.7	346.97	39.35	27	8	8	9	216	5.22
MBF1717846.1	catabolite control protein A [*Streptococcus salivarius*]	36.7	342.86	26.73	32	8	8	8	333	5.77
WP_002887063.1	50S ribosomal protein L22 [*Streptococcus*]	12.4	342.13	40.35	5	5	5	8	114	11.02
CCB96062.1	penicillin-binding protein 2X [*Streptococcus salivarius* JIM8777]	78.4	341.86	15.10	79	11	11	12	722	5.43
MBS7053832.1	1,4-alpha-glucan branching protein GlgB [*Streptococcus salivarius*]	73.3	340.48	15.18	50	10	10	12	626	5.44
WP_214252643.1	GBS Bsp-like repeat-containing protein [*Streptococcus salivarius*]	130.6	338.63	7.89	181	0	10	10	1204	7.01
MBS4823153.1	hypothetical protein [*Streptococcus salivarius*]	41.2	337.40	25.14	45	8	8	8	358	5.30
WP_231605992.1	Ig-like domain-containing protein [*Streptococcus salivarius*]	129.4	336.56	53.20	53	1	8	10	1312	4.54
HBJ24369.1	UDP-N-acetylglucosamine--N-acetylmuramyl-(pentapeptide) pyrophosphoryl-undecaprenol N-acetylglucosamine transferase [*Streptococcus salivarius*]	39.6	331.75	26.69	55	7	7	9	356	8.16
MBS4924066.1	homoserine dehydrogenase [*Streptococcus salivarius*]	40.5	328.58	25.47	28	7	7	8	373	4.89
WP_195917845.1	hypothetical protein [*Streptococcus salivarius*]	20.1	325.18	42.70	8	6	6	9	185	4.94
WP_002889557.1	rod shape-determining protein MreC [*Streptococcus salivarius*]	30.3	324.59	25.00	40	5	5	6	276	7.24
WP_008535049.1	hypothetical protein [*Streptococcus*]	21.9	311.99	34.95	6	5	5	7	186	6.11
MBS4924105.1	3-oxoacyl-[acyl-carrier-protein] reductase [*Streptococcus salivarius*]	25.8	310.90	33.20	22	7	7	8	244	5.48
WP_002890254.1	bifunctional pyr operon transcriptional regulator/uracil phosphoribosyltransferase PyrR [*Streptococcus*]	19.5	309.20	35.26	10	6	6	7	173	5.24
AIY21692.1	dihydroxy-acid dehydratase [*Streptococcus salivarius*]	59.8	307.06	15.17	54	8	8	8	567	5.12
WP_002883723.1	ABC transporter ATP-binding protein [*Streptococcus*]	27.8	306.00	33.46	23	9	9	12	254	5.14
WP_095559509.1	amino acid ABC transporter permease [*Streptococcus*]	27.0	305.99	22.98	38	5	5	6	248	8.29
WP_002887505.1	50S ribosomal protein L4 [*Streptococcus*]	22.2	303.79	39.13	5	1	7	9	207	9.85
MBS7215085.1	hypothetical protein [*Streptococcus salivarius*]	18.0	302.81	45.91	14	8	8	11	159	8.98
HBJ22629.1	ATP synthase F0 subunit B [*Streptococcus salivarius*]	18.0	302.48	43.03	13	7	7	8	165	5.05
WP_037598539.1	PTS system trehalose-specific EIIBC component [*Streptococcus salivarius*]	70.5	300.11	14.87	58	9	9	12	659	5.29
WP_247914929.1	DUF421 domain-containing protein, partial [*Streptococcus salivarius*]	18.4	298.15	43.29	41	5	5	6	164	5.30
MBS6120303.1	FAD-dependent oxidoreductase [*Streptococcus salivarius*]	30.0	297.99	28.32	64	6	6	7	279	5.73
WP_002892324.1	SAP domain-containing protein [*Streptococcus*]	25.1	293.40	38.97	1	7	7	8	213	9.36
WP_002890102.1	ABC transporter ATP-binding protein/permease [*Streptococcus salivarius*]	67.0	293.18	18.21	80	8	8	9	604	6.10
WP_004182597.1	signal peptidase I [*Streptococcus salivarius*]	20.9	292.30	43.24	20	7	8	11	185	8.24
WP_002889798.1	preprotein translocase subunit YajC [*Streptococcus*]	12.3	291.46	53.57	25	6	6	10	112	4.64
WP_002892057.1	hypothetical protein [*Streptococcus salivarius*]	36.0	291.36	28.31	50	9	9	9	325	8.51
WP_002890526.1	DUF4300 family protein [*Streptococcus salivarius*]	31.5	284.21	18.64	76	6	6	8	279	7.23
MBS4822285.1	signal peptidase I [*Streptococcus salivarius*]	24.1	284.04	31.88	35	8	8	10	207	9.06
MBS4923069.1	alcohol dehydrogenase AdhP [*Streptococcus salivarius*]	35.7	280.33	22.71	50	6	6	7	339	5.12
WP_002886110.1	50S ribosomal protein L7/L12 [*Streptococcus*]	12.3	278.78	79.51	19	8	8	9	122	4.53
WP_201710952.1	ribonuclease J, partial [*Streptococcus salivarius*]	37.2	278.46	20.06	51	6	6	7	334	9.06

MBS6530985.1	class 1b ribonucleoside-diphosphate reductase subunit alpha [*Streptococcus salivarius*]	81.5	273.38	14.46	33	10	10	11	719	5.49
WP_002885597.1	50S ribosomal protein L21 [*Streptococcus*]	11.2	271.99	41.35	6	4	4	7	104	9.86
WP_049544183.1	50S ribosomal protein L20, partial [*Streptococcus salivarius*]	10.3	271.90	46.67	14	4	4	6	90	10.24
WP_002889684.1	LPKTxAVK-anchored surface protein [*Streptococcus*]	14.2	270.57	37.96	10	4	4	6	137	6.01
WP_002895436.1	L-lactate dehydrogenase [*Streptococcus*]	35.3	263.86	17.68	1	1	7	9	328	5.30
WP_002891009.1	iron-siderophore ABC transporter substrate-binding protein [*Streptococcus salivarius*]	36.8	260.32	21.74	75	5	5	6	322	5.00
MBS7214219.1	universal stress protein [*Streptococcus salivarius*]	16.5	260.06	40.82	6	6	6	6	147	5.60
WP_002885849.1	50S ribosomal protein L15 [*Streptococcus*]	15.4	258.45	43.15	12	5	5	8	146	10.49
MBS7055454.1	hypothetical protein [*Streptococcus salivarius*]	7.6	257.85	21.13	39	2	2	6	71	4.88
MBS7215169.1	penicillin-binding protein PBP1B [*Streptococcus salivarius*]	77.0	257.14	10.69	94	7	7	8	711	5.49
WP_037597754.1	ribose-5-phosphate isomerase RpiA [*Streptococcus salivarius*]	24.3	248.59	38.05	31	8	8	9	226	5.07
WP_002884509.1	phosphate acetyltransferase [*Streptococcus salivarius*]	35.4	243.87	25.99	62	7	7	9	327	5.49
MBS6973411.1	ATP-grasp domain-containing protein [*Streptococcus salivarius*]	44.7	237.74	19.12	39	6	6	7	387	4.97
HBJ23453.1	acetolactate synthase AlsS [*Streptococcus salivarius*]	61.1	236.95	20.25	52	10	10	10	558	5.05
MBF1719222.1	30S ribosomal protein S9 [*Streptococcus salivarius*]	13.7	236.18	28.57	8	5	5	9	126	10.49
MBS5422532.1	acetate kinase [*Streptococcus salivarius*]	43.2	235.24	24.94	30	8	8	9	397	5.08
WP_002890017.1	CBS domain-containing protein [*Streptococcus*]	24.1	233.52	28.31	35	6	6	6	219	4.97
WP_247914978.1	FtsQ-type POTRA domain-containing protein, partial [*Streptococcus salivarius*]	28.9	231.62	19.84	72	5	6	8	257	9.73
WP_073686523.1	serine hydroxymethyltransferase, partial [*Streptococcus salivarius*]	40.0	231.35	27.35	55	9	9	9	373	5.90
MBS6760324.1	redox-regulated ATPase YchF [*Streptococcus salivarius*]	41.2	230.99	22.10	17	7	7	7	371	4.82
MBS6119832.1	30S ribosomal protein S12 [*Streptococcus salivarius*]	14.9	230.17	25.19	3	5	5	6	135	11.66
MBS5422237.1	PepSY domain-containing protein [*Streptococcus salivarius*]	22.0	225.25	34.63	61	4	4	5	205	5.43
WP_002886432.1	amino acid ABC transporter ATP-binding protein [*Streptococcus*]	27.5	224.91	32.93	16	7	7	8	246	5.44
WP_002885820.1	30S ribosomal protein S13 [*Streptococcus*]	13.4	223.80	47.11	5	8	8	10	121	10.52
WP_002887048.1	adenylate kinase [*Streptococcus*]	23.5	220.25	33.96	25	5	5	5	212	4.86
WP_002890205.1	F0F1 ATP synthase subunit delta [*Streptococcus*]	20.3	220.25	27.53	26	5	5	8	178	4.97
WP_002885763.1	UTP--glucose-1-phosphate uridylyltransferase GalU [*Streptococcus salivarius*]	33.7	219.10	34.87	22	7	7	7	304	5.48
WP_002891752.1	DUF6287 domain-containing protein [*Streptococcus salivarius*]	37.2	218.47	14.86	65	4	4	4	350	4.91
WP_002883618.1	30S ribosomal protein S15 [*Streptococcus*]	10.5	218.39	30.34	6	4	4	10	89	10.13
WP_037597274.1	phosphoribosylformylglycinamidine cyclo-ligase [*Streptococcus salivarius*]	36.4	218.12	13.53	34	4	4	4	340	4.86
WP_002885567.1	ribosome recycling factor [*Streptococcus*]	20.5	217.55	29.73	10	5	5	6	185	5.80
MBS7056032.1	30S ribosomal protein S19 [*Streptococcus salivarius*]	10.6	214.71	35.87	7	4	4	9	92	10.04
WP_002885874.1	50S ribosomal protein L24 [*Streptococcus*]	10.8	214.38	47.52	4	4	4	6	101	9.91
WP_002883489.1	50S ribosomal protein L19 [*Streptococcus*]	13.2	213.80	48.70	4	6	6	10	115	10.78
WP_002885729.1	50S ribosomal protein L9 [*Streptococcus*]	17.0	211.84	36.84	7	6	6	7	152	9.54

MBS6973123.1	trigger factor [*Streptococcus salivarius*]	46.6	211.68	15.69	33	6	6	7	427	4.51
MBS6119899.1	ABC transporter ATP-binding protein [*Streptococcus salivarius*]	44.9	210.87	18.20	21	6	6	7	401	5.01
WP_247916114.1	DUF5067 domain-containing protein, partial [*Streptococcus salivarius*]	16.7	209.98	33.78	36	5	5	5	148	6.60
WP_002891590.1	ABC transporter ATP-binding protein [*Streptococcus*]	27.0	209.77	25.00	6	5	5	5	244	7.78
MBS6973149.1	Fe-S cluster assembly protein SufD [*Streptococcus salivarius*]	45.9	209.21	25.00	39	9	9	9	420	5.02
MBS6319368.1	CapA family protein [*Streptococcus salivarius*]	45.6	208.86	17.28	59	6	6	6	405	5.63
WP_002892198.1	tryptophan--tRNA ligase [*Streptococcus salivarius*]	38.3	203.28	23.24	32	6	6	6	340	5.74
MBS6530565.1	hypothetical protein [*Streptococcus salivarius*]	14.5	202.34	31.78	6	4	4	4	129	4.94
WP_247922426.1	C69 family dipeptidase, partial [*Streptococcus salivarius*]	30.7	201.31	19.55	2	1	6	7	266	6.28
AEJ52454.1	signaling protein [*Streptococcus salivarius* 57.I]	68.2	195.43	7.35	38	4	4	4	612	5.15
MBS7054012.1	DEAD/DEAH box helicase [*Streptococcus salivarius*]	58.5	195.11	11.30	48	6	6	6	522	9.60
HBJ22662.1	oligoendopeptidase F [*Streptococcus salivarius*]	69.0	194.97	7.65	49	3	3	3	601	4.91
HBJ24030.1	butanediol dehydrogenase [*Streptococcus salivarius*]	37.5	192.51	15.56	18	4	4	5	347	5.01
WP_002885773.1	50S ribosomal protein L30 [*Streptococcus*]	6.4	190.63	41.67	4	2	2	4	60	10.11
WP_142236144.1	Na/Pi cotransporter family protein, partial [*Streptococcus salivarius*]	31.9	190.29	27.27	51	8	8	9	286	5.21
MBS4923064.1	amino acid ABC transporter ATP-binding protein [*Streptococcus salivarius*]	23.3	186.98	22.49	31	4	4	4	209	5.59
MBS7213534.1	CHAP domain-containing protein [*Streptococcus salivarius*]	50.5	186.56	8.68	16	3	3	3	461	4.72
WP_002885648.1	50S ribosomal protein L11 [*Streptococcus*]	14.8	185.53	36.88	2	1	5	6	141	9.28
WP_002884297.1	DUF4230 domain-containing protein [*Streptococcus*]	22.4	185.15	35.64	14	6	6	6	202	8.97
WP_002892302.1	SH3 domain-containing protein [*Streptococcus salivarius*]	67.6	183.48	7.64	14	3	3	3	615	6.06
WP_155214116.1	dTDP-glucose 4,6-dehydratase, partial [*Streptococcus salivarius*]	33.9	182.36	18.42	27	5	5	6	304	6.11
WP_002885810.1	50S ribosomal protein L18 [*Streptococcus*]	12.9	180.80	37.29	4	3	3	5	118	10.40
AEJ52484.1	aspartyl-tRNA synthetase [*Streptococcus salivarius* 57.I]	65.3	180.71	11.23	58	5	5	5	579	4.97
WP_230311018.1	LPXTG cell wall anchor domain-containing protein, partial [*Streptococcus salivarius*]	80.9	180.17	4.25	7	1	2	2	776	4.58
MBS4924007.1	Asp-tRNA(Asn)/Glu-tRNA(Gln) amidotransferase subunit GatB [*Streptococcus salivarius*]	50.7	179.30	14.76	44	6	6	7	454	5.14
WP_224111234.1	accessory Sec-dependent serine-rich glycoprotein adhesin, partial [*Streptococcus salivarius*]	65.5	177.55	6.36	19	3	3	4	629	8.06
RGW04238.1	Fe-S cluster assembly ATPase SufC [*Streptococcus salivarius*]	28.2	176.09	22.66	19	6	6	6	256	4.61
MBS6732012.1	M1 family metallopeptidase [*Streptococcus salivarius*]	96.1	172.44	7.92	93	6	6	6	846	4.72
WP_247915120.1	DeoD-type purine-nucleoside phosphorylase, partial [*Streptococcus salivarius*]	17.4	170.40	13.29	36	2	2	3	158	6.18
MBS7055344.1	hypothetical protein [*Streptococcus salivarius*]	42.5	164.83	18.80	85	8	8	8	367	6.90
WP_247914609.1	peptide chain release factor 3, partial [*Streptococcus salivarius*]	57.2	163.63	11.09	27	4	4	4	505	5.07
WP_002889980.1	PTS mannose/fructose/sorbose transporter subunit IIC [*Streptococcus*]	27.3	163.10	17.71	18	3	3	3	271	5.14
CCB92703.1	biotin carboxylase [*Streptococcus salivarius* CCHSS3]	49.3	161.11	15.52	24	7	7	7	451	5.00
WP_002885617.1	NAD(P)H-dependent oxidoreductase [*Streptococcus*]	22.3	160.26	25.00	19	5	5	6	200	5.60
MBS6530211.1	ribosome assembly RNA-binding protein YhbY [*Streptococcus salivarius*]	11.9	158.67	26.67	14	3	3	6	105	9.67

MBS4922903.1	GTPase Era [*Streptococcus salivarius*]	34.2	157.05	13.71	23	4	4	5	299	6.47
WP_002890971.1	thiol peroxidase [*Streptococcus salivarius*]	18.2	156.85	31.10	38	5	5	5	164	4.61
AIY21333.1	50S ribosomal protein L31 type B [*Streptococcus salivarius*]	9.4	154.60	26.25	6	2	2	3	80	8.43
MBS6733391.1	4-hydroxy-tetrahydrodipicolinate synthase [*Streptococcus salivarius*]	17.5	151.07	19.38	32	2	2	3	160	4.84
MBS4822467.1	asparagine--tRNA ligase [*Streptococcus salivarius*]	50.9	149.68	10.04	32	5	5	5	448	5.06
WP_037598512.1	2,3,4,5-tetrahydropyridine-2,6-dicarboxylate N-acetyltransferase [*Streptococcus*]	24.1	149.30	16.81	22	4	4	5	232	4.89
WP_002890242.1	glycine--tRNA ligase subunit beta [*Streptococcus salivarius*]	74.4	148.13	8.41	88	5	5	5	678	4.77
MBS6120684.1	ATP-binding cassette domain-containing protein [*Streptococcus salivarius*]	22.3	147.41	17.09	26	3	3	3	199	5.27
MBS6531569.1	lipocalin/fatty acid-binding family protein [*Streptococcus salivarius*]	14.9	146.50	36.69	40	3	3	3	139	4.60
WP_142236201.1	2,3-bisphosphoglycerate-dependent phosphoglycerate mutase, partial [*Streptococcus salivarius*]	10.2	146.47	55.56	7	1	5	6	90	4.78
MBS4822949.1	ABC transporter permease [*Streptococcus salivarius*]	34.6	146.08	15.91	29	5	5	5	308	9.36
WP_002890656.1	DNA starvation/stationary phase protection protein [*Streptococcus*]	19.0	144.06	16.18	7	2	2	3	173	4.58
WP_002889931.1	cobalt transporter CbiM [*Streptococcus salivarius*]	35.1	141.80	11.69	30	3	3	3	325	8.48
WP_224111200.1	hypothetical protein [*Streptococcus salivarius*]	53.6	140.27	10.99	87	5	5	5	464	9.55
WP_073690584.1	DUF4300 family protein [*Streptococcus salivarius*]	32.4	139.39	14.80	54	4	4	4	277	8.50
KXU59230.1	30S ribosomal protein S11 [*Streptococcus salivarius*]	12.3	137.63	29.66	6	3	3	3	118	10.42
WP_173014721.1	ABC transporter permease subunit, partial [*Streptococcus salivarius*]	20.6	137.33	32.28	77	3	3	4	189	7.12
WP_247922559.1	transcription termination factor NusA, partial [*Streptococcus salivarius*]	28.7	137.28	20.88	30	5	5	5	249	5.08
MBS6891385.1	16S rRNA (adenine(1518)-N(6)/adenine(1519)-N(6))-dimethyltransferase RsmA [*Streptococcus salivarius*]	32.7	137.03	10.69	42	3	3	4	290	5.73
WP_002886694.1	MarR family transcriptional regulator [*Streptococcus salivarius*]	16.6	134.87	35.66	41	6	6	7	143	8.79
WP_253184712.1	LPXTG cell wall anchor domain-containing protein, partial [*Streptococcus salivarius*]	67.6	134.87	6.43	22	2	2	2	638	4.74
WP_002890025.1	manganese-dependent inorganic pyrophosphatase [*Streptococcus salivarius*]	33.7	134.84	16.45	51	4	4	4	310	4.51
MBS5247019.1	2-oxo acid dehydrogenase subunit E2 [*Streptococcus salivarius*]	43.9	134.16	10.02	55	4	4	4	409	5.07
HBJ23624.1	zinc ABC transporter ATP-binding protein [*Streptococcus salivarius*]	22.9	134.07	9.95	15	3	3	3	201	9.52
WP_247915004.1	Fe-S cluster assembly protein SufB, partial [*Streptococcus salivarius*]	45.1	131.76	10.67	17	4	4	4	403	5.07
WP_000960950.1	50S ribosomal protein L16 [*Streptococcus*]	15.4	129.63	18.98	6	3	3	5	137	10.65
MBS6732077.1	tRNA 2-thiouridine(34) synthase MnmA [*Streptococcus salivarius*]	41.7	129.53	9.92	36	3	3	3	373	5.43
MBS7055411.1	LysM domain-containing protein [*Streptococcus salivarius*]	30.4	128.32	9.93	70	3	3	3	282	4.97
WP_155164685.1	YpmS family protein [*Streptococcus salivarius*]	19.6	127.63	18.54	50	3	3	3	178	9.50
WP_002891651.1	DUF536 domain-containing protein [*Streptococcus salivarius*]	20.5	127.26	19.10	5	3	3	3	178	4.89
WP_002883695.1	YneF family protein [*Streptococcus*]	9.4	125.94	34.94	6	3	3	5	83	10.13
HBJ23662.1	nicotinate phosphoribosyltransferase [*Streptococcus salivarius*]	54.4	125.60	10.54	59	4	4	4	484	5.25
MBS7214966.1	DEAD/DEAH box helicase [*Streptococcus salivarius*]	36.8	124.16	6.79	16	1	1	1	324	6.90
WP_004182179.1	polyribonucleotide nucleotidyltransferase [*Streptococcus salivarius*]	80.9	122.96	6.21	67	4	4	4	741	5.07
MBS5421813.1	hypoxanthine phosphoribosyltransferase [*Streptococcus salivarius*]	20.5	120.62	12.22	12	2	2	2	180	4.89

WP_183137226.1	DUF3290 family protein [*Streptococcus salivarius*]	16.9	120.42	16.78	38	2	2	2	149	8.76
HBJ22714.1	S-ribosylhomocysteine lyase [*Streptococcus salivarius*]	17.9	120.33	15.63	9	2	2	3	160	5.36
WP_013990672.1	cell division ATP-binding protein FtsE [*Streptococcus*]	26.0	120.22	21.30	11	5	5	5	230	8.97
MBS6732344.1	aquaporin family protein [*Streptococcus salivarius*]	30.9	119.85	7.64	26	2	2	2	288	6.32
WP_002890047.1	acetyl-CoA carboxylase, carboxyltransferase subunit beta [*Streptococcus salivarius*]	31.5	117.47	12.85	31	3	3	3	288	6.16
WP_002890091.1	4-hydroxy-tetrahydrodipicolinate reductase [*Streptococcus salivarius*]	27.7	116.25	14.51	30	3	3	4	255	5.12
WP_226972865.1	hypothetical protein [*Streptococcus salivarius*]	67.5	115.90	7.41	46	3	3	4	594	8.29
MBS7215126.1	histidine phosphatase family protein [*Streptococcus salivarius*]	22.4	114.12	27.00	57	4	4	4	200	5.85
WP_247914868.1	UDP-N-acetylmuramate--L-alanine ligase, partial [*Streptococcus salivarius*]	46.2	113.85	6.57	24	2	2	2	411	5.12
MBS4823121.1	superoxide dismutase SodA [*Streptococcus salivarius*]	14.6	111.55	26.72	24	3	3	3	131	5.02
WP_037598735.1	ParA family protein [*Streptococcus*]	30.2	109.72	19.93	21	4	4	5	276	4.93
WP_002889651.1	DeoR/GlpR family DNA-binding transcription regulator [*Streptococcus*]	28.5	109.29	10.59	4	2	2	2	255	6.27
WP_002883757.1	response regulator transcription factor [*Streptococcus*]	26.5	109.11	11.79	9	2	2	3	229	5.03
MBS4823128.1	GTP-binding protein [*Streptococcus salivarius*]	36.6	109.04	6.95	15	2	2	2	331	4.74
RGW02771.1	translation initiation factor IF-3 [*Streptococcus salivarius*]	19.9	108.52	19.54	8	4	4	4	174	9.76
MBS4822526.1	Asp-tRNA(Asn)/Glu-tRNA(Gln) amidotransferase subunit GatA [*Streptococcus salivarius*]	52.0	107.48	8.40	68	3	3	3	488	4.93
WP_002892202.1	ATP-binding cassette domain-containing protein [*Streptococcus salivarius*]	60.8	106.78	5.56	27	2	3	3	540	4.82
MBS4821773.1	carbamoyl phosphate synthase small subunit [*Streptococcus salivarius*]	40.1	106.68	6.63	16	2	2	2	362	6.13
WP_002884257.1	ABC transporter ATP-binding protein [*Streptococcus salivarius*]	27.5	105.93	13.99	30	3	3	3	243	5.11
MBS7053853.1	ATP-binding cassette domain-containing protein [*Streptococcus salivarius*]	16.6	105.63	24.16	38	3	3	3	149	9.26
MBS6885742.1	CotH kinase family protein [*Streptococcus salivarius*]	56.0	104.60	8.09	59	4	4	4	482	5.68
WP_002891470.1	glucosyltransferase domain-containing protein [*Streptococcus salivarius*]	56.0	104.45	8.35	1	4	4	4	491	9.22
MBS5423292.1	isoprenyl transferase [*Streptococcus salivarius*]	28.5	103.35	16.06	17	4	4	4	249	8.68
MBS6891548.1	DUF1129 domain-containing protein [*Streptococcus salivarius*]	25.1	103.08	13.00	10	2	2	4	223	9.25
WP_073688941.1	membrane protein insertase YidC, partial [*Streptococcus salivarius*]	24.3	102.88	10.14	49	2	2	2	217	9.54
MBS5181398.1	DUF1958 domain-containing protein [*Streptococcus salivarius*]	31.0	101.89	14.23	103	3	3	3	274	9.54
AEJ53725.1	TIGR03943 family protein [*Streptococcus salivarius* 57.I]	29.0	98.72	9.34	39	2	2	2	257	7.52
MBS4822645.1	nucleoside-triphosphate diphosphatase [*Streptococcus salivarius*]	35.7	98.63	8.02	42	2	2	2	324	5.00
WP_247915125.1	YebC/PmpR family DNA-binding transcriptional regulator, partial [*Streptococcus salivarius*]	23.7	98.41	11.01	12	2	2	2	218	4.40
WP_247922917.1	preprotein translocase subunit SecY, partial [*Streptococcus salivarius*]	34.0	98.32	11.86	14	3	3	3	312	9.14
MBS6319787.1	IMP dehydrogenase [*Streptococcus salivarius*]	48.0	98.21	6.90	17	3	3	3	449	5.92
AEJ52639.1	pur operon repressor PurR [*Streptococcus salivarius* 57.I]	28.5	98.20	7.60	14	2	2	2	263	5.10
WP_002890707.1	dTDP-4-dehydrorhamnose 3,5-epimerase family protein [*Streptococcus salivarius*]	22.4	97.19	18.27	32	3	3	3	197	5.03

WP_247914782.1	50S ribosomal protein L35, partial [*Streptococcus salivarius*]	6.7	96.89	32.14	2	3	3	5	56	12.60
WP_002894312.1	ATP-dependent zinc metalloprotease FtsH [*Streptococcus*]	72.5	95.78	7.13	1	2	5	6	659	5.52
WP_223896427.1	hypothetical protein [*Streptococcus salivarius*]	32.8	94.62	9.22	115	2	2	2	282	4.96
MBS6760764.1	30S ribosomal protein S18 [*Streptococcus salivarius*]	9.3	91.84	29.11	4	4	4	4	79	10.64
MBS7213645.1	acetyltransferase [*Streptococcus salivarius*]	20.4	91.39	14.36	49	2	2	2	188	8.21
MBS7214491.1	peptidase [*Streptococcus salivarius*]	29.5	87.39	9.56	92	2	2	2	293	4.93
WP_002883686.1	rRNA pseudouridine synthase [*Streptococcus*]	27.3	86.96	5.39	9	1	1	1	241	9.91
WP_002262412.1	50S ribosomal protein L33 [*Streptococcus*]	5.9	86.88	44.90	7	3	3	3	49	10.35
MBS7054683.1	phospho-N-acetylmuramoyl-pentapeptide-transferase [*Streptococcus salivarius*]	5.3	86.51	22.92	38	1	1	1	48	9.61
WP_002885786.1	50S ribosomal protein L28 [*Streptococcus*]	6.9	86.31	30.65	3	3	3	3	62	11.21
WP_201709461.1	isoleucine--tRNA ligase [*Streptococcus salivarius*]	105.8	83.55	4.61	87	4	4	4	932	4.97
WP_207185315.1	membrane protein insertase YidC [*Streptococcus salivarius*]	33.2	83.40	6.78	6	3	3	4	295	10.02
MBS5180676.1	DUF3042 family protein [*Streptococcus salivarius*]	3.8	82.55	28.13	5	2	2	4	32	10.61
WP_002884235.1	PH domain-containing protein [*Streptococcus salivarius*]	14.1	81.84	34.92	19	4	4	4	126	5.55
MBS7214878.1	inositol monophosphatase family protein [*Streptococcus salivarius*]	6.1	81.37	22.22	41	1	1	1	54	5.01
WP_038676984.1	phage holin [*Streptococcus salivarius*]	8.5	80.71	10.53	2	1	1	2	76	7.25
WP_247914817.1	ABC transporter permease subunit, partial [*Streptococcus salivarius*]	20.2	80.71	12.23	9	2	2	2	188	9.31
WP_002886340.1	alkyl hydroperoxide reductase subunit C [*Streptococcus*]	20.5	80.58	12.90	21	3	3	3	186	4.70
WP_002891688.1	hypothetical protein [*Streptococcus*]	29.1	78.91	11.60	32	3	3	3	250	5.21
WP_002883992.1	glucose-1-phosphate thymidylyltransferase RfbA [*Streptococcus*]	32.0	77.87	11.07	13	3	3	3	289	4.78
WP_073922228.1	ABC transporter ATP-binding protein, partial [*Streptococcus salivarius*]	24.8	77.53	6.28	39	1	1	1	223	7.20
WP_002889668.1	tagatose-bisphosphate aldolase [*Streptococcus*]	36.5	76.70	7.06	4	2	2	2	326	4.84
WP_002892326.1	non-ribosomal peptide synthetase [*Streptococcus salivarius*]	210.8	76.68	1.19	2	2	2	2	1856	5.17
ARC49259.1	UDP-N-acetylmuramate dehydrogenase [*Streptococcus salivarius*]	33.1	75.79	7.00	54	2	2	2	300	6.57
WP_073922347.1	ABC transporter permease, partial [*Streptococcus salivarius*]	34.8	74.06	4.85	32	2	2	3	330	8.69
AEJ52905.1	spermidine/putrescine import ATP-binding protein PotA [*Streptococcus salivarius* 57.I]	42.4	72.53	5.66	16	2	2	2	371	4.65
WP_002892335.1	ACP S-malonyltransferase [*Streptococcus*]	32.7	72.19	5.72	1	2	2	2	297	4.91
WP_064524928.1	transcriptional regulator NrdR [*Streptococcus salivarius*]	18.3	71.83	13.92	10	2	2	2	158	6.79
WP_064526629.1	3-hydroxyacyl-ACP dehydratase FabZ, partial [*Streptococcus salivarius*]	14.1	71.72	33.07	3	4	4	4	127	7.40
MBS7214705.1	YbbR-like domain-containing protein [*Streptococcus salivarius*]	23.7	69.90	12.89	45	2	2	2	225	8.25
WP_247922456.1	Hsp70 family protein, partial [*Streptococcus salivarius*]	37.3	69.81	10.45	27	3	3	3	335	5.25
WP_195187947.1	formate/nitrite transporter family protein, partial [*Streptococcus salivarius*]	27.5	68.79	8.06	51	2	2	2	248	8.25
MBS5423388.1	amino acid ABC transporter permease [*Streptococcus salivarius*]	22.3	68.25	8.46	11	2	2	2	201	9.07
HBJ22709.1	glutathione-disulfide reductase [*Streptococcus salivarius*]	48.7	67.58	4.22	66	2	2	3	450	5.62
MBS7215167.1	Rrf2 family transcriptional regulator [*Streptococcus salivarius*]	16.0	67.55	8.05	12	1	1	1	149	9.01
WP_155213791.1	aldo/keto reductase, partial [*Streptococcus salivarius*]	31.5	67.07	8.19	78	2	2	2	281	6.04

WP_247922777.1	GTP-binding protein, partial [*Streptococcus salivarius*]	20.8	66.74	5.32	38	1	1	1	188	4.55
MBS4923571.1	DNA-directed RNA polymerase subunit omega [*Streptococcus salivarius*]	11.2	66.66	26.00	10	3	3	4	100	6.57
MBS7213687.1	PTS mannose/fructose/sorbose transporter subunit IIC [*Streptococcus salivarius*]	27.3	66.64	4.14	5	1	1	1	266	4.70
MBS4823607.1	BCCT family transporter [*Streptococcus salivarius*]	44.9	66.51	3.41	17	1	1	1	410	9.48
WP_002883720.1	branched-chain amino acid ABC transporter permease [*Streptococcus*]	33.9	65.62	6.65	12	2	2	2	316	9.54
MBS7214967.1	carbamoyl-phosphate synthase large subunit [*Streptococcus salivarius*]	99.6	65.39	4.76	90	4	4	4	904	4.78
MBS7214802.1	YSIRK signal domain/LPXTG anchor domain surface protein [*Streptococcus salivarius*]	54.9	65.08	2.53	84	1	1	1	514	6.06
KJU94562.1	heat shock protein GrpE [*Streptococcus salivarius*]	19.9	64.97	13.29	44	2	2	2	173	4.70
MBS7214875.1	esterase family protein [*Streptococcus salivarius*]	25.4	64.34	5.02	18	1	1	2	219	5.40
AEJ54106.1	conserved hypothetical protein [*Streptococcus salivarius* 57.I]	11.7	63.86	23.58	19	3	3	3	106	9.94
WP_002884978.1	response regulator YycF [*Streptococcus*]	27.0	63.82	8.94	11	2	2	2	235	5.19
MBS6733527.1	DUF948 domain-containing protein [*Streptococcus salivarius*]	7.7	63.71	14.67	12	1	1	1	75	9.32
WP_073690614.1	glycerol-3-phosphate 1-O-acyltransferase PlsY, partial [*Streptococcus salivarius*]	21.8	63.66	7.00	39	1	1	1	200	9.64
AAC43563.1	urease gamma subunit [*Streptococcus salivarius*]	11.5	63.55	17.48	7	3	3	3	103	6.30
WP_142236616.1	transcriptional repressor, partial [*Streptococcus salivarius*]	9.8	62.99	25.58	23	2	2	2	86	4.92
MBS6120384.1	aspartate kinase [*Streptococcus salivarius*]	41.9	62.70	9.52	33	3	3	3	378	4.94
MBF1717945.1	glutamine-hydrolyzing GMP synthase [*Streptococcus salivarius*]	56.7	61.50	3.68	40	2	2	2	516	4.94
WP_073690484.1	hypothetical protein, partial [*Streptococcus salivarius*]	42.0	61.09	3.12	79	1	1	1	385	5.22
MBF1719041.1	ABC transporter ATP-binding protein [*Streptococcus salivarius*]	45.3	59.88	3.95	76	2	2	2	405	5.39
MBS6886067.1	ABC transporter ATP-binding protein [*Streptococcus salivarius*]	22.8	59.83	14.71	16	3	3	3	204	7.05
MBS6121086.1	ABC transporter permease [*Streptococcus salivarius*]	19.8	59.75	4.81	19	1	1	1	187	4.84
WP_195413864.1	CPBP family intramembrane metalloprotease [*Streptococcus salivarius*]	23.4	59.03	3.79	4	1	1	1	211	9.67
WP_247922959.1	lipoate--protein ligase family protein, partial [*Streptococcus salivarius*]	17.6	58.52	6.41	41	1	1	1	156	6.09
MBS6761570.1	acyl carrier protein [*Streptococcus salivarius*]	8.4	58.36	14.86	6	1	1	1	74	3.87
WP_002885996.1	ribosome biogenesis GTP-binding protein YihA/YsxC [*Streptococcus*]	22.8	58.32	12.06	17	2	2	2	199	8.66
WP_073922203.1	urease accessory protein UreG, partial [*Streptococcus salivarius*]	16.8	58.09	5.23	11	1	1	1	153	4.88
MBS7214217.1	pyruvate formate lyase-activating protein [*Streptococcus salivarius*]	30.5	57.66	10.90	9	2	2	2	266	6.04
AEJ52680.1	Mg2+ and Co2+ transporter [*Streptococcus salivarius* 57.I]	35.9	56.31	3.22	27	1	1	1	311	4.86
WP_155213573.1	DUF1372 family protein [*Streptococcus salivarius*]	10.4	56.28	17.89	5	1	1	1	95	8.68
RGW02733.1	hypothetical protein [*Streptococcus salivarius*]	10.4	55.82	17.39	8	2	2	2	92	5.68
WP_037597359.1	tRNA dihydrouridine synthase DusB [*Streptococcus salivarius*]	35.8	52.62	8.62	25	2	2	2	325	6.20
WP_064526452.1	acetolactate synthase small subunit, partial [*Streptococcus salivarius*]	13.8	51.64	12.80	13	2	2	2	125	7.27
MBS6121021.1	amino acid permease [*Streptococcus salivarius*]	47.3	51.04	2.12	47	1	1	2	424	9.36
MBS7054875.1	ATP-dependent Clp protease proteolytic subunit [*Streptococcus salivarius*]	20.4	50.76	12.90	11	2	2	2	186	4.96
WP_014635141.1	type Z 30S ribosomal protein S14 [*Streptococcus salivarius*]	6.6	50.23	28.57	3	2	2	2	56	10.35

WP_073689559.1	rhodanese-related sulfurtransferase, partial [*Streptococcus salivarius*]	27.2	47.56	5.93	50	1	1	1	236	5.01
WP_002886207.1	CsbD family protein [*Streptococcus*]	7.1	47.13	16.42	9	1	1	1	67	6.77
WP_201710930.1	CTP synthase, partial [*Streptococcus salivarius*]	31.1	46.05	5.71	34	1	1	1	280	5.64
WP_223896526.1	cadmium resistance transporter, partial [*Streptococcus salivarius*]	5.6	45.60	26.53	32	1	1	1	49	6.51
MBS6120897.1	phosphotransferase family protein [*Streptococcus salivarius*]	24.3	45.20	3.79	7	1	1	1	211	6.87
WP_096832747.1	P-loop NTPase fold protein [*Streptococcus salivarius*]	74.1	44.99	0.96	1	1	1	3	623	8.07
MBS6119901.1	glycosyltransferase family 2 protein [*Streptococcus salivarius*]	16.9	44.99	15.49	23	2	2	2	142	10.04
MBS6732157.1	CDP-diacylglycerol--glycerol-3-phosphate 3-phosphatidyltransferase [*Streptococcus salivarius*]	19.9	44.88	8.89	16	2	2	2	180	9.03
MBS7054767.1	DUF1846 family protein [*Streptococcus salivarius*]	28.8	43.59	5.77	44	1	1	1	260	5.57
MBS4922658.1	DUF805 domain-containing protein [*Streptococcus salivarius*]	14.0	43.45	11.67	40	2	2	2	120	9.16
AEJ52879.1	ribonuclease BN [*Streptococcus salivarius* 57.I]	35.4	43.27	5.75	31	1	1	1	313	9.74
WP_002892266.1	ABC transporter ATP-binding protein [*Streptococcus salivarius*]	34.4	43.22	4.29	4	2	2	2	303	8.02
MBS4823402.1	phosphocarrier protein HPr [*Streptococcus salivarius*]	8.9	43.14	11.49	3	1	1	1	87	5.05
MBS6760419.1	DUF956 family protein [*Streptococcus salivarius*]	15.2	42.97	17.19	20	2	2	2	128	10.26
WP_073690295.1	tryptophan synthase subunit alpha, partial [*Streptococcus salivarius*]	18.5	42.73	10.65	49	1	1	1	169	5.01
WP_002885967.1	DUF1827 family protein [*Streptococcus*]	11.7	42.64	8.00	2	1	1	1	100	6.95
WP_084913461.1	DUF87 domain-containing protein, partial [*Streptococcus salivarius*]	45.8	41.23	2.94	3	1	1	1	408	6.46
MBS4822178.1	CppA family protein [*Streptococcus salivarius*]	28.8	40.45	5.18	19	1	1	1	251	4.53
MBS6120594.1	DegV family protein [*Streptococcus salivarius*]	14.5	40.12	6.02	82	1	1	1	133	9.36
MBS4822338.1	TetR/AcrR family transcriptional regulator [*Streptococcus salivarius*]	22.5	40.03	5.05	25	1	1	1	198	6.68
MBS6890550.1	bacteriocin immunity protein [*Streptococcus salivarius*]	11.3	39.82	12.24	16	1	1	1	98	6.11
MBS6732137.1	acid-activated urea channel [*Streptococcus salivarius*]	7.0	39.73	10.61	12	1	1	1	66	5.72
MBS6885736.1	UDP-N-acetylglucosamine 1-carboxyvinyltransferase [*Streptococcus salivarius*]	31.6	39.27	3.00	35	1	1	1	300	5.03
MBS6531340.1	NADPH-dependent 7-cyano-7-deazaguanine reductase QueF [*Streptococcus salivarius*]	17.0	37.95	6.29	12	1	1	1	143	5.15
MBF1718323.1	site-specific integrase [*Streptococcus salivarius*]	48.2	37.93	1.43	1	1	1	1	421	9.60
CCB96178.1	response regulator of the LytR/AlgR family [*Streptococcus salivarius* JIM8777]	27.3	37.92	3.80	19	1	1	1	237	7.97
HBJ24245.1	type I 3-dehydroquinate dehydratase [*Streptococcus salivarius*]	25.5	37.38	5.78	42	1	1	1	225	4.67
WP_073922287.1	pyrimidine-nucleoside phosphorylase, partial [*Streptococcus salivarius*]	45.1	36.77	2.65	66	1	1	1	415	5.06
WP_064523977.1	hypothetical protein [*Streptococcus salivarius*]	11.3	36.41	6.38	1	1	1	1	94	5.31
WP_073687230.1	ATP-binding cassette domain-containing protein [*Streptococcus*]	27.0	36.36	2.46	1	1	1	1	244	9.09
WP_231593871.1	hypothetical protein [*Streptococcus salivarius*]	6.0	36.18	11.32	2	1	1	1	53	10.13
WP_000598356.1	DUF3173 domain-containing protein [*Streptococcus*]	7.0	36.06	9.38	2	1	1	1	64	8.54
WP_142236428.1	tRNA uridine-5-carboxymethylaminomethyl(34) synthesis enzyme MnmG, partial [*Streptococcus salivarius*]	27.8	36.02	3.13	48	1	1	1	256	5.52
WP_247914645.1	Asp23/Gls24 family envelope stress response protein, partial [*Streptococcus salivarius*]	8.0	35.97	11.69	5	1	1	1	77	4.96
WP_195310560.1	hypothetical protein [*Streptococcus salivarius*]	55.6	35.75	1.30	3	1	1	1	462	7.84

WP_013990691.1	UDP-N-acetylglucosamine 2-epimerase (non-hydrolyzing) [*Streptococcus*]	43.1	35.19	1.57	2	1	1	1	383	5.90
WP_225791458.1	tape measure protein [*Streptococcus salivarius*]	33.3	35.19	3.14	8	1	1	1	318	9.13
AEJ52985.1	organic phosphate binding cyth family protein [*Streptococcus salivarius* 57.I]	13.6	34.92	6.72	26	1	1	1	119	5.10
MBS6531707.1	acetyl-CoA carboxylase carboxyl transferase subunit alpha [*Streptococcus salivarius*]	16.0	34.34	5.44	23	1	1	1	147	7.43
WP_179968688.1	efflux RND transporter periplasmic adaptor subunit, partial [*Streptococcus salivarius*]	29.6	33.85	5.13	71	1	1	1	273	4.77
MBS6731867.1	ABC transporter permease [*Streptococcus salivarius*]	40.1	33.84	2.32	13	1	1	1	345	10.02
MBS6885364.1	30S ribosomal protein S20 [*Streptococcus salivarius*]	7.6	33.65	17.14	7	1	1	1	70	10.29
MBF1719263.1	bifunctional UDP-N-acetylglucosamine diphosphorylase/glucosamine-1-phosphate N-acetyltransferase GlmU [*Streptococcus salivarius*]	45.1	33.42	1.92	53	1	1	1	416	5.40
WP_241672536.1	cell wall-active antibiotics response protein [*Streptococcus salivarius*]	15.3	33.35	5.11	70	1	1	1	137	8.97
WP_002885550.1	DUF1149 family protein [*Streptococcus*]	14.4	31.86	20.97	5	2	2	2	124	4.75
AMB83404.1	hypothetical protein [*Streptococcus salivarius*]	9.1	31.41	10.67	28	1	1	1	75	9.48
WP_247915103.1	aldo/keto reductase, partial [*Streptococcus salivarius*]	30.3	31.12	5.86	85	2	2	2	273	4.81
MBS6761620.1	ABC-F family ATP-binding cassette domain-containing protein [*Streptococcus salivarius*]	37.7	30.89	3.34	40	1	1	1	329	5.19
WP_002883656.1	preprotein translocase subunit SecE [*Streptococcus*]	7.0	30.02	12.07	2	1	1	1	58	8.62
WP_195401316.1	ImmA/IrrE family metallo-endopeptidase [*Streptococcus salivarius*]	34.2	29.03	2.79	3	2	2	4	287	6.89
BAE97498.1	DNA gyrase subunit B, partial [*Streptococcus salivarius*]	33.4	28.97	6.67	39	2	2	2	300	7.43
AEJ54319.1	regulatory protein spx [*Streptococcus salivarius* 57.I]	12.9	28.40	7.96	2	1	1	1	113	6.14
MBS6273035.1	ammonia-dependent NAD(+) synthetase [*Streptococcus salivarius*]	30.0	28.02	6.23	48	2	2	2	273	5.25
AEJ53397.1	regulatory protein spx [*Streptococcus salivarius* 57.I]	13.9	27.95	9.32	12	1	1	1	118	5.05
WP_073922337.1	ketoacyl-ACP synthase III, partial [*Streptococcus salivarius*]	33.0	27.61	2.59	50	1	1	1	309	4.78
WP_241525105.1	alpha-amylase family glycosyl hydrolase, partial [*Streptococcus salivarius*]	39.0	27.59	2.70	65	1	1	1	333	5.25
MBS6119481.1	DNA-dependent RNA polymerase auxiliary subunit epsilon family protein [*Streptococcus salivarius*]	9.1	27.35	9.21	10	1	1	1	76	4.68
MBS5182041.1	pyridoxal 5'-phosphate synthase lyase subunit PdxS [*Streptococcus salivarius*]	19.7	25.61	5.52	27	1	1	1	181	6.13
